# Visualizing Evolutionary Relationships of Multidomain Proteins: An Example from Receiver (REC) Domains of Sensor Histidine Kinases in the *Candidatus* Maribeggiatoa str. Orange Guaymas Draft Genome

**DOI:** 10.3389/fmicb.2016.01780

**Published:** 2016-11-14

**Authors:** Barbara J. MacGregor

**Affiliations:** Department of Marine Sciences, University of North Carolina-Chapel HillChapel Hill, NC, USA

**Keywords:** Orange Guaymas Maribeggiatoa, Beggiatoaceae, Cyanobacteria, sensor histidine kinases, recognition (REC) domains, multidomain proteins, horizontal gene transfer

## Abstract

For multidomain proteins, evolutionary changes may occur at the domain as well as the whole-protein level. An example is presented here, with suggestions for how such complicated relationships might be visualized. Earlier analysis of the *Candidatus* Maribeggiatoa str. Orange Guaymas (BOGUAY; Gammaproteobacteria) single-filament draft genome found evidence of gene exchange with the phylogenetically distant Cyanobacteria, particularly for sensory and signal transduction proteins. Because these are modular proteins, known to undergo frequent duplication, domain swapping, and horizontal gene transfer, a single domain was chosen for analysis. Recognition (REC) domains are short (~125 amino acids) and well conserved, simplifying sequence alignments and phylogenetic calculations. Over 100 of these were identified in the BOGUAY genome and found to have a wide range of inferred phylogenetic relationships. Two sets were chosen here for detailed study. One set of four BOGUAY ORFs has closest relatives among other Beggiatoaceae and Cyanobacteria. A second set of four has REC domains with more mixed affiliations, including other Beggiatoaceae, several sulfate-reducing Deltaproteobacteria and Firmicutes, magnetotactic Nitrospirae, one *Shewanella* and one *Ferrimonas* strain (both Gammaproteobacteria), and numerous *Vibrio vulnificus* and *V. navarrensis* strains (also Gammaproteobacteria). For an overview of the possible origins of the whole proteins and the surrounding genomic regions, color-coded BLASTP results were produced and displayed against cartoons showing protein domain structure of predicted genes. This is suggested as a visualization method for investigation of possible horizontally transferred regions, giving more detail than scans of DNA composition and codon usage but much faster than carrying out full phylogenetic analyses for multiple proteins. As expected, most of the predicted sensor histidine kinases investigated have two or more segments with distinct BLASTP affiliations. For the first set of BOGUAY ORFs, the flanking regions were also examined, and the results suggest they are embedded in genomic stretches with complex histories. An automated method of creating such visualizations could be generally useful; a wish list for its features is given.

## Introduction

The *Candidatus* Maribeggiatoa str. Orange Guaymas (BOGUAY) single-filament draft genome contains potential mobile genetic elements of several types (introns, inteins, and possible excision elements) with close relatives among the phylogenetically distant Cyanobacteria, suggesting a history of genetic exchange between these groups (MacGregor et al., 2013c). As identified by the top five BLASTP matches, the largest single category of potentially exchanged genes was for sensory and signal transduction proteins, raising the question of what environmental conditions these might respond to, what other genes they might interact with, and which lineages may have contributed (or received) which functions.

The multidomain nature of signal transduction proteins complicates phylogenetic inferences. Domains appear to be swapped at a high rate relative to overall genome evolution both within and between species (reviewed in Capra and Laub, 2012; Salazar and Laub, 2015), presumably allowing a range of regulatory adaptations to be tested within a population. The REC (recognition) domain was selected for this analysis, being short (~125 amino acids) and easily aligned. In the studied cases, REC domains are phosphorylated by a histidine kinase upstream in a signaling chain, which may be on the same or a different protein. They may change conformation or dimerize, and interact with an element downstream in the chain (reviewed in Casino et al., 2010).

Understanding the evolution of multidomain signal transduction proteins is a daunting task, but could yield insights into the regulatory adaptations of bacterial species to their local environments. For the Beggiatoaceae, these include shallow hypersaline ponds, sulfidic seeps in freshwater lakes, and sulfidic deep sea sediments at vents, seeps, and the western continental margins of Africa and South America. These large vacuolated bacteria are sometimes found in close association with other species in microbial mats, and can be covered with epibionts (e.g., Fliss, 2014; Flood et al., 2016), suggesting opportunities for gene transfer. The individual cells or filaments may have higher than usual genome copy numbers (Angert, 2012), which could make them better able to carry out and tolerate genetic rearrangements of various sorts; these points are the topic of current research in several labs.

Analysis of such multidomain proteins would greatly benefit from phylogenetically nested domain-, protein-, local neighborhood-, and whole-genome visualization tools linked to the ever-expanding sequence database. Presented here is a detailed look at two sets of REC domains, illustrating both their possible history and how such visualizations might appear.

## Materials and methods

### Available genomes of *Beggiatoaceae* and related large sulfur bacteria

Few *Beggiatoaceae* and related large sulfur gammaproteobacteria have as yet been sequenced, and fewer still are in cultivation; their classification is still in progress (Salman et al., 2011, 2013). There are complete or near-complete genome sequences for *Beggiatoa alba* B18LD (BegalDRAFT; Lucas et al., unpublished), *Beggiatoa leptomitiformis* D-420 (Fomenkov et al., 2015), *Thioploca ingrica* (THII; Kojima et al., 2015), and *Cand*. Maribeggiatoa “Orange Guaymas” (MacGregor et al., 2013a,b,c); a partial sequence for *Cand*. Thiomargarita nelsonii (Mußmann et al., unpublished); and one partial (*Cand*. Isobeggiatoa sp. PS; BGP) and one very partial (*Cand*. Parabeggiatoa sp. SS; BGS) genome for two filaments collected from Baltic Sea harbor sediment (Mußmann et al., 2007). By 16S rRNA phylogeny, *B. alba* is in a separate clade from the rest (Salman et al., 2013; *B. leptomitiformis* had not been sequenced when these names were proposed).

### Naming conventions

Orange Guaymas *Cand*. Maribeggiatoa filament (abbreviated BOGUAY) draft genome open reading frames (ORFs) are referred to either by their IMG/ER locus tag (e.g., BOGUAY_1733) or by contig and locus tag (e.g., BOGUAY 00362_1733). *Cand*. Isobeggiatoa sp. PS, *Cand*. Parabeggiatoa sp. SS, and *B. alba* B18LD locus tags begin with BGP, BGS, or BegalDRAFT, respectively. ORFs from other species are referred to by GenBank accession number or, for those whose chromosomal location was checked, by IMG locus tags. In phylogenetic trees, numbers in parentheses (e.g., 5–119) indicate the amino acid residues used in the REC sequence alignment. At present, the same sequence may have two or three different accession numbers in GenBank and two different locus tags in IMG/ER; an attempt was made to be consistent, but because sequences were downloaded at different times, designations within a species may have more than one form.

Where multiple REC domains occur within a single putative BOGUAY protein, letter designations were used for the proteins and numbers for REC domains, in order of their position [e.g., BOGUAY 00362_1733 contains REC domains N1 (amino acids 8-120) and N2 (amino acids 148-260)].

### Phylogenetic reconstructions

Putative REC domains were identified in the BOGUAY genome within IMG/ER (www.jgi.doe.gov) by searching for relevant keywords and COG, KOG, and pfam numbers. The predicted amino acid sequences were then used to search the NCBI database with BLASTP, and the highest-scoring matching regions downloaded. One hundred hits per sequence were taken initially, but due to extensive overlap between sets, this was decreased to 50 for most searches. Because few genomic sequences are so far available for the *Beggiatoaceae*, there is no chance for search results to be dominated by very close relatives, as happens for example with pathogens. The final database included 4672 amino acid sequences, some of which derived from putative proteins with two or more separate REC domains. The REC domains were aligned in MEGA5 (Tamura et al., 2011) using MUSCLE (Edgar, 2004) and small adjustments made manually. Where only partial domains were retrieved by BLAST, upstream and/or downstream residues were added from the GenBank entries. The aligned sequences were exported to ARB (Ludwig et al., 2004), an initial guide tree computed by neighbor joining, and subtrees centered on BOGUAY sequences computed first by neighbor joining and then with RAxML rapid bootstrapping (Stamatakis, 2006) using a random initial tree, the PROTMIX or more recently implemented PROTGAMMA rate distribution and WAG amino acid substitution models, empirical amino acid frequencies, and branch optimization. The final species composition of most trees is the result of several RAxML and neighbor-joining runs, with consistently outlying or unstable branches removed. The final set of 35 RAxML trees included 2254 REC domain sequences. The trees are numbered in order of the occurrence of the majority of their species in the initial neighbor-joining guide tree. The three trees described here (9, 19, and 36) were updated by new BLASTP searches in Spring 2016. Sequences and alignments of the REC domains in each tree are provided in the Supplemental Files. The domain structure of all proteins was predicted using the Conserved Domain Database (CDD, Marchler-Bauer et al., 2011). Protein domain abbreviations are those used by the CDD (summarized in Supplemental Table 1). Predicted chromosomal arrangements and ORF annotations were retrieved from IMG/ER.

## Results

Three REC-domain phylogenetic trees will be discussed here, numbered according to their position in the initial guide tree. Tree 36 includes primarily ORFs predicted to contain a single REC domain internal to a protein, closely related to Cyanobacterial sequences. Trees 19 and 9 include REC domains typically found together at the end of an ORF, often with a third partial or complete REC domain between them, and more diverse affiliations. Their inferred phylogenies are considered below, along with those of the proteins containing them and the surrounding genomic neighborhoods.

### Tree 36: cyanobacterial affiliations

#### Inferred REC domain phylogeny

**Figure 1:**
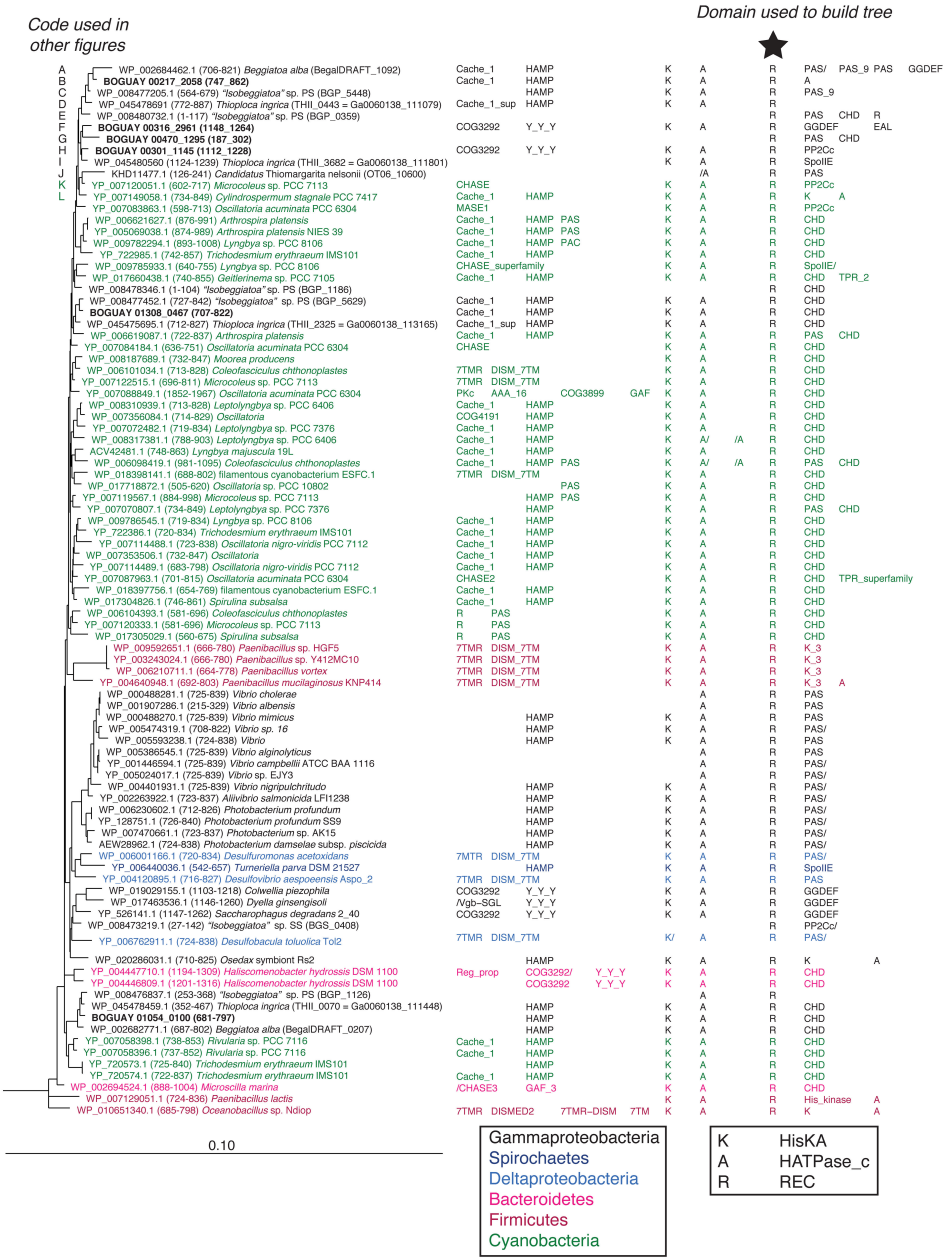
**REC domain Tree 36**. The tree was produced using RAxML rapid bootstrapping (Stamatakis, 2006) as implemented in ARB (Ludwig et al., 2004), using a random initial tree, the PROTGAMMA rate distribution and WAG amino acid substitution models, empirical amino acid frequencies, and branch optimization. Six *Clostridia* sequences were used to root the tree. Domain composition is from CDD (Marchler-Bauer et al., 2011). See Supplemental Table 1 for definition of domain abbreviations.

Tree 36 (Figure 1) includes three clusters of predicted Beggiatoaceae REC domains with cyanobacterial sequences as closest affiliates. More distantly related are several Spirochaete, Deltaproteobacteria, Bacteroidetes, and Firmicute sequences. The domain structures of the predicted proteins including them generally have one or two domains upstream of the HisKA-HATPase_c-REC (abbreviated KA-R) core, and one or occasionally two domains downstream of this, with a range of predicted sensory (e.g., Cache_1, PAS) and signal transduction (e.g., HAMP, CHD) roles.

#### Inferred phylogeny of other domains

**Figure 2:**
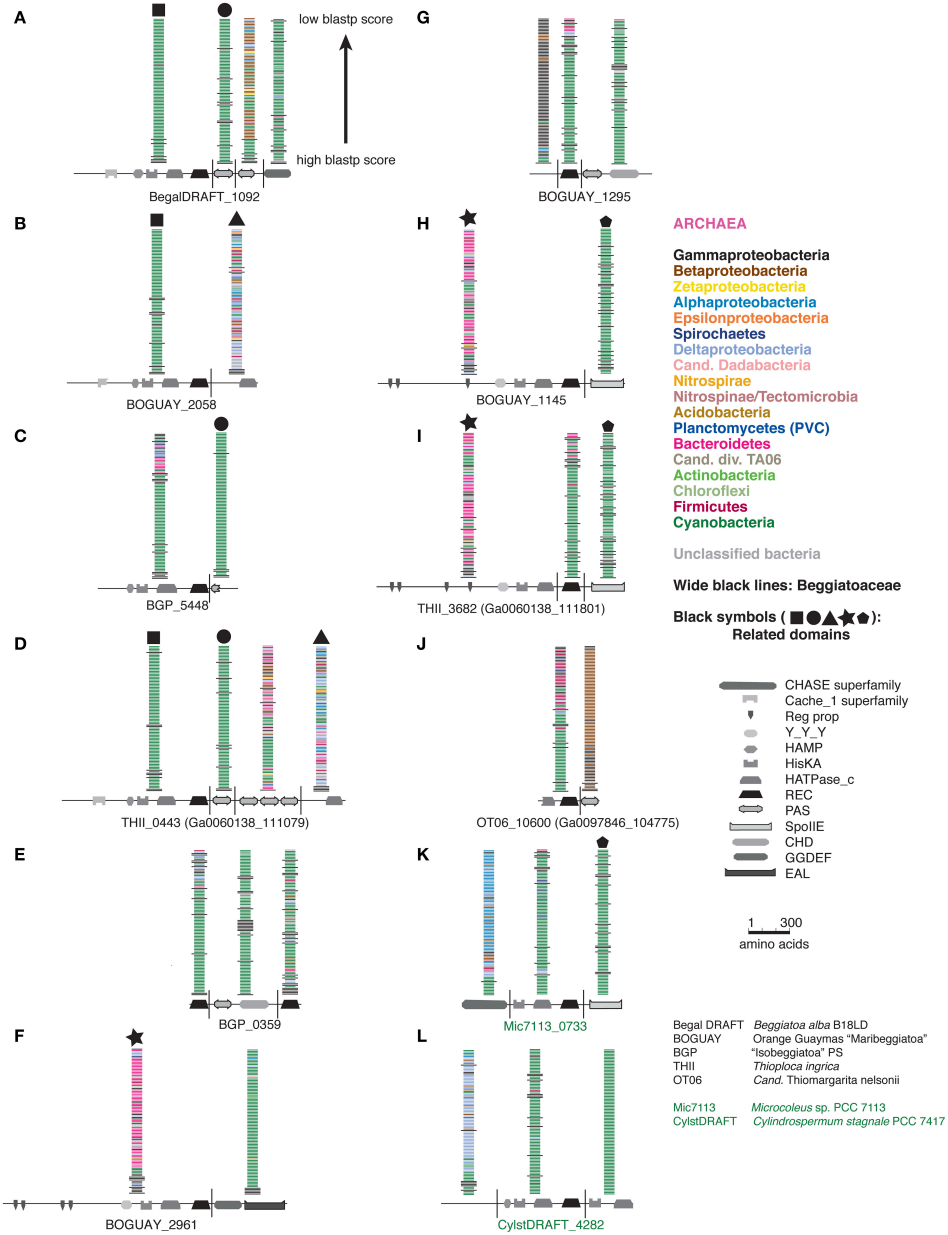
**Color-coded Tree 36 BLASTP results with cartoon representations of domain compositions**. Each BLASTP hit is represented by a single bar, colored by phylum. Beggiatoaceae are indicated by wide black bars, other Gammaproteobacteria by normal ones. Black symbols at the top of some BLASTP results indicate domains with similar affiliations, identified by looking at the full species lists (not shown).

A full phylogenetic analysis of each domain of each protein would be very time-consuming, so a quicker method was sought for an overview, shown here for the first 12 sequences in the tree (coded A through L). These include 10 Beggiatoaceae and two Cyanobacteria sequences. First, BLASTP searches were used to identify possible separately derived regions within each predicted protein (Supplemental Figure 1); all 12 appear to be mosaics. The matches were then illustrated as phylogenetically color-coded bars, displayed against the predicted domain structures (Figure 2). Wide black bars were used to distinguish Beggiatoaceae from other Gammaproteobacteria.

All but one REC domain is within a segment with primarily Cyanobacterial BLASTP hits, as expected. The exception is BOGUAY_2961 (Figure 2F): the REC domain is at the downstream end of a segment with a high proportion of Bacteroidetes matches (although Cyanobacteria are still among the highest-scoring), immediately upstream of a Cyanobacteria-related segment. All but the two shortest sequences (BGP_5448 and BGP_0359) also have at least one segment with substantially different associations. Consideration of the species lists themselves (not shown) identified similar segments in different predicted proteins, indicated by large black symbols; for example, BOGUAY_2058 (Figure 2B) and THII_0443 (Figure 2D) both have terminal HATPase_c domains with similar mixed, particularly Deltaproteobacterial hits. This is an unusual position for this domain, more often found between a histidine kinase and REC domain. Only one set of matches (Figure 2G) is dominated by Gammaproteobacterial sequences, and these do not include any Beggiatoaceae except a “self” hit, suggesting that this segment too may have been exchanged horizontally.

**Figure 3:**
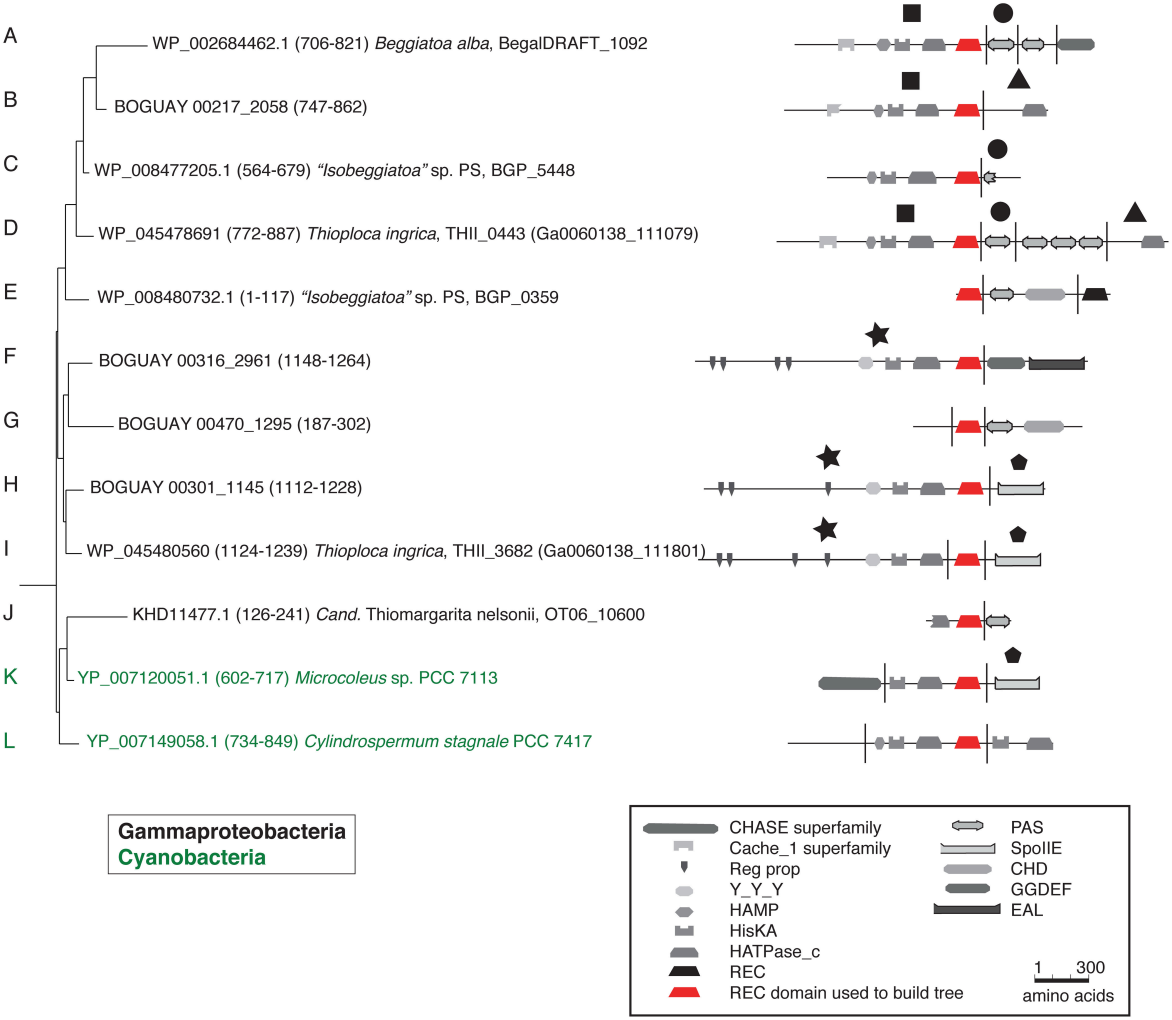
**Detail of REC domain Tree 36 showing BLASTP-predicted related predicted protein segments**.

Putting these cartoons in the context of the REC domain phylogenetic tree (Figure 3), hypotheses about how these predicted proteins may have been assembled can be suggested. For example, two *Beggiatoaceae* have a predicted terminal SpoIIE domain similar to that of the Cyanobacterial Mic7113_0733 (Figures 3H,I,K; pentagon symbol). All matches to all three segments are from Cyanobacteria, Beggiatoaceae, or (for THII_3682 only) unclassified bacteria. The Beggiatoaceae matches for this domain are dispersed rather than clustered (Figures 2H,I,K), suggesting that (alone or as part of some protein) it may have been introduced from Cyanobacteria to an ancestral Beggiatoaceae species, and then diverged within these. Alternatively, there could have been several transfers from different Cyanobacteria to different Beggiatoaceae, but this seems less likely.

#### BLASTP relatives of neighboring predicted proteins

**Figure 4:**
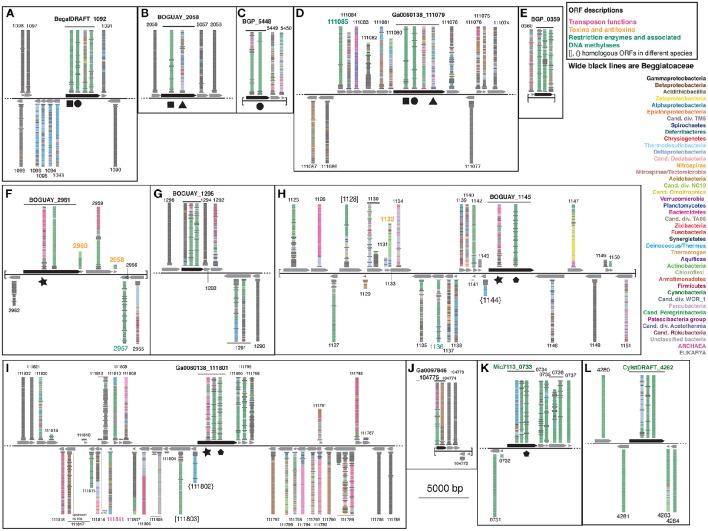
**Color-coded BLASTP results for genome neighborhoods of selected Tree 36 predicted sensor histidine kinases**. Possible transfer-associated functions are indicated. For a complete list of ORFs, see Supplemental Table 2.

To search for the boundaries of possible mobile elements that could have introduced sensor proteins to the Beggiatoaceae genomes, the BLASTP visualizations were continued to either side of the ORFs encoding the putative REC domain-containing proteins until either sequences with only Gammaproteobacterial matches or the end of a contig was reached (Figure 4; see Supplemental Table 2 for ORF descriptions). Nearly all of the Beggiatoaceae ORFs are part of regions with apparently complex histories. The illustration for a vertically transmitted gene is expected to resemble that of *T. ingrica* Ga0060138_111077 (Figure 4D), with Beggiatoaceae sequences first (wide black bars) and then strictly Gammaproteobacteria, for which there are many more than 100 (the number of bars) sequenced genomes available. This is only suggestive evidence; proof would require a closer look at the species identities and sequences.

The *B. alba* region in Figure 4A appears to have the simplest exchange history. On the bottom strand are four consecutive ORFs (BegalDRAFT 1093-1096) whose closest affiliations are to just one other Beggiatoaceae sequence, followed by a mixture of species with Alphaproteobacteria predominating. These are predicted to encode a cobalt-nickel transporter (Supplemental Table 2A), a likely candidate for gene transfer, since heavy-metal resistance is often carried on mobile elements (reviewed in Bouzat and Hoostal, 2013); the simplest interpretation would be that it was acquired by the *B. alba* lineage after its divergence from the other Beggiatoaceae, although differential retention is also a possibility. Immediately upstream of this, on the opposite strand, is the predicted REC domain protein gene (BegalDRAFT_1092), with primarily Cyanobacterial affiliations. All four regions of this ORF have sporadically distributed Beggiatoaceae matches (wide black bars), suggesting that they may have diverged since their acquisition by a common ancestor. These two possibly transferred segments are flanked by at least several ORFs (as far as checked) with primarily Gammaproteobacterial BLASTP hits.

Most of the other Beggiatoaceae regions illustrated appear to have more complex histories. A few include predicted genes that may record at least part of the transfer mechanism: toxins and an antitoxin (Figures 4F,H), a transposase (Figure 4I), and two restriction endonucleases and a possible associated methylase (Figures 4D,F,H). BOGUAY_1145 and *T. ingrica* Ga0060138_111801 (Figures 4H,I) appear to be related by rearrangement, with homologous ORFs (Ga0060138_111802, BOGUAY_1144) encoding putative hypothetical proteins immediately upstream of very similar REC domain protein genes. The ORF just downstream of this one in *T. ingrica* (Ga0060138_111803) also has a BOGUAY homolog (BOGUAY_1128), but it is further from the REC domain protein, and this gene pair has less similar affiliations, although both are annotated as CHAT domain (“Caspase HetF Associated with Tprs”; possible peptidases) proteins with N-terminal tetratricopeptide repeats (groups TPR_16 and TPR_12, respectively). Two of the Cyanobacterial REC domain proteins in Tree 36 (Figure 1: WP_0176660438, TPR_2; YP_007087963, TPR_16) are themselves annotated with C-terminal tetratricopeptide domains. Such repeats suggest a possible mechanism for recombination, but if there was a repeat-mediated event, these sequences appear to have diverged considerably since then (not shown). No other potential homolog pairs were identified.

As a crude measure of the likelihood of finding transposases on a given genome segment, the assembled genome length was divided by the number of annotated transposases and putative transposases annotated in IMG. Leaving aside the very incomplete *Cand*. Parabeggiatoa sp. SS genome, estimates range from one “transposase” every ~24,000 bp in *B. leptomitiformis* (178 in 4.3 mbp), through one every ~67–69,000 bp in *B. alba* and *T. ingrica*, to one every ~92,000 bp in BOGUAY (52 in 4.8 mbp). It is therefore not unusual to find one on the genome fragments illustrated in Figure 4, but neither can a contribution to transfer or rearrangement of the sensor proteins be ruled out. The annotation of toxin/antitoxin and restriction/methylation genes is more difficult because of the many classes of each (see e.g., MacGregor et al., 2013c, for BOGUAY), but they are not especially rare. It could be informative to investigate how many of these possible mobility functions are found in regions with phylogenetically mixed vs. homogeneous blastp affiliations.

By contrast to the wide phylogenetic range of blastp hits in the Beggiatoaceae genome segments, both of the Cyanobacterial predicted REC domain protein genes (Figures 4K,L) appear to be in much more stable regions. The *Microcoleus* sp. PCC 7113 one is upstream of three ORFs with predominately Cyanobacterial and Beggiatoaceae affiliations, then flanked by ORFs with only Cyanobacterial hits. The *Cylindrospermum stagnale* PCC 7417 one is flanked by ORFs with nearly or entirely Cyanobacterial affiliations. This suggests the Cyanobacteria as the immediate donor of these REC domain-containing gene segments to the Beggiatoaceae, rather than the other way around.

### Trees 19 and 9: paired REC domains

#### Inferred REC domain phylogeny

**Figure 5:**
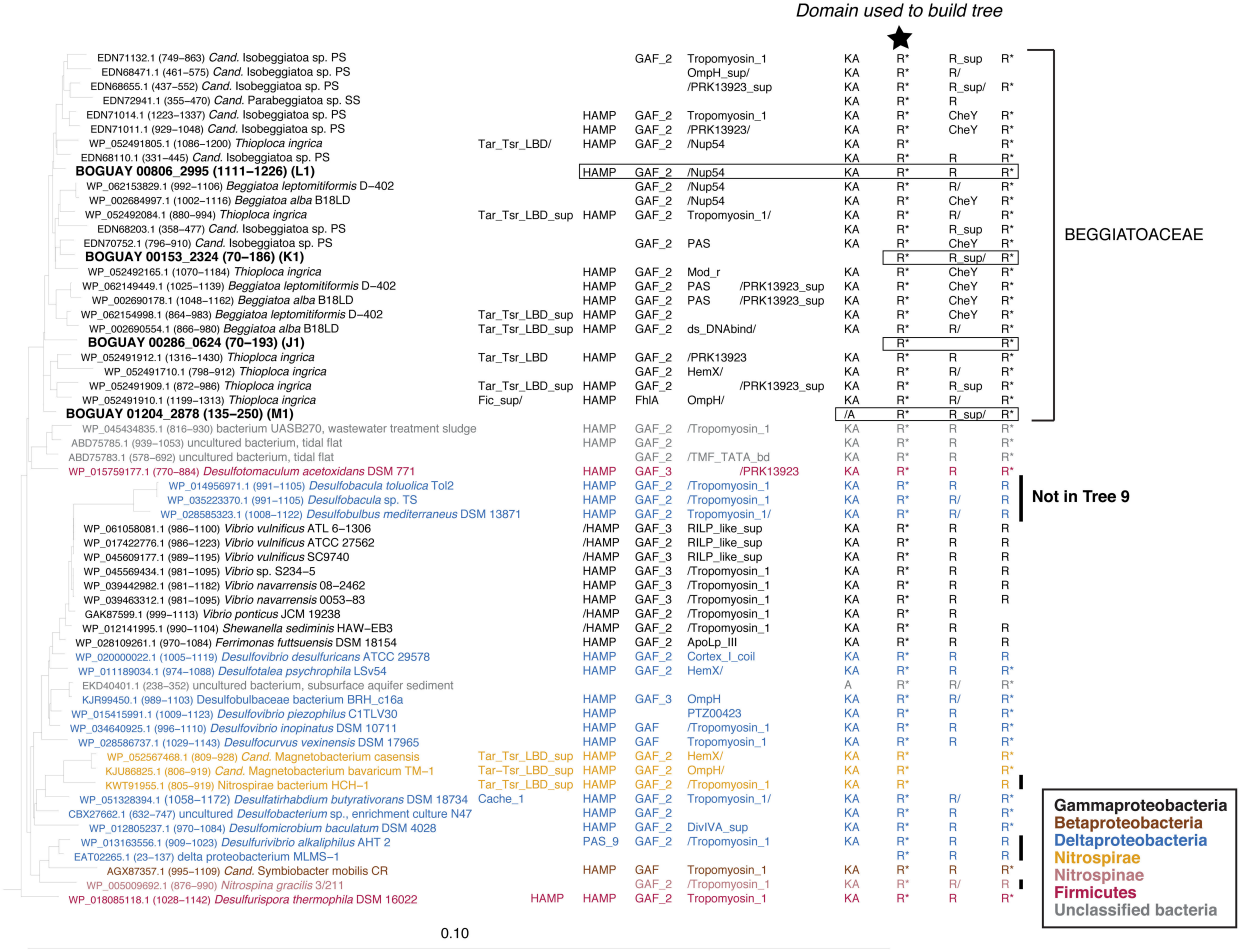
**REC domain Tree 19**. See legend to Figure 1 for methods. BOGUAY domain compositions are highlighted by boxes. Three *Paenibacillus* sequences were used to root the tree.

**Figure 6:**
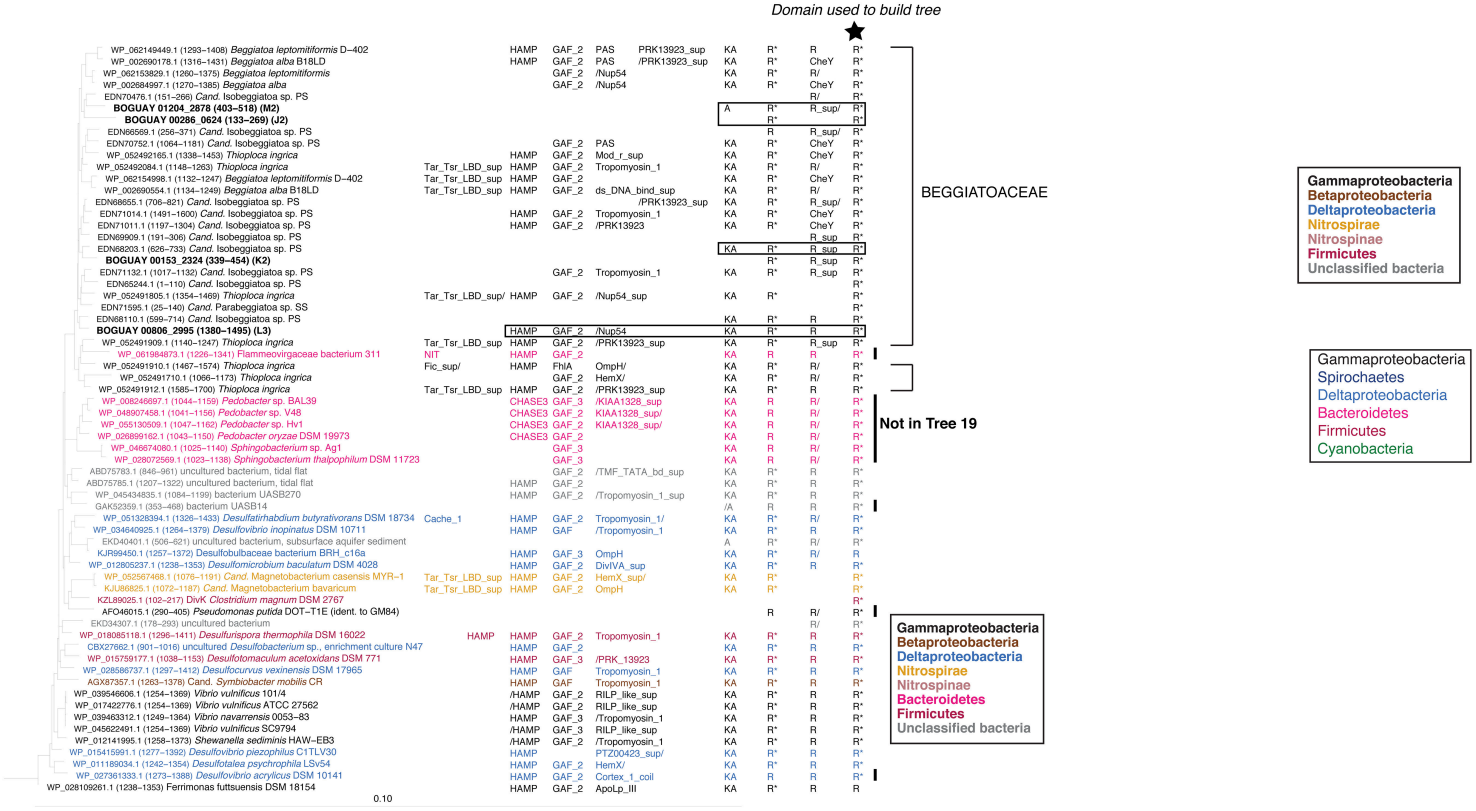
**REC domain Tree 9**. See legend to Figure 1 for methods. BOGUAY domain compositions are highlighted by boxes. Three *Paenibacillus* sequences were used to root the tree.

**Figure 7:**
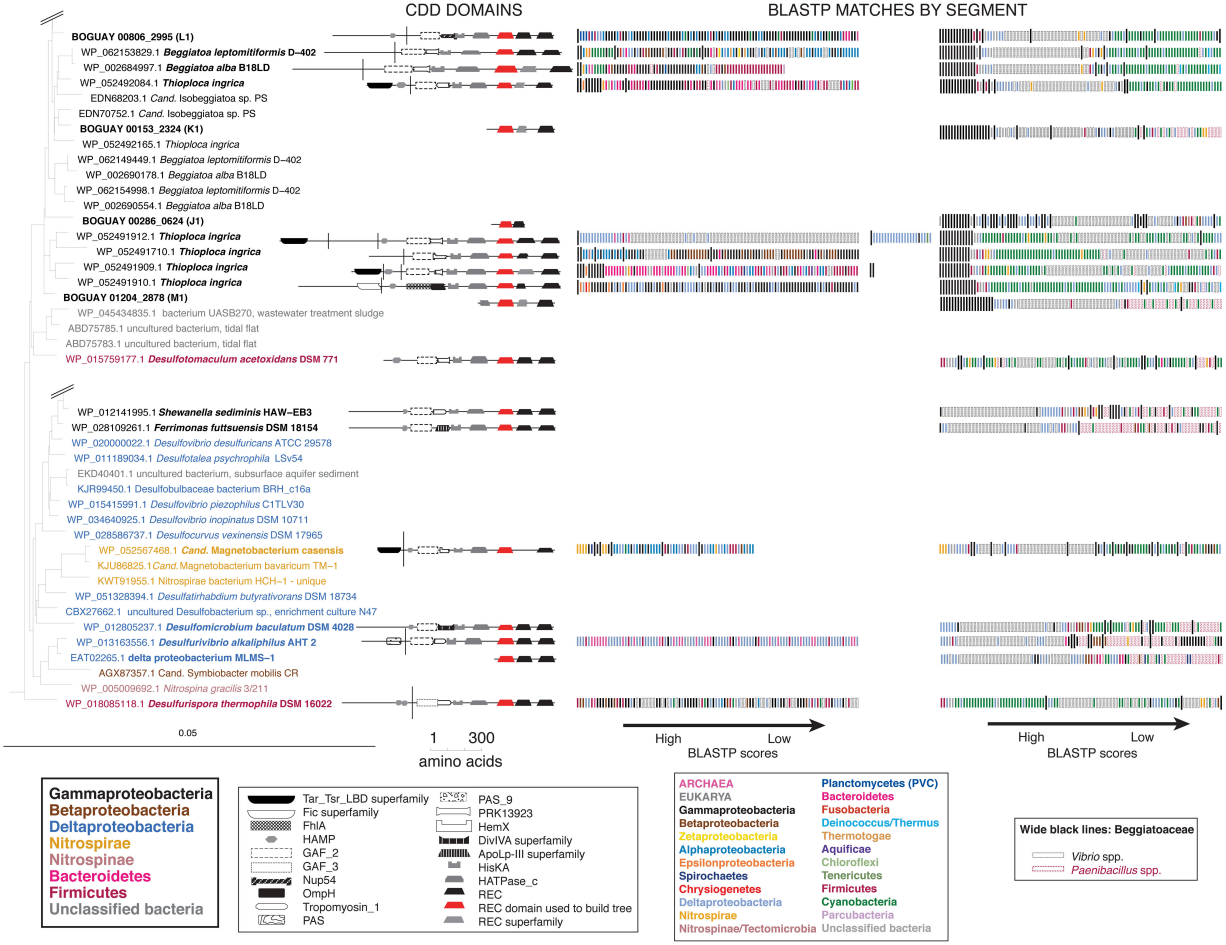
**Domain structure and color-coded BLASTP matches for selected Tree 19 sequences**. Vertical lines in domain cartoons indicate possible phylogenetic break points as identified by BLASTP, used to produce the color-coded diagrams. Open black (Gammaproteobacteria) and maroon (Firmicutes) bars were used for *Vibrio* and *Paenibacillus* spp., respectively, since these were dominant matches for the downstream segments.

The REC domains shown in Trees 19 and 9 (Figures 5, 6) are found as the first and last, respectively, of series of two or three REC domains at the C-terminal end of predicted proteins. In the Beggiatoaceae and most of the other species shown they occur together, and have similar inferred phylogenies. This suggests they may have undergone lateral transfer primarily as a unit, while upstream domains are more variable. The main exceptions are several predicted Deltaproteobacterial proteins found only in Tree 19, and a cluster of Sphingobacterales (Bacteroidetes; *Pedobacter* spp. in particular) found only in Tree 9. Because these are RAxML trees, identical sequences could not be included; *Pedobacter* is a well-studied genus with many published genomes, so the species shown are only a subset. Similarly, while the lists of *Vibrio* species do not completely overlap between the two trees, each one shown is only an example of a much larger group of primarily *Vibrio vulnificus* and *V. navarrensis* strains. For purposes of this paper it was not considered necessary to make a full concordance. An impression of the number of identical sequences can be gained from Figure 7 (open black bars).

#### BLASTP affiliations of complete proteins

For an overview of the affiliations of the complete predicted proteins, BLASTP searches (not shown) were used as above to identify possible boundaries in a subset of the Tree 19/Tree 9 sequences (Figure 7). First, this illustrates a problem with the method in its current state. Deeply sequenced genera such as *Vibrio* and *Paenibacillus* can dominate visually even if they represent only a small part of the phylogenetic range; they were assigned special colors to distinguish them from other Gammaproteobacteria and Firmicutes.

The downstream segment of this predicted protein group appears to have undergone duplication (or more) within the *Beggiatoaceae*, as evidenced by the clusters of wide black bands and similar banding patterns (e.g., compare BOGUAY “L1” and the *B. leptomitiformis, B. alba*, and *T. ingrica* predicted proteins grouped with it).

**Figure 8:**
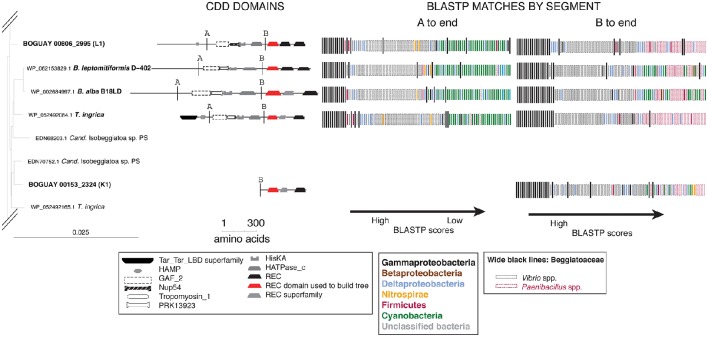
**BLASTP results for selected Tree 19 sequences truncated to match the shortest ORF**. Break points identified by BLASTP of the four longer sequences are indicated by line A. Break point B was placed halfway between the HATPase_c and first REC domains, to match the short sequence BOGUAY_2324 (K1).

Two of the predicted BOGUAY proteins (coded “K” and “L”), several of the *Isobeggiatoa* ones, and the *Parabeggiatoa* one begin at position 1 of their respective contigs, and therefore may be missing upstream domains. This can affect the BLASTP results, particularly for modular proteins. For example, BOGUAY 00153_2324 (“K”) appears distinct from the downstream segments of the other four predicted proteins shown for its REC domain clade (Figure 7, top right). However, when equivalent segments of each ORF are used, results for all five are similar (Figure 8, compare “A to end” and “B to end”). In particular, there are fewer Cyanobacterial and more *Paenibacillus* matches at the lower end of the scale, especially for the marine strains BOGUAY and *T. ingrica*.

#### Identification of possible transfer mechanisms

**Figure 9:**
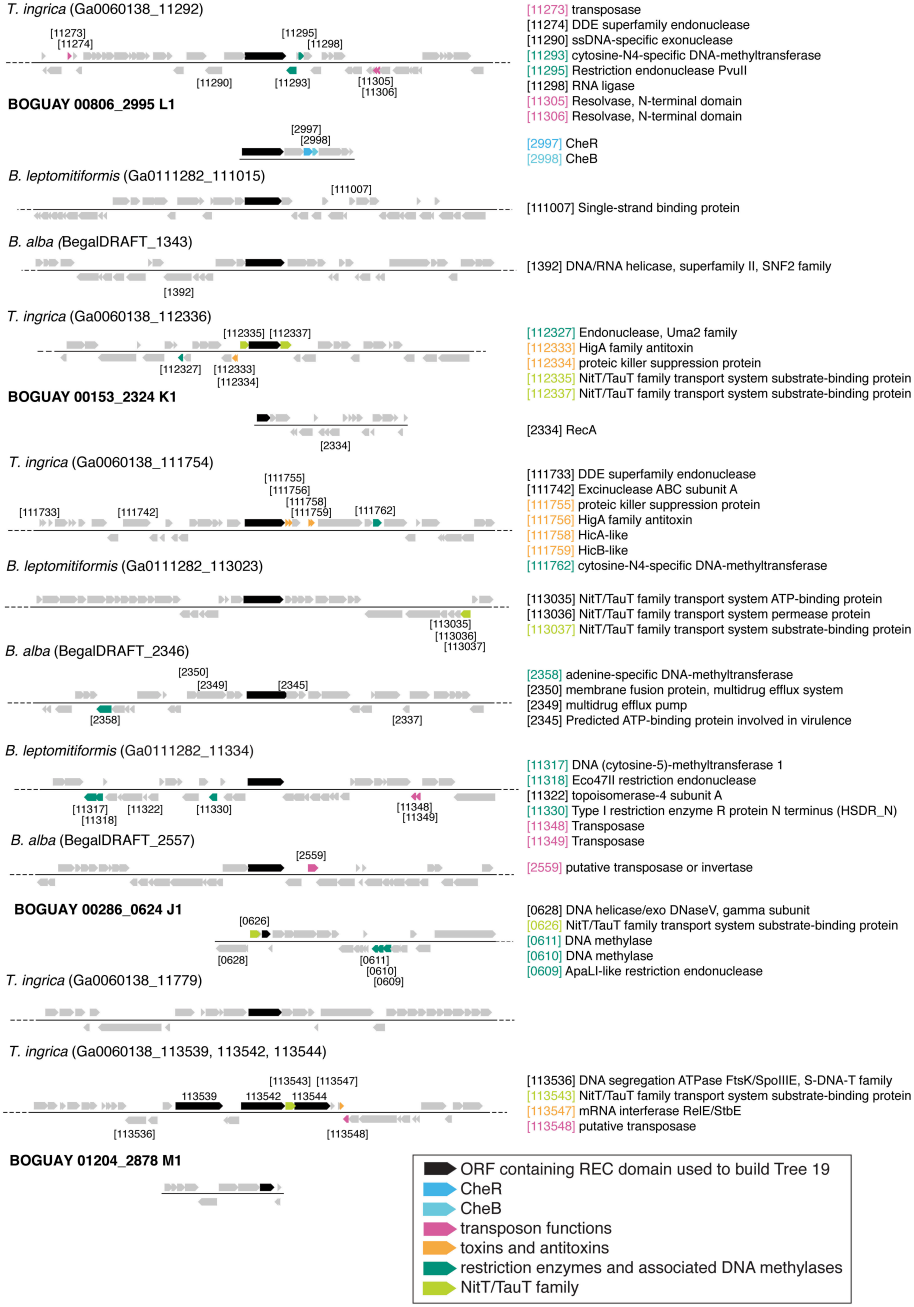
**Gene neighborhoods for Tree 19 and Tree 9 Beggiatoaceae**. Sequences are grouped by species.

**Figure 10:**
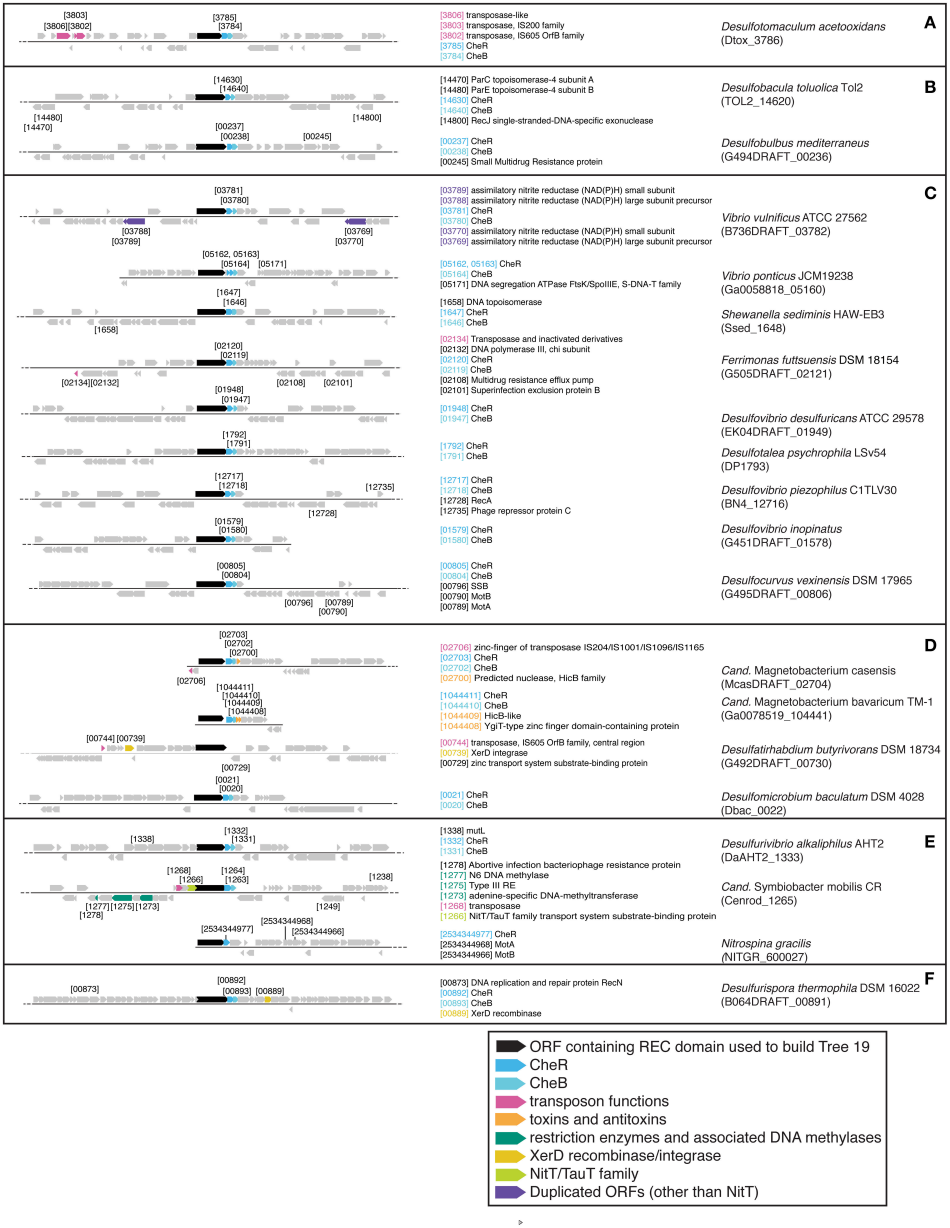
**Gene neighborhoods for Tree 19 non-Beggiatoaceae**. Sequences are grouped **(A–F)** according to clades in tree.

**Figure 11:**
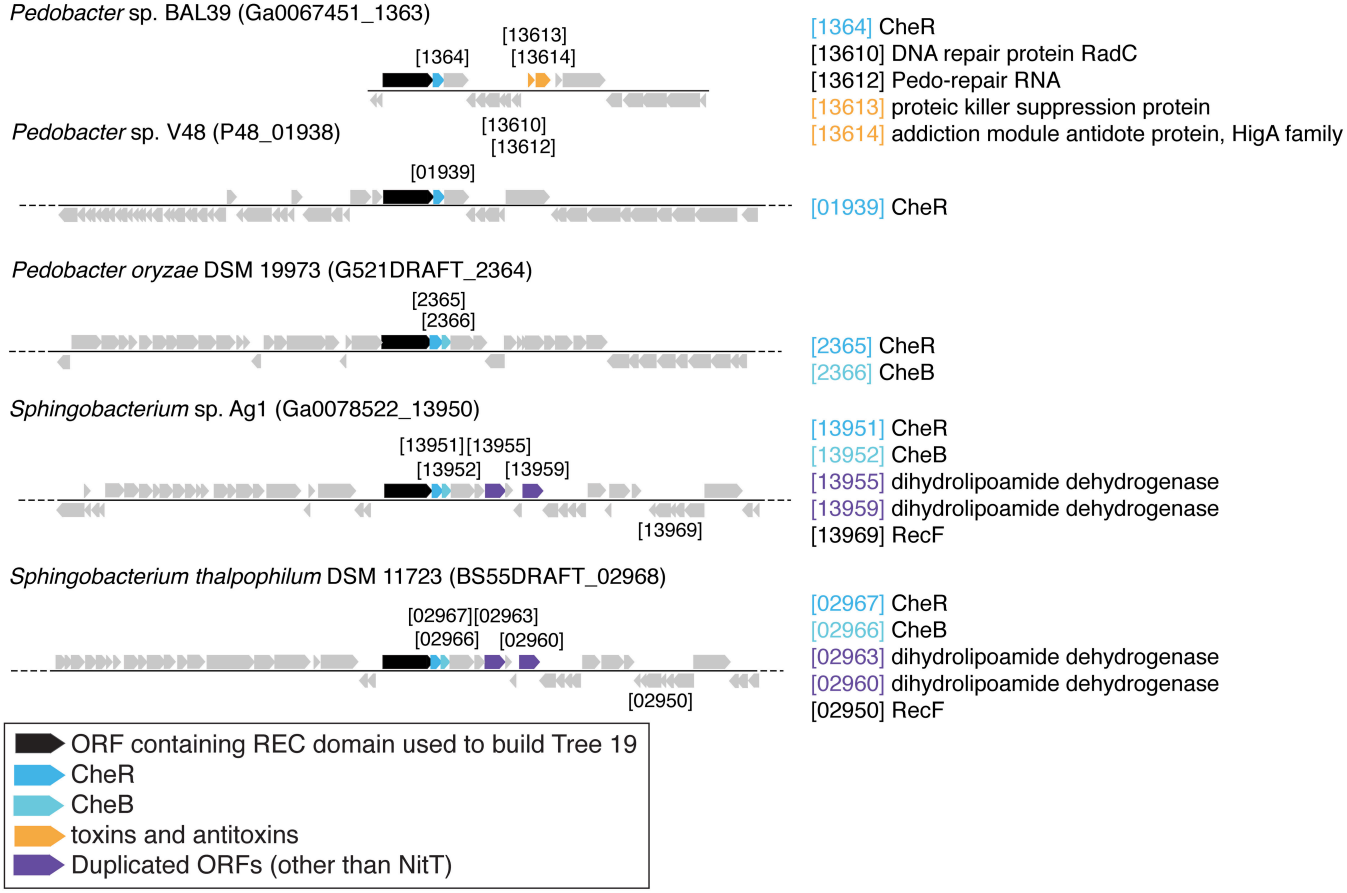
**Gene neighborhoods for Tree 9 Sphingobacteriaceae**.

A simpler look was taken at gene neighborhoods for these trees than for Tree 36. Possible indicators of genome rearrangements and horizontal gene transfer were highlighted in the immediate neighborhoods (arbitrarily defined as the 50 kb displayed by IMG) of the Tree 19 putative REC domain proteins (Figures 9–**11**). These include homologous ORFs, duplicated genes, transposons, toxin and antitoxin genes, XerD integrase/recombinase, and restriction enzyme and associated methylase genes. The predicted transposons, toxin/antitoxin, and restriction enzymes are sporadically distributed and no clearly related sets were found, nor do the two XerD-like sequences (Tree 10D, F) have any significant similarity (not shown). If any of these do have common ancestors, they have diverged or decayed considerably.

There are three examples of similar gene neighborhoods. First, the two *Sphingobacterium* regions from Tree 9 are very similar (**Figure 11**). Second, nearly all non-Beggiatoaceae have predicted genes for chemotaxis methyltransferase CheR/CheB directly downstream of the predicted REC domain protein gene; the exceptions are *Nitrospina gracilis* and two *Pedobacter* spp. (CheR only; Figures 10E, 11) and *Desulfatirhabdium butyrivorans* (neither; Figure 10D). By contrast, CheR/CheB are found in only one of the Beggiatoaceae neighborhoods (BOGUAY “L1”; Figure 9), where they are separated from the Tree 19/Tree 9 ORF BOGUAY_2995 by a second multi-REC domain signal transduction histidine kinase (BOGUAY_2996; CDD-predicted domains REC-HisKA-HATPase_c-REC/-REC/).

Third, NitT/TauT family transport system substrate-binding proteins are annotated directly upstream and downstream of one *T. ingrica* ORF (Figure 9, Ga0060138_112336); directly upstream of one BOGUAY (Figure 9, BOGUAY 00286_0624); between two of a set of three *T. ingrica* ORFs (Figure 9, Ga0060138_113542, 113544); directly upstream of the *Symbiobacterium* ORF (Figure 10E); and farther downstream of one *B. alba* ORF. This is suggestive of a pair of co-transferred genes, and a duplication event in *T. ingrica*. The upstream ORF of the *T. ingrica* pair is highly similar to the one from BOGUAY (*E*-value 8e-156, the closest current database match) and somewhat less so to the one from *Symbiobacter* (2e-15), suggesting these may be related by inheritance (within the Beggiatoaceae) or transfer. None of the remaining putative NitT/TauT sequences are significantly similar, however (not shown), including the two flanking the *T. ingrica* ORF; this REC domain protein may have taken on a role in more than one transport system.

There are also two examples of tandem repeats of highly similar ORFs that could record a recombination event: putative genes for large and small assimilatory nitrate reductase subunits in *V. vulnificus* ATCC 27562 (Figure 10C) and numerous other *V. vulnificus* strains (not shown), and two putative dihydrolipoamide dehydrogenase genes just downstream of the REC domain protein genes in the two *Sphingobacterium* strains shown (Figure 11) and several others (not shown).

## Discussion

In attempting to gain an overview of the sensor complement of the single-filament “Maribeggiatoa” Orange Guaymas (BOGUAY) genome, and its possible evolutionary origins, several obstacles were encountered. Their modular structure means that domains or domain clusters, rather than whole proteins, are the relevant evolutionary units in many cases. Ideally, phylogenetic reconstructions could be carried out for each individual domain or coherent group of domains, and the individual analyses recombined for visualization at different levels of detail.

From the work presented here, some of the desirable features of such a bioinformatic tool for modular proteins became clear. Many of the pieces are already available, but linkages are not seamless. In particular, the ability to reorder maps and diagrams with reference to user-generated phylogenetic trees (or, failing that, in user-selected order) would greatly ease the production of figures such as those shown.

The wish list also includes the following: (1) The data should remain easily updateable. (2) Segments defined by BLASTP scores (with default settings) may correctly segment some proteins, but well-defined domains are likely a better choice. It would be useful to be able to group or ungroup these; for example, HisKA-HATPase-REC seems to be a generally conserved unit. (3) Significance cutoffs should be adjustable, and large sets of near-identical sequences collapsible. (4) The level of phylogenetic resolution used for illustration should be customizable and flexible; in some cases phylum-level resolution is sufficient, in other cases—even in the same tree—species or even strain level may be needed (e.g., the many *V. vulnificus* strains). (5) Visual display of BLASTP results would be improved by including some representation of scores, perhaps as a bar graph parallel to the phylogenetically color-coded bars. (6) User-controlled color choices for phylogenetic and functional groups at different levels of specificity would aid understanding and presentation of results.

## Ethics statement

No human or animal subjects were involved in this study.

## Author contributions

The author confirms being the sole contributor of this work and approved it for publication.

## Funding

Genome sequencing was performed by the J. Craig Venter Institute, with funding from The Gordon and Betty Moore Foundation Marine Microbial Genome Sequencing Project. The use of RAST was supported in part by the National Institute of Allergy and Infectious Diseases, National Institutes of Health, Department of Health and Human Services (NIAD) under contract HHSN266200400042C. The Guaymas Basin project was funded by NSF OCE 0647633.

### Conflict of interest statement

The author declares that the research was conducted in the absence of any commercial or financial relationships that could be construed as a potential conflict of interest.
