# Supplementary material for: Visualizing Evolutionary Relationships of Multidomain Proteins: An Example from Receiver (REC) Domains of Sensor Histidine Kinases in the Candidatus Maribeggiatoa str. Orange Guaymas Draft Genome
Source: Front Microbiol. 2016 Nov 14;7:1780. doi: 10.3389/fmicb.2016.01780 (PMC5108060; doi:10.3389/fmicb.2016.01780)
Supplement: Supplementary file 1 [file Table1.PDF]

Supplemental Table 1. Protein domain abbreviations. Domain definitions are from the Conserved Domain Database (CDD) (1). Shorter abbreviations were used in figures for the three most common domains, shown at the top of the list.

**Domain abbreviation**	**Possible function (CDD)**
HisKA (K)	Histidine kinase; Dimerization and phosphoacceptor domain
HATPase_c (A)	Histidine kinase-like ATPases
REC (R)	Phosphoacceptor; contains a phosphoacceptor site that is phosphorylated by histidine kinase homologues
7TMR DISMED2	Extracellular receptor, possibly for carbohydrates
AAA	ATPase family associated with various cellular activities
ApoLp_III	Lipid transport (in insects)
Cache_1	Extracellular small-molecule recognition
CHASE, CHASE2, CHASE3	Extracellular ligand binding
CHD	Cyclase homology domains; includes eukaryotic and prokaryotic adenylate and guanylate cyclases
CheY	CheY-like receiver (REC) domain
COG3292	Predicted periplasmic ligand-binding sensor domain
COG3899	Predicted ATPase [General function prediction only]
COG4191	Signal transduction histidine kinase (C4-dicarboxylate transport)
Cortex_I_coil	18-heptad-repeat alpha-helical coiled-coil (known from an actin-bundling protein)
DivIVA_sup	Found as repeated domain in some proteins; known from a cell-division protein
ds_DNAbind	(not found)
EAL	Diguanylate phosphodiesterase. Named after conserved residues. Often found with GGDEF.
FhlA	Transcriptional regulator containing GAF, AAA-type ATPase, and DNA binding domains
Fic_sup	"Filamentation induced by cAMP"; known from cell-division protein
GAF, GAF_2, GAF_3	Sensor; Present in cGMP-specific phosphodiesterases, adenylyl and guanylyl cyclases, phytochromes, FhlA and NifA; may respond to cGMP, light, other proteins
GGDEF	c-di-GMP turnover. Shows homology to the adenylyl cyclase catalytic domain; found in variety of cell-signaling proteins. Often found with EAL.
HAMP	Signal transduction (by conformational change)
HemX	HemX is a membrane protein that downregulates HemA
MASE1	Predicted integral membrane sensory domain
Mod_r	"Modifier of rudimentary" (*Drosophila*); function unknown
Nup54	human Nup54 subunit of the nucleoporin complex

OmpH_sup	Outer membrane domain
PAC	Domain C-terminal to some PAS domains
PAS	Cytoplasmic sensor domain
PKc	Catalytic domain of protein kinases
PP2Cc	Serine/threonine phosphatases, family 2C, catalytic domain
PRK13923	Putative spore coat protein regulator protein in *B. subtilis*
PTZ00423	glideosome-associated protein 45 in apicomplexan parasites
Reg_prop	N-terminal domain of 14 tandem repeats , possibly in beta propeller conformation; DNA binding? Protein-protein interactions?
RILP_like_sup	Found in Rab interacting lysosomal protein-like 1 and 2 (Bilateria)
SpoIIE	Bacterial stage II sporulation E proteins: required for formation of a normal polar septum during sporulation. N-terminal region predicted membrane-spanning.
Tar_Tsr_LBD	Homodimeric receptors with N-terminal periplasmic ligand binding domain, transmembrane region, HAMP domain, and C-terminal cytosolic signaling domain
TMF_TATA_bd	C-terminal coiled coil of TATA element modulatory factor 1 proteins (eukaryotes), which compete with TATA-binding proteins
TPR	Tetratricopeptide repeat domain (uncertain function)
Tropomyosin_1	Eukaryotic morphogenesis
Vgb-SGL	Streptogramin lyase
Y_Y_Y	Mostly found at the end of the beta propellers (pfam07494) in a family of two component regulators.

References

1. **Marchler-Bauer A, Lu SN, Anderson JB, Chitsaz F, Derbyshire MK, DeWeese-Scott C, Fong JH, Geer LY, Geer RC, Gonzales NR, Gwadz M, Hurwitz DI, Jackson JD, Ke ZX, Lanczycki CJ, Lu F, Marchler GH, Mullokandov M, Omelchenko MV, Robertson CL, Song JS, Thanki N, Yamashita RA, Zhang DC, Zhang NG, Zheng CJ, Bryant SH.** 2011. CDD: a Conserved Domain Database for the functional annotation of proteins. Nucleic Acids Research **39:**D225-D229.
