# Supplementary material for: Visualizing Evolutionary Relationships of Multidomain Proteins: An Example from Receiver (REC) Domains of Sensor Histidine Kinases in the Candidatus Maribeggiatoa str. Orange Guaymas Draft Genome
Source: Front Microbiol. 2016 Nov 14;7:1780. doi: 10.3389/fmicb.2016.01780 (PMC5108060; doi:10.3389/fmicb.2016.01780)
Supplement: Supplementary file 2 [file Table2.PDF]

Supplemental Table 2. Tree 36 ORF descriptions. See Fig. 5.

(A) *Beggiatoa alba* B18LD

BegalDRAFT_1099	thiamine-phosphate diphosphorylase
BegalDRAFT_1098	sulfur carrier protein ThiS
BegalDRAFT_1097	thiazole-phosphate synthase
BegalDRAFT_1096	Co^{2+}/Ni^{2+} permease
BegalDRAFT_1095	Ni^{2+} transport protein
BegalDRAFT_1094	Co^{2+}/Ni^{2+} transport protein
BegalDRAFT_1093	trans-aconitate 2-methyltransferase
**BegalDRAFT_1092**	**PAS domain S-box and diguanylate cyclase (GGDEF) domain-containing protein**
BegalDRAFT_1091	transcriptional regulator, TetR family
BegalDRAFT_1090	3-isopropylmalate/(R)-2-methylmalate dehydratase, large subunit

(B) Orange Guaymas "Maribeggiatoa"

BOGUAY_2059	ornithine carbamoyltransferase
**BOGUAY_2058**	**signal transduction histidine kinase**
BOGUAY_2057	phosphate-selective porin OprO and OprP
BOGUAY_2056	tRNA-2-methylthio-N6-dimethylallyladenosine synthase

(C) "Isobeggiatoa" PS

**BGP_5448**	**response regulator receiver protein**
BGP_5449	periplasmic sensor signal transduction histidine kinase
BGP_5450	hypothetical protein

(D) Thioploca ingrica

Ga0060138_111087	glucose-6-phosphate 1-epimerase
Ga0060138_111086	glucose-6-phosphate 1-dehydrogenase
Ga0060138_111085	endonuclease, Uma2 family
Ga0060138_111084	NYN domain protein
Ga0060138_111083	methyltransferase domain protein
Ga0060138_111082	tetratricopeptide repeat
Ga0060138_111081	OmpA family protein
Ga0060138_111080	hypothetical protein
**Ga0060138_111079**	**PAS domain S-box-containing protein**
Ga0060138_111078	signal transduction histidine kinase
Ga0060138_111077	RNA polymerase, sigma 32 subunit, RpoH
Ga0060138_111076	hypothetical protein
Ga0060138_111075	hypothetical protein
Ga0060138_111074	sulfate permease, SulP family

(E) "Isobeggiatoa" PS

BGP_0360	hypothetical protein
**BGP_0359**	**adenylate cyclase**

(F) Orange Guaymas "Maribeggiatoa"

BOGUAY_2962	hypothetical protein
**BOGUAY_2961**	**diguanylate cyclase (GGDEF) domain-containing protein**
BOGUAY_2960	toxin?
BOGUAY_2959	exonuclease
BOGUAY_2958	antitoxin?
BOGUAY_2957	Type I restriction enzyme R protein N terminus (BOGUAY_0693-like)
BOGUAY_2956	(no matches)
BOGUAY_2955	Penicillin-binding protein 4

(G) Orange Guaymas "Maribeggiatoa"

BOGUAY_1296	general secretion pathway protein E
**BOGUAY_1295**	**PAS domain S-box-containing protein**
BOGUAY_1294	dUTP pyrophosphatase
BOGUAY_1293	hypothetical protein (no matches)
BOGUAY_1292	Signal transduction histidine kinase
BOGUAY_1291	Signal transduction histidine kinase
BOGUAY_1290	dethiobiotin synthetase

(H) Orange Guaymas "Maribeggiatoa"

BOGUAY_1125	filamentous hemagglutinin family N-terminal domain-containing protein
BOGUAY_1126	PD-(D/E)XK nuclease superfamily protein
BOGUAY_1127	protein of unknown function (DUF928)
[BOGUAY_1128]	[CHAT domain-containing protein]
BOGUAY_1129	hypothetical protein
BOGUAY_1130	Regulator of chromosome condensation (RCC1) repeat-containing protein
BOGUAY_1131	hypothetical protein
BOGUAY_1132	MazF
BOGUAY_1133	hypothetical protein
BOGUAY_1134	chloride channel protein, ClC family
BOGUAY_1135	conserved hypothetical protein
BOGUAY_1136	N-6 DNA Methylase
BOGUAY_1137	conserved hypothetical protein
BOGUAY_1138	Predicted Fe-Mo cluster-binding protein, NifX family
BOGUAY_1139	NMT1/THI5 like (putative lipoprotein)
BOGUAY_1140	hypothetical protein
BOGUAY_1141	hypothetical protein
BOGUAY_1142	HAMP domain-containing protein
BOGUAY_1143	conserved domain protein
{BOGUAY_1144}	{hypothetical protein}
**BOGUAY_1145**	**Serine phosphatase RsbU, regulator of sigma subunit**
BOGUAY_1146	Uncharacterized iron-regulated protein
BOGUAY_1147	AAA-like domain-containing protein
BOGUAY_1148	fumarase, class I, homodimeric
BOGUAY_1149	hypothetical protein
BOGUAY_1150	hypothetical protein
BOGUAY_1151	ribonucleoside-diphosphate reductase class II

Transposon functions
Toxins and antitoxins
Restriction enzymes and associated DNA methylases

[], {} homologous ORFs in different species

(I) *Thioploca ingrica*

Ga0060138_111822	NAD(P)H-hydrate epimerase
Ga0060138_111821	tRNA threonylcarbamoyladenosine biosynthesis protein TsaE
Ga0060138_111820	UDP-sulfoquinovose synthase
Ga0060138_111819	hypothetical protein
Ga0060138_111818	Peroxiredoxin
Ga0060138_111817	Transglycosylase SLT domain-containing protein
Ga0060138_111816	hypothetical protein
Ga0060138_111815	hypothetical protein
Ga0060138_111814	hypothetical protein
Ga0060138_111813	hypothetical protein (no significant matches)
Ga0060138_111812	hypothetical protein
Ga0060138_111811	Transposase
Ga0060138_111810	Tellurite resistance protein
Ga0060138_111809	hypothetical protein
Ga0060138_111808	hydrogenase expression/formation protein HypD
Ga0060138_111807	hypothetical protein
Ga0060138_111806	Predicted PurR-regulated permease PerM
Ga0060138_111805	Ankyrin repeat-containing protein
Ga0060138_111804	hypothetical protein
[Ga0060138_111803]	[CHAT domain-containing protein]
{Ga0060138_111802}	{hypothetical protein}
**Ga0060138_111801**	**Serine phosphatase RsbU, regulator of sigma subunit**
Ga0060138_111800	hypothetical protein
Ga0060138_111799	hypothetical protein
Ga0060138_111798	protein of unknown function DUF29
Ga0060138_111797	Phospholipid N-methyltransferase
Ga0060138_111796	NADPH-dependent glutamate synthase beta chain
Ga0060138_111795	YgiT-type zinc finger domain-containing protein
Ga0060138_111794	pyruvate ferredoxin oxidoreductase beta subunit
Ga0060138_111793	pyruvate ferredoxin oxidoreductase alpha subunit
Ga0060138_111792	pyruvate ferredoxin oxidoreductase gamma subunit
Ga0060138_1117991	hypothetical protein
Ga0060138_111790	hypothetical protein
Ga0060138_111789	Listeria/Bacterioides repeat-containing protein
Ga0060138_111788	ribosomal-protein-alanine N-acetyltransferase
Ga0060138_111787	hypothetical protein
Ga0060138_111786	anthranilate synthase component 1
Ga0060138_111785	phosphoglycolate phosphatase

(J) *Cand.* Thiomargarita nelsonii

**Ga0097846_104775**	**PAS domain S-box-containing protein**
Ga0097846_104774	release factor glutamine methyltransferase
Ga0097846_104773	hypothetical protein
Ga0097846_104772	hypothetical protein

(K) *Microcoleus* sp. PCC 7113

Mic7113_0731	4-amino-4-deoxychorismate lyase
Mic7113_0732	hypothetical protein
Mic7113_0733	Serine phosphatase RsbU, regulator of sigma subunit
Mic7113_0734	hypothetical protein
Mic7113_0735	hypothetical protein
**Mic7113_0736**	**PAS domain S-box-containing protein**
Mic7113_0737	large subunit ribosomal protein L35

(L) *Cylindrospermum stagnale* PCC 7417

CylstDRAFT_4280	Tetratricopeptide repeat-containing protein
CylstDRAFT_4281	sulfate transport system substrate-binding protein
**CylstDRAFT_4282**	**Signal transduction histidine kinase**
CylstDRAFT_4283	hypothetical protein
CylstDRAFT_4284	Uncharacterized membrane protein, DUF373 family
