# Supplementary material for: Visualizing Evolutionary Relationships of Multidomain Proteins: An Example from Receiver (REC) Domains of Sensor Histidine Kinases in the Candidatus Maribeggiatoa str. Orange Guaymas Draft Genome
Source: Front Microbiol. 2016 Nov 14;7:1780. doi: 10.3389/fmicb.2016.01780 (PMC5108060; doi:10.3389/fmicb.2016.01780)
Supplement: Supplementary file 6 [file DataSheet4.PDF]

```
ID   ENBZZZZ4   ami; 110 BP.
XX
AC   ARB_436BE9C8;
OS   EDN71014.1 (1491-1600) Beggiatoa sp. PS
XX
XX
XX
XX
XX
XX
FT   source          1..110
XX
SQ   Sequence 110 BP;
1         LIVDDDPRNT FALATILEDK --NMEVIAGE NGHEALELLE QHEDIAIVLM
51        DIMMPEMDGY EAMRQIRAQK NRFRRQLPII ALTAKAMKGD KTKCIEAGAN
101       DYLSKPVDTD KL........
//
ID   ENBZZZZ7   ami; 108 BP.
XX
AC   ARB_E8FD1FE9;
OS   EDN71011.1 (1197-1304) Beggiatoa sp. PS
XX
XX
XX
XX
XX
XX
FT   source          1..108
XX
SQ   Sequence 108 BP;
1         LIVDDDARNT FALAIVLEEK --QMEVIAGN NGFEALKLLD EHQDIAIVLM
51        DIMMPEMDGY EAMRQIRAR- DGFR-KLPII ALTAKAMKSD KAKCIEAGAN
101       DYLSKPVDTD KL........
//
ID   ASJZZZZ7   ami; 108 BP.
XX
AC   ARB_E7AED6EA;
OS   WP_052491909.1 (1140-1247) Thioploca ingrica
XX
XX
XX
XX
XX
XX
FT   source          1..108
XX
SQ   Sequence 108 BP;
1         LIVDDDVRNV FALAATLEDK --GMEIVVAH NGQEALPILE TQTDISVILM
51        DIMMPGMDGY ETIRQIRSQ- VRFR-KLPII ALTAKAMKGD KAKCIEAGAS
101       DYLAKPFDAN KL........
```

```
//
ID   ASOZZZZ2   ami; 113 BP.
XX
AC   ARB_3BC43F52;
OS   WP_011189034.1 (1242-1354) Desulfotalea psychrophila LSv54
XX
XX
XX
XX
XX
XX
FT   source          1..113
XX
SQ   Sequence 113 BP;
1         LLVDDDMRNV FALSSVLEER --GINIVVAR DGIECLEKLK EQDHFDAVLM
51        DIMMPRMDGY EAMQEIRKN- IQHK-KLPII ALTAKAMKGD RSKCIEAGAS
101       DYLAKPVDAD KLISMLR...
//
ID   ASJZZZZ9   ami; 108 BP.
XX
AC   ARB_FC1EBA88;
OS   WP_052491910.1 (1467-1574) Thioploca ingrica
XX
XX
XX
XX
XX
XX
FT   source          1..108
XX
SQ   Sequence 108 BP;
1         LIVDDDIRNV FALTATLEDK --NMEIVAAH NGLQALQALE NHPDVAVVLM
51        DIMMPEMDGY EATRKIRAQ- IRFR-KLPII ALTAKAMRDD KAKCIAAGAN
101       DYLSKPVDGE RL........
//
ID   ENBZZZ12   ami; 108 BP.
XX
AC   ARB_BE4FB2B4;
OS   EDN68203.1 (626-733) Beggiatoa sp. PS (dup)
XX
XX
XX
XX
XX
XX
FT   source          1..108
XX
SQ   Sequence 108 BP;
1         LIVDDDVRNT YALATVLEDR --EMEVVVGN NGNQALELLE KHSDIAIVLM
51        DVMMPKMDGY EAIHQIRAQ- PRYR-HLPII ALTAKAMKGD KAKCIEAGAN
```

```
101       DYLSKPVDTE KL........
//
ID   AT6ZZZZ2   ami; 108 BP.
XX
AC   ARB_421BA4AC;
OS   WP_051328394.1 (1326-1433) Desulfatirhabdium butyrativorans DSM
18734
XX
XX
XX
XX
XX
XX
FT   source          1..108
XX
SQ   Sequence 108 BP;
1         LLVDDDMRNV YAITNILEEK --GMRVVVGK NGKEGIERLK ADPGIHLVLM
51        DIMMPVMDGY EAMRRIRAM- DVYK-KLPII ALTAKAMKGD KALCIEAGAN
101       DYLAKPFDTD KL........
//
ID   ASJZZZ12   ami; 108 BP.
XX
AC   ARB_7892ACDB;
OS   WP_052491710.1 (1066-1173) Thioploca ingrica
XX
XX
XX
XX
XX
XX
FT   source          1..108
XX
SQ   Sequence 108 BP;
1         LLVDDDMRNV FALAADLEKE --GIETLVAH DGHSAIQQLQ AKPDIAMVLM
51        DMMMPEIDGY NAIRQIRAQ- PQFR-QLPII ALIAKAMKAD RTKCLEAGAN
101       DYLAKPINTH NL........
//
ID   ATKZZZZ2   ami; 108 BP.
XX
AC   ARB_6B094892;
OS   WP_026899162.1 (1043-1150) Pedobacter oryzae DSM 19973
XX
XX
XX
XX
XX
XX
FT   source          1..108
XX
SQ   Sequence 108 BP;
```

```
1         LIVDDDMRNI FALSSALQDY --NMQIEIAN NGLEALQKLD ENPDINLVLM
51        DIMMPEMDGY EAMQEIRKQ- SRFA-KLPLM ALTAKAMKND REKCIEAGAN
101       DYISKPVDIN KL........
//
ID   ResreYy3   ami; 116 BP.
XX
AC   ARB_3566A8C5;
OS   BOGUAY 00153_2324 (339-454) K2
XX
XX
XX
XX
XX
XX
FT   source          1..116
XX
SQ   Sequence 116 BP;
1         LIVDDDVRNT FALATVLEDH --DMEVVTGS DGKEGLAVLE QNDDVAIVLM
51        DIMMPEMDGY EAIRKIREQ- PRYR-HFPII ALTAKAMKGD KTKCIKAGAN
101       DYLAKPVDTD KLLSLMRVWL
//
ID   BgJZZZZZ   ami; 116 BP.
XX
AC   ARB_EC5B975B;
OS   BOGUAY 00286_0624 (133-269) J2
XX
XX
XX
XX
XX
XX
FT   source          1..116
XX
SQ   Sequence 116 BP;
1         EAKKMGAIGY LIKPVSVDEK --EMEVLVAD NGKEALKKLE KEPNIDIVLM
51        DIMMPEMDGY KAIQEIRTQ- PRFR-QLPII ALTAKAMKGD KAKCIEAGAN
101       DYLAKPMDTD KLFSLMRVWL
//
ID   ResreY11   ami; 116 BP.
XX
AC   ARB_233E72A4;
OS   BOGUAY 01204_2878 (403-518) M2
XX
XX
XX
XX
XX
XX
FT   source          1..116
XX
```

```
SQ   Sequence 116 BP;
1         LIVDDDMRNS FALTTVLEGK --EMVVTVAE TGTEALQKLE QHKDVAIILM
51        DIMMPEMDGY EAILKIKEQ- PQYQ-KLPII ALTAKAMKGD KTKCLEAGAS
101       DYLAKPVDTE KLISLMRVWL
//
ID   RsrsYyy5   ami; 116 BP.
XX
AC   ARB_ABF1EFB6;
OS   BOGUAY 00806_2995 (1380-1495) L3
XX
XX
XX
XX
XX
XX
FT   source          1..116
XX
SQ   Sequence 116 BP;
1         LIVDDDVRNV FALATVLEDK --DMEVVVAK DGEKALELLG EHEEIAIVLM
51        DIMMPGMDGY EAMRKIRIQ- PRYR-KLPII ALTAKAMKGD KAKCIEAGAN
101       DYLSKPMDTH KLLSLMRVWL
//
ID   ENgZZZZ2   ami; 116 BP.
XX
AC   ARB_BDD69F21;
OS   EDN71595.1 (25-140) Beggiatoa sp. SS
XX
XX
XX
XX
XX
XX
FT   source          1..116
XX
SQ   Sequence 116 BP;
1         LIVDDDVRNT YALATVLEEK --EMEVIGAK NGIEALAILK EHENIALILM
51        DIMMPEMDGY EAMRQIRKQ- PKHR-QLPII ALTAKAMKGD KAKCIEAGAN
101       DYLSKPVDTD KLISLMRVWL
//
ID   ENBZZZ13   ami; 116 BP.
XX
AC   ARB_84EDFC87;
OS   EDN69909.1 (191-306) Beggiatoa sp. PS
XX
XX
XX
XX
XX
XX
FT   source          1..116
```

```
XX
SQ   Sequence 116 BP;
1         LIVDDDARNI FALAIVLEDK --NMEVIAGN HGKEALTLLD EHPDIAIILM
51        DIMMPEMDGY EAIRKIREQ- PRFK-RLPII ALTAKAMKGD KAKCIEAGAN
101       DYLSKPVNTD KLISLMRVWL
//
ID   ENBZZZ15   ami; 116 BP.
XX
AC   ARB_E902D9A5;
OS   EDN66569.1 (256-371) Beggiatoa sp. PS (dup)
XX
XX
XX
XX
XX
XX
FT   source          1..116
XX
SQ   Sequence 116 BP;
1         LVVDDDMRNA FALVTVLEDH --DMEVFCGK NGKKALAVLD EHPDIAIVLM
51        DIMMPEMDGY EAMCEIRKQ- SRFY-QLPII ALTAKAMKGD KAKCIEAGAS
101       DYLSKPVDTD KLLSLMRVWL
//
ID   ENBZZZ19   ami; 116 BP.
XX
AC   ARB_B2741AE2;
OS   EDN70476.1 (151-266) Beggiatoa sp. PS
XX
XX
XX
XX
XX
XX
FT   source          1..116
XX
SQ   Sequence 116 BP;
1         LLVDDDVRNT FALMTFLESK --NMEVIVAE NGKEALAALE EHPNIDIVLM
51        DIMMPEMDGY EAIHEIREQ- PRFH-KLPII AITAKAMKGD KVKCIEAGAS
101       DYLAKPVDTD KLVSLIRVWL
//
ID   ENBZZZ20   ami; 116 BP.
XX
AC   ARB_D1A3539E;
OS   EDN68110.1 (599-714) Beggiatoa sp. PS (dup)
XX
XX
XX
XX
XX
XX
```

```
FT   source          1..116
XX
SQ   Sequence 116 BP;
1          LIVDDDSRNV FALATVLENY --DMEVIFAD NGKKALKLLE EQDNIAIVLM
51         DIMMPEMDGY EAMRKIRAQ- ARFR-QLPII ALTAKAMKGD KAKCIEAGAN
101        DYLAKPVDTD KLISLMRVWL
//
ID   ASJZZZ13   ami; 116 BP.
XX
AC   ARB_413A0C9A;
OS   WP_052492084.1 (1148-1263) Thioploca ingrica (dup)
XX
XX
XX
XX
XX
XX
FT   source          1..116
XX
SQ   Sequence 116 BP;
1          LIVDDDIRNT FALATVLEDH --DVEVKVAQ NGKEALTVLE EHPEIDIVLM
51         DIMMPEMDGY EAMQKIRAQ- PRFR-KLPII ALTAKAMKGD RAKCIEAGAN
101        DYLSKPVDTD KLISLMRVWL
//
ID   ENBZZZ23   ami; 116 BP.
XX
AC   ARB_A652DD01;
OS   EDN71132.1 (1017-1132) Beggiatoa sp. PS (dup)
XX
XX
XX
XX
XX
XX
FT   source          1..116
XX
SQ   Sequence 116 BP;
1          LIVDDDMRNI FALATVLEDK --NMEVVSAD NGHEALKLLA ENSDIALVLM
51         DIMMPEMDGY ETMRQIRQS- PNFR-KLPII ALTAKAMKGD KAKCIEAGAN
101        DYLSKPVDTD KLISLMRVWL
//
ID   ENBZZZ28   ami; 116 BP.
XX
AC   ARB_6FC27AFB;
OS   EDN68655.1 (706-821) Beggiatoa sp. PS
XX
XX
XX
XX
XX
```

```
XX
FT   source          1..116
XX
SQ   Sequence 116 BP;
1         LIVDDDPRNT FALATVLEDK --QMEVIAGN NGNEALELLD EHQNITIVLM
51        DIMMPEMDGY EAMRQIRAQ- DRFR-KLPII ALTAKAMKGD KSKCIEAGAN
101       DYLSKPVDTD KLISLMRVWL
//
ID   AVMZZZZZ   ami; 116 BP.
XX
AC   ARB_F50B9793;
OS   WP_062153829.1 (1260-1375) Beggiatoa leptomitiformis (dup)
XX
XX
XX
XX
XX
XX
FT   source          1..116
XX
SQ   Sequence 116 BP;
1         LIVDDDVRNT FALTTVLENK --NMQVLVSE NGKEGLDMLS LNPDISMVLM
51        DVMMPEMDGY EAMRAIRAQ- LRFR-KLPII ALTAKAMKGD KAKCIEAGAN
101       DYLTKPVDTD KLLSLMRVWL
//
ID   ENBZZZ29   ami; 110 BP.
XX
AC   ARB_A4C2F450;
OS   EDN65244.1 (1-110) Beggiatoa sp. PS
XX
XX
XX
XX
XX
XX
FT   source          1..110
XX
SQ   Sequence 110 BP;
1         ------MRNI FSLASVLEDH --EMEIIIAN NGNEALKKLE AHQGIALVLM
51        DIMMPEKDGY ETMREIRTQ- PNYR-KLPII ALTAKAMKDD KAKCIEAGAN
101       DYLSKPVDTE KLLSLIRVWL
//
ID   AVOZZZZZ   ami; 116 BP.
XX
AC   ARB_270B49ED;
OS   WP_002684997.1 (1270-1385) Beggiatoa alba (dup)
XX
XX
XX
XX
```

```
XX
XX
FT   source          1..116
XX
SQ   Sequence 116 BP;
1          LIVDDDVRNT FALTTVLENK --NMQVLVSE NGKEALNLLN ENPDISMILM
51         DVMMPEMDGY EAMRAIRSQ- LRFR-KLPII ALTAKAMKGD KAKCIEAGAN
101        DYLTKPVDTD KLLSLMRVWL
//
ID   WtDsYyyy   ami; 116 BP.
XX
AC   ARB_E68C802A;
OS   WP_027361333.1 (1273-1388) Desulfovibrio acrylicus DSM 10141 (see
D. desulfuricans)(dup)
XX
XX
XX
XX
XX
XX
FT   source          1..116
XX
SQ   Sequence 116 BP;
1          MIVDDDMRNV FALSSVLEDR --NMDVVVAK NGIECLEKLE ELDSIDCVLM
51         DIMMPQMDGY EAMTEIRKN- PEYA-KLPII ALTAKAMKGD RSKCIDCGAS
101        DYLSKPVNTE KLISMMRVWL
//
ID   ASJZZZ16   ami; 116 BP.
XX
AC   ARB_1D440676;
OS   WP_052492165.1 (1338-1453) Thioploca ingrica
XX
XX
XX
XX
XX
XX
FT   source          1..116
XX
SQ   Sequence 116 BP;
1          LLVDDDMRNI FALVSVLEDR --DMKVFIAQ NGKEALNLLE QHPDIDIVLM
51         DIMMPEMDGY EAMRKIRSQ- TRFS-KLPII ALTAKAMKGD KIKCIDAGAN
101        DYIAKPIDTD KLISLMKVWL
//
ID   AVXZZZZZ   ami; 116 BP.
XX
AC   ARB_5413A8B3;
OS   WP_028109261.1 (1238-1353) Ferrimonas futtsuensis DSM 18154
XX
XX
```

```
XX
XX
XX
XX
FT   source          1..116
XX
SQ   Sequence 116 BP;
1         LLVDDDMRNV FALSSVLEEK --GLAVTIAR NGFEALEKLE EMEQVDGVLM
51        DIMMPKMDGY EAMRQIRQR- SDWA-KLPII ALTAKAMKGD RSKCIEAGAS
101       DYLAKPVDTD KLLSMLRVWL
//
ID   ABDpuYy2   ami; 116 BP.
XX
AC   ARB_9378D8E0;
OS   ABD75785.1 (1207-1322) uncultured bacterium, tidal flat
XX
XX
XX
XX
XX
XX
FT   source          1..116
XX
SQ   Sequence 116 BP;
1         LMVDDDMRNV FALSSILEEK --GMQVLIGE HGQDAMELLD QNPDIDLVLM
51        DIMMPEMDGY EAMRQIRQQ- PKFK-TLPII ALTAKAMKGD RQKCIDAGAN
101       DYLSKPVDTD KLLSLLRVWL
//
ID   WtfrYyyy   ami; 116 BP.
XX
AC   ARB_95CBA7DA;
OS   WP_018085118.1 (1296-1411) Desulfurispora thermophila DSM 16022
XX
XX
XX
XX
XX
XX
FT   source          1..116
XX
SQ   Sequence 116 BP;
1         LLADDDMRNV FALLSILEEK --GMEILVAR NGREALQKLA EHPDTDLVLM
51        DIMMPEMDGY QAMREIRKQ- ERFK-YLPII ALTAKAMKGD REKSIAAGAS
101       DYLSKPVDPD KLLSLLRVWL
//
ID   AW3ZZZZZ   ami; 116 BP.
XX
AC   ARB_6A88131B;
OS   WP_055130509.1 (1047-1162) Pedobacter sp. Hv1
XX
```

```
XX
XX
XX
XX
XX
FT   source          1..116
XX
SQ   Sequence 116 BP;
1         LITDDDMRNI FALSSALQAY --DIKIIIAN NGIEALAKIE ENPQIDLVLM
51        DIMMPEMDGY EAMRRIRNQ- KQLV-NLPII ALTAKAMKND REKCIEAGAN
101       DYISKPVDVD QLLSMLRVWL
//
ID   ENBZZZ33   ami; 118 BP.
XX
AC   ARB_DC5C7E0F;
OS   EDN70752.1 (1064-1181) Beggiatoa sp. PS
XX
XX
XX
XX
XX
XX
FT   source          1..118
XX
SQ   Sequence 118 BP;
1         LIVDDDIRNT FALMTYLEGE GMEMEIFIGE NGKEALTMLD EHPDIDIVLM
51        DIMMPEMDGY EAIRAIRKQ- TRFQ-KLPII ALTAKAMKGD KTKCIEAGAN
101       DYLSKPVDTE KLISLMRVWL
//
ID   AU0ZZZZ3   ami; 116 BP.
XX
AC   ARB_72227A72;
OS   WP_039463312.1 (1249-1364) Vibrio navarrensis 0053-83
XX
XX
XX
XX
XX
XX
FT   source          1..116
XX
SQ   Sequence 116 BP;
1         LLVDDDMRNV FALSSILEDK --GMDIVIAR DGLESLDKLK ENPDIDVVLM
51        DIMMPRMDGY EAMEEIRKQ- KVYE-KLPVI ALTAKAMKGD RSKCIEAGAS
101       DYLAKPVNTD KLLSMLRVWL
//
ID   AVMZZZZ4   ami; 116 BP.
XX
AC   ARB_346AC2A5;
OS   WP_062149449.1 (1293-1408) Beggiatoa leptomitiformis D-402
```

```
XX
XX
XX
XX
XX
XX
FT   source          1..116
XX
SQ   Sequence 116 BP;
1         LLADDDVRNS FALTTFLESK --DMDVLIAE NGKEALALLE KNPNISLVLM
51        DVMMPEMDGY EAMQRIRAQ- TRFR-KLPII ALTAKAMKGD KAKCIKAGAN
101       DYLTKPVDMN RLLSLLRVWL
//
ID   AWAZZZZZ   ami; 116 BP.
XX
AC   ARB_59F0A039;
OS   WP_034640925.1 (1264-1379) Desulfovibrio inopinatus DSM 10711
XX
XX
XX
XX
XX
XX
FT   source          1..116
XX
SQ   Sequence 116 BP;
1         LVVDDDMRNV FALSNALEEK --DLNVIVAR NGQESIDKLK DHPEINLVLM
51        DIMMPVMDGY EAMRAIRKN- PQHK-KLPII ALTAKAMKGD RNLCIEAGAN
101       DYLAKPVDTE KLLSMLRVWL
//
ID   AWBZZZZZ   ami; 80 BP.
XX
AC   ARB_CC21F777;
OS   518251480_WP_019421688_1_879-958_Paenibacillus_sp._OSY-SE
XX
XX
XX
XX
XX
XX
FT   source          1..80
XX
SQ   Sequence 80 BP;
1         LIVEDDGPQR QSLIALIEGV --DVSVTAVS TGTEALKQLA DRK-FDGMVL
51        DLLLPDMTGF ELMDHISRN- PQLR-RVPII VYTGK----- ----------
101       ---------- ----------
//
ID   AWGZZZZZ   ami; 116 BP.
XX
AC   ARB_F76AC0B7;
```

```
OS   WP_046674080.1 (1025-1140) Sphingobacterium sp. Ag1
XX
XX
XX
XX
XX
XX
FT   source          1..116
XX
SQ   Sequence 116 BP;
1         LLADDDMRNI FALSTAFESY --DMNIEIAN NGQEALDILE RNEQIDLVLM
51        DIMMPVMDGY EAIEKIRAN- KKFA-NLPII AVTAKAMKGD REKTIAVGAN
101       DYISKPIDVD KLISLMRVWL
//
ID   AVOZZZZ4   ami; 116 BP.
XX
AC   ARB_56BC4E49;
OS   WP_002690554.1 (1134-1249) Beggiatoa alba B18LD
XX
XX
XX
XX
XX
XX
FT   source          1..116
XX
SQ   Sequence 116 BP;
1         LIADDDMRNT FALGTVLEKH --AMQVVIAK NGKKALEILE EQPDIDIILM
51        DIMMPEMDGY EAMRQIRSQ- PNLR-KLPII ALTAKAMKGD KGKAIEAGAN
101       DYLSKPVDVD KLISLLRVWL
//
ID   KJUZZZZ3   ami; 116 BP.
XX
AC   ARB_B41AB2DC;
OS   KJU86825.1 (1072-1187) Cand. Magnetobacterium bavaricum
XX
XX
XX
XX
XX
XX
FT   source          1..116
XX
SQ   Sequence 116 BP;
1         LIVDDDVRNV FALSNVLQDR --GVNVLVGR DGREGIARLK DNPSVELVLM
51        DIMMPEMDGY EAIRQIRSH- YEYR-NLPII ALTAKAMKED KMRCIEAGAN
101       DYLAKPIDAD RLLSLLRVWL
//
ID   AT8ZZZZ3   ami; 116 BP.
XX
```

```
AC   ARB_C7E9059F;
OS   WP_052567468.1 (1076-1191) Cand. Magnetobacterium casensis MYR-1
XX
XX
XX
XX
XX
XX
FT   source          1..116
XX
SQ   Sequence 116 BP;
1         LIVDDDVRNV FALSSVLQER --GVNVLVGR DGREGIARLK DNKAVELVLM
51        DIMMPEMDGY EAIRQIRSS- YEYR-NLPII ALTAKAMKED KMKCIEAGAN
101       DYLAKPIDAD RLLSLLRVWL
//
ID   ASJZZZ18   ami; 116 BP.
XX
AC   ARB_78930E0D;
OS   WP_052491912.1 (1585-1700) Thioploca ingrica
XX
XX
XX
XX
XX
XX
FT   source          1..116
XX
SQ   Sequence 116 BP;
1         LLVDDDMRNV FALAASLENK --GMEVITAH NGQDALARLS EHPDIAVVLM
51        DIMMPNMDGY EATRKIRAQ- PPFR-KLPII ALTAKAMKGD KAKCIEAGAN
101       DYLAKPVNTE KLISLLRVWL
//
ID   EKhpYyyy   ami; 116 BP.
XX
AC   ARB_C45CEAF3;
OS   EKD40401.1 (506-621) uncultured bacterium, subsurface aquifer
sediment
XX
XX
XX
XX
XX
XX
FT   source          1..116
XX
SQ   Sequence 116 BP;
1         LVVDDDMRNV FALANLLEEK --GVKVAVAR DGLESLARLE QNKDIDLVLM
51        DIMMPKMDGI EAMRRIREK- PEHR-ELPII ALTAKAMKGD RAKCIEAGAN
101       DYLAKPVDTD RLISILKVWL
//
```

```
ID   ABDpYyyy   ami; 116 BP.
XX
AC   ARB_B0F359DE;
OS   ABD75783.1 (846-961) uncultured bacterium, tidal flat
XX
XX
XX
XX
XX
XX
FT   source          1..116
XX
SQ   Sequence 116 BP;
1         LMVDDDIRNV FALSSVLEEK --GMTIVIGE NGKEALELLK NNPGIDLVLM
51        DIMMPEMDGY EATRQIRKQ- TQFS-KLPII ALTAKAMKGD RQKCIDAGAN
101       DYLSKPIDID KLLSLLRVWL
//
ID   AVOZZZZ6   ami; 116 BP.
XX
AC   ARB_8292F153;
OS   WP_002690178.1 (1316-1431) Beggiatoa alba B18LD
XX
XX
XX
XX
XX
XX
FT   source          1..116
XX
SQ   Sequence 116 BP;
1         LLVDDDVRNS FALTTFLESK --DMDVVVAE NGKEALDALT KHPNISLVLM
51        DIMMPEMDGY EAMKHIREQ- TKFR-KLPII ALTAKAMKGD KAKCIAAGAN
101       DYLTKPVDMN RLLSLLRVWL
//
ID   AVMZZZZ7   ami; 116 BP.
XX
AC   ARB_5F71452D;
OS   WP_062154998.1 (1132-1247) Beggiatoa leptomitiformis D-402
XX
XX
XX
XX
XX
XX
FT   source          1..116
XX
SQ   Sequence 116 BP;
1         LIADDDMRNT FALGTVLEKN --NMLVIIAK NGKKALEILQ EQHDIDIVLM
51        DIMMPEMDGY EAMRQIRAQ- PTFR-KLPII ALTAKAMKGD RVKAIEAGAN
101       DYLSKPVDVD KLISLLRVWL
```

```
//
ID   AWPZZZZZ   ami; 116 BP.
XX
AC   ARB_E9976B5F;
OS   WP_028072569.1 (1023-1138) Sphingobacterium thalpophilum DSM
11723
XX
XX
XX
XX
XX
XX
FT   source          1..116
XX
SQ   Sequence 116 BP;
1         LLADDDMRNI FALSTVFEGY --EINVEIAN NGQEALDILE RNEHIDLVLM
51        DIMMPVMDGY EAIENIRAN- KRFA-NLPII AVTAKAMKGD REKTIAVGAN
101       DYISKPVDVD KLISLMRVWL
//
ID   GAbZZZZ2   ami; 116 BP.
XX
AC   ARB_7EB6FB57;
OS   GAK52359.1 (353-468) bacterium UASB14
XX
XX
XX
XX
XX
XX
FT   source          1..116
XX
SQ   Sequence 116 BP;
1         LVVDDDMRNV FALSSVLEGK --GMEVLIAE NGKEALDQLD AHPEINLVLM
51        DIMMPEMNGY ETMERIRSQ- AKFE-KLPMI ALTAKAMKGD RQKALDAGAN
101       DYLSKPIDID KLLSLLRVWL
//
ID   WtknYyyy   ami; 116 BP.
XX
AC   ARB_3C691B65;
OS   WP_017422776.1 (1254-1369) Vibrio vulnificus ATCC 27562
XX
XX
XX
XX
XX
XX
FT   source          1..116
XX
SQ   Sequence 116 BP;
1         LLVDDDMRNV FALSSILEDK --GMDIVIAR DGVESLDKLK ENPDIDVVLM
```

```
51        DIMMPKMDGY EAMEEIRKQ- KVYE-KLPVI ALTAKAMKGD RSKCIEAGAS
101       DYLAKPVNTD KLLSMLRVWL
//
ID   KZLZZZZZ   ami; 116 BP.
XX
AC   ARB_BC8301D3;
OS   KZL89025.1 (102-217) DivK Clostridium magnum DSM 2767
XX
XX
XX
XX
XX
XX
FT   source          1..116
XX
SQ   Sequence 116 BP;
1         LIVDDDMRNV FALTSALEEK --GISVIVGR NGVEGIKKLQ ENPDTDLILM
51        DVMMPEMDGY AAMRKIRKE- ETFK-NIPII AITAKAMKDD REKCIEAGAN
101       DYLTKPVDIN KLISLLRVWL
//
ID   EKtYyyyy   ami; 116 BP.
XX
AC   ARB_C392A8F8;
OS   EKD34307.1 (178-293) uncultured bacterium
XX
XX
XX
XX
XX
XX
FT   source          1..116
XX
SQ   Sequence 116 BP;
1         LLVDDDMRNV FAISSILEDK --GLTITICK NGREALQALD EHPTARLVLM
51        DMMMPEMDGY QAMGEIRKQ- KRFE-KLPII AITAKAMMGD RCKCIEAGAS
101       DYLAKPIDRD KLLSMMRVWL
//
ID   AXCZZZZ3   ami; 116 BP.
XX
AC   ARB_70EA0A6F;
OS   AGX87357.1 (1263-1378) Cand. Symbiobacter mobilis CR
XX
XX
XX
XX
XX
XX
FT   source          1..116
XX
SQ   Sequence 116 BP;
```

```
1         LLVDDDMRNV FALSAALQEQ --DMQVIPAA NGIEALKLLD EHPDVSLVLM
51        DIMMPEMDGY EAMRRIRAQ- DRFA-SLPII ALTAKAMKGD RSKCIEAGAS
101       DYLAKPVDSA KLISMMRVWL
//
ID   AUYZZZZ2   ami; 82 BP.
XX
AC   ARB_D901CEC2;
OS   655083960_WP_028532005_1_868-949_Paenibacillus_sp._UNC217MF
XX
XX
XX
XX
XX
XX
FT   source          1..82
XX
SQ   Sequence 82 BP;
1         LIVEDDGPQR QSLIALIEGA --DVAVTAVS TGTEALKVLG EEN-FDGMVL
51        DLLLPDMTGF ELMDEISHH- SRIR-RIPII VYTGKLL--- ----------
101       ---------- ----------
//
ID   AX0ZZZZZ   ami; 116 BP.
XX
AC   ARB_E9415C9A;
OS   WP_048907458.1 (1041-1156) Pedobacter sp. V48
XX
XX
XX
XX
XX
XX
FT   source          1..116
XX
SQ   Sequence 116 BP;
1         LITDDDMRNI FALSSALQLY --DMNIIIAN NGREALERLA ENEEIDLVLM
51        DIMMPEMDGY EAMKAIRSE- KRFS-KLPII ALTAKAMKND REKCIEAGAN
101       DYISKPVDMD KLLSMLRVWL
//
ID   A0AWPyyy   ami; 116 BP.
XX
AC   ARB_99B4AB2;
OS   WP_008246697.1 (1044-1159) Pedobacter sp. BAL39
XX
XX
XX
XX
XX
XX
FT   source          1..116
XX
```

```
SQ   Sequence 116 BP;
1         LITDDDMRNI FALSSALQVY --DLKIIIAN NGREALEKLA GPDTIDIVLM
51        DIMMPEMDGY EAMKAIRME- KRFA-KLPII ALTAKAMKND REKCIEAGAN
101       DYISKPVDMD KLLSMLRVWL
//
ID   AX8ZZZZZ   ami; 116 BP.
XX
AC   ARB_9A70A02D;
OS   WP_028586737.1 (1297-1412) Desulfocurvus vexinensis DSM 17965
XX
XX
XX
XX
XX
XX
FT   source          1..116
XX
SQ   Sequence 116 BP;
1         LLVDDDMRNV FALSSALEAH --GMEVAIAR NGREGVEKLS ANPETDLVLM
51        DIMMPEMDGY EAMRAIRKR- KRFQ-DLPII ALTAKAMKGD RNKCIEAGAS
101       DYMAKPVDTD KLFSLLRVWL
//
ID   ASJZZZ25   ami; 116 BP.
XX
AC   ARB_9F3756DE;
OS   WP_052491805.1 (1354-1469) Thioploca ingrica
XX
XX
XX
XX
XX
XX
FT   source          1..116
XX
SQ   Sequence 116 BP;
1         LLVDDDVRNT FALATVLEDN --DMEIIVAT NGKEALRKLN ENKDIVMVIM
51        DMMMPEMDGY EAMRTIRTQ- PNFH-NLPII ALTAKAMKGD KAKCIEAGAN
101       DYLSKPVNTD KLISLMRVWL
//
ID   AXAZZZZZ   ami; 116 BP.
XX
AC   ARB_1169B3EE;
OS   WP_012141995.1 (1258-1373) Shewanella sediminis HAW-EB3
XX
XX
XX
XX
XX
XX
FT   source          1..116
```

```
XX
SQ   Sequence 116 BP;
1         LLVDDDMRNV FALSSILEDK --GIEIVVGR DGLESIEKLD QHPDIALILM
51        DIMMPKMDGY DAMKEIRKK- RKYK-KLPII ALTAKAMKGD RSKCIEAGAS
101       DYLAKPVDTD KLLSMLRVWL
//
ID   AVWZZZZ4   ami; 80 BP.
XX
AC   ARB_83EAF85A;
OS   544846345_WP_021261593_1_868-947_Paenibacillus_alvei
XX
XX
XX
XX
XX
XX
FT   source          1..80
XX
SQ   Sequence 80 BP;
1         LIVEDDGPQR QSLIALIEGA --DVAVTAVS TGTEALKVLG EEK-FDGMVL
51        DLLLPDMTGF ELMDEISHH- SRIR-RIPII VYTGK----- ----------
101       ---------- ----------
//
ID   YPOYyyyy   ami; 116 BP.
XX
AC   ARB_5EC37475;
OS   AF046015.1 (290-405) Pseudomonas putida DOT-T1E (ident. to GM84)
XX
XX
XX
XX
XX
XX
FT   source          1..116
XX
SQ   Sequence 116 BP;
1         LLVDDDVRNI FALTSALEHK --GAIVEIGR NGREAIERLE QHDDIDLVLM
51        DVMMPEMDGF EATRLIRQQ- PRWR-KLPII AVTAKAMKDD QQRCLQAGAN
101       DYLAKPIDLD RLFSLIRVWL
//
ID   KJRZZZZ3   ami; 116 BP.
XX
AC   ARB_3F1372A0;
OS   KJR99450.1 (1257-1372) Desulfobulbaceae bacterium BRH_c16a
XX
XX
XX
XX
XX
XX
```

```
FT   source          1..116
XX
SQ   Sequence 116 BP;
1         LVVDDDMRNV FALTNLLEEK --GVKIVVAR DGLESLERLE HNKEIDLVLM
51        DIMMPKMDGL EAMRRIREN- PEFR-DLPII ALTAKAMKGD RTKCIEAGAN
101       DYLAKPVDTD RLISILKVWL
//
ID   YGtdYyyy   ami; 116 BP.
XX
AC   ARB_AB43A98D;
OS   WP_012805237.1 (1238-1353) Desulfomicrobium baculatum DSM 4028
XX
XX
XX
XX
XX
XX
FT   source          1..116
XX
SQ   Sequence 116 BP;
1         LVVDDDMRNI FALTSVLEEK --GMQVVVAR DGSESLTRLR ENPEIDLVLM
51        DIMMPVMDGY EAMREIRKD- PKLK-DLPII ALTAKAMKGD KNACIEAGAN
101       DYLAKPVDMD KLLSLLRVWL
//
ID   YGAFYyyy   ami; 116 BP.
XX
AC   ARB_4B659EB8;
OS   WP_015759177.1 (1038-1153) Desulfotomaculum acetoxidans DSM 771
XX
XX
XX
XX
XX
XX
FT   source          1..116
XX
SQ   Sequence 116 BP;
1         LLIDDDMRNV FALMNVLEEK --GMKVLVGK NGKEGIFLLE DNPDVDLILM
51        DIMMPEMDGY EAMKEIRKQ- NKFK-LTPII ALTAKAMVGD RSKCIEAGAS
101       DYLSKPIDTD KLLSLLRVWL
//
ID   AXVZZZZZ   ami; 116 BP.
XX
AC   ARB_BE1F6A6B;
OS   WP_045434835.1 (1084-1199) bacterium UASB270
XX
XX
XX
XX
XX
```

```
XX
FT   source          1..116
XX
SQ   Sequence 116 BP;
1         LLVDDDVRNI FALSSVLEDK --GLSVLAAG NGREALDMLA SHPEIDLVLM
51        DIMMPEMDGY ETMQHIRKQ- PEFS-KLPII ALTAKAMKGD RQRCIEAGAS
101       DYLSKPVESE KLLSLLRVWL
//
ID   AXWZZZZZ   ami; 116 BP.
XX
AC   ARB_6D6A05A2;
OS   WP_061984873.1 (1226-1341) Flammeovirgaceae bacterium 311
XX
XX
XX
XX
XX
XX
FT   source          1..116
XX
SQ   Sequence 116 BP;
1         LIVDDDVRNV YSLCSLLENH --EMNIVVAY DGQEALNKLE AVEDIDIVLM
51        DVMMPEMDGI EATRQIRQN- YKFR-KLPII ALTAKAMKGD KEKCIEAGAS
101       DYIPKPVDTD KLLTLMRVWL
//
ID   CBXhYyyy   ami; 116 BP.
XX
AC   ARB_B59F5AB2;
OS   CBX27662.1 (901-1016) uncultured Desulfobacterium sp.
XX
XX
XX
XX
XX
XX
FT   source          1..116
XX
SQ   Sequence 116 BP;
1         LIVDDDMRNV FAVSNIIEEK --GMEVLVGK NGREGLERLN GNPDIDLVLM
51        DIMMPEMNGY EAMTEIRKQ- MRFK-SLPII ALTAKAMTGE RNKCLEAGAS
101       DYLAKPFDID KLLSLMRVWL
//
ID   NPsiYyyy   ami; 116 BP.
XX
AC   ARB_C1F8F28A;
OS   WP_045622491.1 (1254-1369) Vibrio vulnificus SC9794
XX
XX
XX
XX
```

```
XX
XX
FT   source          1..116
XX
SQ   Sequence 116 BP;
1         LLVDDDMRNV FALSSILEDK --GMDIVIAR DGLESLDKLK ENPDIDVVLM
51        DIMMPKMDGY EAMEEIRKQ- KVYE-KLPVI ALTAKAMKGD RSKCIEAGAS
101       DYLAKPVNTD KLLSMLRVWL
//
ID   ASYZZZ14   ami; 116 BP.
XX
AC   ARB_5C0F038B;
OS   WP_039546606.1 (1254-1369) Vibrio vulnificus 101/4
XX
XX
XX
XX
XX
XX
FT   source          1..116
XX
SQ   Sequence 116 BP;
1         LLVDDDMRNV FALSSILEDK --GMDIVIAR DGIESLDKLK ENPDIDVVLM
51        DIMMPKMDGY EAMEEIRKQ- KVYE-KLPVI ALTAKAMKGD RSKCIEAGAS
101       DYLAKPVNTD KLLSMLRVWL
//
ID   YPzpYyyy   ami; 116 BP.
XX
AC   ARB_40DC206B;
OS   WP_015415991.1 (1277-1392) Desulfovibrio piezophilus C1TLV30
XX
XX
XX
XX
XX
XX
FT   source          1..116
XX
SQ   Sequence 116 BP;
1         LLVDDDMRNV FALSSVLEEK --TMDVVIAR NGLEAIDKLK EHDEIDLVLM
51        DIMMPLMDGY EAMQAIRKE- HKYA-KLPMI ALTAKAMKGD RSKCIEAGAN
101       DYLAKPVNTD KLLSMLRVWL
//
```
