# Supplementary material for: Visualizing Evolutionary Relationships of Multidomain Proteins: An Example from Receiver (REC) Domains of Sensor Histidine Kinases in the Candidatus Maribeggiatoa str. Orange Guaymas Draft Genome
Source: Front Microbiol. 2016 Nov 14;7:1780. doi: 10.3389/fmicb.2016.01780 (PMC5108060; doi:10.3389/fmicb.2016.01780)
Supplement: Supplementary file 7 [file DataSheet5.PDF]

```
ID   RsnZZZZZ   ami; 116 BP.
XX
AC   ARB_79B27B4D;
OS   BOGUAY 00153_2324 (70-186) K1
XX
XX
XX
XX
XX
XX
FT   source          1..116
XX
SQ   Sequence 116 BP;
1         LIIEDDQNFS SLLVDLAHNK GFKCLIAGDG ITGLQ-LAEE YKPHAIILDV
51        GLPQLDGWTV MEKLKDNPAI RHIPVHFMSA AEQQNMDAQK MGAIGYLLKP
101       VSMEQLGEAF QKIELFL... .......... .......... ..........
151       .......... .......... .......... .......... ..........
201       .......... .......... .......... ..........
//
ID   RssZZZZZ   ami; 115 BP.
XX
AC   ARB_5F0717E9;
OS   BOGUAY 00286_0624 (70-193) (J1)
XX
XX
XX
XX
XX
XX
FT   source          1..115
XX
SQ   Sequence 115 BP;
1         LIIEDDHNFS NILIELAQEK GFKYLIAKDG ETGLQ-LAQE YHPQAVILDV
51        GLPQLDGISV MERLKDNPNT RAIPVHFISA SD-QSSEAKK MGAIGYLIKP
101       VSVDEKEMEV LVADNGK... .......... .......... ..........
151       .......... .......... .......... .......... ..........
201       .......... .......... .......... ..........
//
ID   RsYZZZZZ   ami; 115 BP.
XX
AC   ARB_667AA1F8;
OS   BOGUAY 01204_2878 (135-250) (M1)
XX
XX
XX
XX
XX
XX
FT   source          1..115
XX
```

```
SQ   Sequence 115 BP;
1         LIIEDDSKFS EILLKLAQEK GFKCIIAEDG QTGLQ-LAEE YRPSAIILDI
51        GLPKIDGWAV MERLKDNPNT RHIPVHFISA SD-QEKPAKT MGAIGYLLKP
101       VNMTELSEAF KKIEGFI... .......... .......... ..........
151       .......... .......... .......... .......... ..........
201       .......... .......... .......... ..........
//
ID   RsBZZZZZ   ami; 115 BP.
XX
AC   ARB_9C427493;
OS   BOGUAY 00806_2995 (1111-1226) L1
XX
XX
XX
XX
XX
XX
FT   source          1..115
XX
SQ   Sequence 115 BP;
1         LIVEDDRNFS HLVMELAQEK GFKCIIAEDG KTGLE-FAEQ YQPHAIILDL
51        GLPKVDGWTV MEKLKDNAQT RHIPVHFMSG SD-QSMEAKK MGAIGYLLKP
101       VSMEQLGGAF QKIEQFI... .......... .......... ..........
151       .......... .......... .......... .......... ..........
201       .......... .......... .......... ..........
//
ID   ENBZZZZZ   ami; 115 BP.
XX
AC   ARB_D6803E74;
OS   EDN70752.1 (796-910) Beggiatoa sp. PS
XX
XX
XX
XX
XX
XX
FT   source          1..115
XX
SQ   Sequence 115 BP;
1         LLIEDDYKFS RLLMEFARDR GFKCLIAGDG KTGLQ-LAQQ YKPHAIILDV
51        GLPQMDGWTV MEKLKDNPET RHIPVHFLSA SE-SNLDAKK MGAIGYLLKP
101       VGMGEINEAF KNIEQFL... .......... .......... ..........
151       .......... .......... .......... .......... ..........
201       .......... .......... .......... ..........
//
ID   ENBZZZZ3   ami; 115 BP.
XX
AC   ARB_6D753801;
OS   EDN71014.1 (1223-1337) Beggiatoa sp. PS
XX
```

```
XX
XX
XX
XX
XX
FT   source          1..115
XX
SQ   Sequence 115 BP;
1         LIVEDDRKFS TLILELARET GFKCLVAEDG ITGLQ-MAEQ YKPHAIILDV
51        GLPNLDGWKV MEQLKNNPDT RHIPVHFISA FD-QSLEAKK MGAIGYLMKP
101       VNMEQLGETF QKIEYFM... .......... .......... ..........
151       .......... .......... .......... .......... ..........
201       .......... .......... .......... ..........
//
ID   ENBZZZZ5   ami; 116 BP.
XX
AC   ARB_97FCD652;
OS   EDN68655.1 (437-552) Beggiatoa sp. PS
XX
XX
XX
XX
XX
XX
FT   source          1..116
XX
SQ   Sequence 116 BP;
1         LIIEDDRKFI SILMELAHEK GFKCLVAENG QTGLQ-LVEE YQPNAIILDV
51        GLPQLDGWTV MGILKDDPKT RHIPVHFISA ADHNILEATR MGAIGYLQKP
101       VNLEQLGETF QKIEQFM... .......... .......... ..........
151       .......... .......... .......... .......... ..........
201       .......... .......... .......... ..........
//
ID   ASJZZZZZ   ami; 115 BP.
XX
AC   ARB_33CE3F15;
OS   WP_052492084.1 (880-994) Thioploca ingrica
XX
XX
XX
XX
XX
XX
FT   source          1..115
XX
SQ   Sequence 115 BP;
1         LIIEDDRKFA TILVELAREK EFKCLFAEDG KTGLQ-LAEK YHPHAIILDV
51        GLPLLDGWTV MEQLKDNPNT RHIPVHFMSA TE-QGSEAKK MGAIGYLLKP
101       ISMAELGDAF KKIEQFL... .......... .......... ..........
151       .......... .......... .......... .......... ..........
```

```
201       .......... .......... .......... ..........
//
ID   ASJZZZZ3   ami; 115 BP.
XX
AC   ARB_5E3A9CC7;
OS   WP_052492165.1 (1070-1184) Thioploca ingrica
XX
XX
XX
XX
XX
XX
FT   source          1..115
XX
SQ   Sequence 115 BP;
1         LITEDDRRFA NLLMEQAREK GFKCIIAEDG TTGLE-LAEK YRPHVIMLDI
51        GLPKTDGWTV MERLKDNPEI RHIPVYFMSA AD-QNLAAKK LGAIGYLLKP
101       VSMEQLTEAF NQIEQFL... .......... .......... ..........
151       .......... .......... .......... .......... ..........
201       .......... .......... .......... ..........
//
ID   ENBZZZZ8   ami; 120 BP.
XX
AC   ARB_9D751445;
OS   EDN71011.1 (929-1048) Beggiatoa sp. PS
XX
XX
XX
XX
XX
XX
FT   source          1..120
XX
SQ   Sequence 120 BP;
1         LIVEDDPKFA RLMVELAHEQ QFKCLVGEDG LTGLQ-LAEQ YNPHAIILDV
51        GLPHLDGWKV MEQLKNNPDT RHIPVHFISA FD-QSLAAKK MGAIGYLLKP
101       VSMEQLSETF QKIETFMANT VK........ .......... ..........
151       .......... .......... .......... .......... ..........
201       .......... .......... .......... ..........
//
ID   ASKZZZZZ   ami; 115 BP.
XX
AC   ARB_660A04E9;
OS   WP_062153829.1 (992-1106) Beggiatoa leptomitiformis D-402
XX
XX
XX
XX
XX
XX
```

```
FT   source          1..115
XX
SQ   Sequence 115 BP;
1         LIIEDDRKFL KILMDLAREK DFKCLVAEDG KDGLE-LAQT YKPHAIILDV
51        GLPKVDGWTV MERLKENPET RHIPVHFMSG AD-QEREAKK MGAIGYLLKP
101       ISLSELGEAF KKIERFI... .......... .......... ..........
151       .......... .......... .......... .......... ..........
201       .......... .......... .......... ..........
//
ID   ASLZZZZZ   ami; 115 BP.
XX
AC   ARB_C8F4D6AB;
OS   WP_002684997.1 (1002-1116) Beggiatoa alba B18LD
XX
XX
XX
XX
XX
XX
FT   source          1..115
XX
SQ   Sequence 115 BP;
1         LIIEDDQKFL KILMDLAREK DFKCLIAEDG KDGLE-LAQT YKPNAIILDV
51        GLPKVDGWTV MERLKENPDT RHIPVHFMSG AD-QEREAKK MGAIGYLLKP
101       ISLSELGEAF KKIERFI... .......... .......... ..........
151       .......... .......... .......... .......... ..........
201       .......... .......... .......... ..........
//
ID   ENgZZZZZ   ami; 116 BP.
XX
AC   ARB_9F388D;
OS   EDN72941.1 (355-470) Beggiatoa sp. SS
XX
XX
XX
XX
XX
XX
FT   source          1..116
XX
SQ   Sequence 116 BP;
1         LVIEDDPKFS SILLGLGREK NFKCLLAEDG RTGLQ-LAEE YWPKAIILDV
51        GLPNLNGWSV LERLKDNPET RHIPVHFISG AEQNTLNATK MGAIGFLQKP
101       VNMAQLGEVF KKIEQFI... .......... .......... ..........
151       .......... .......... .......... .......... ..........
201       .......... .......... .......... ..........
//
ID   ASJZZZZ5   ami; 115 BP.
XX
AC   ARB_9A3AD17F;
```

```
OS   WP_052491805.1 (1086-1200) Thioploca ingrica
XX
XX
XX
XX
XX
XX
FT   source          1..115
XX
SQ   Sequence 115 BP;
1         LIIEDDSRFS RLLRENAQEK GFKYLLAEEG RTGLQ-LAQY YQPHAIILDV
51        SLPQVDGWTV MEKLKDNPHT RHIPVHFISA AD-QEMTAKR MGAIGYLLKP
101       VNLSELEEAF KKIERFL... .......... .......... ..........
151       .......... .......... .......... .......... ..........
201       .......... .......... .......... ..........
//
ID   ENpZZZZZ   ami; 115 BP.
XX
AC   ARB_826065F3;
OS   EDN68110.1 (331-445) Beggiatoa sp. PS
XX
XX
XX
XX
XX
XX
FT   source          1..115
XX
SQ   Sequence 115 BP;
1         LIIEDERKFS RLLMDLAQEN AFKCLVAEDG ETSLQ-LAEQ YKPHAIILDV
51        GLPQVDGWTV MERLKDNQDT RHIPVYFISA SD-QVQEAKK MGAIGYLLKP
101       VGMAELEEAF QNIKQFI... .......... .......... ..........
151       .......... .......... .......... .......... ..........
201       .......... .......... .......... ..........
//
ID   ENpZZZZ3   ami; 115 BP.
XX
AC   ARB_FE65E809;
OS   EDN68471.1 (461-575) Beggiatoa sp. PS
XX
XX
XX
XX
XX
XX
FT   source          1..115
XX
SQ   Sequence 115 BP;
1         LVIEDDRKFS TILMDVAHNK DFQCLIAEDG QTGLQ-FAQT YQPNAIILDI
51        GLPQMDGWMV MEQLKDHSDT RHIPVHFLSA FN-QSLKAKK MGALGYLVKP
```

```
101       INIEQLGDVF RKFEQFF... .......... .......... ..........
151       .......... .......... .......... .......... ..........
201       .......... .......... .......... ..........
//
ID   ASJZZZZ6   ami; 115 BP.
XX
AC   ARB_DA66F251;
OS   WP_052491909.1 (872-986) Thioploca ingrica
XX
XX
XX
XX
XX
XX
FT   source          1..115
XX
SQ   Sequence 115 BP;
1         LIVEDDHSFA QVLMEIAHEK FFKCLLATDG DMGLQ-LAAT YQPHAIILDL
51        GLPQVDGWSV MEQLKDNPVT RHIPVHFVSG TD-ESKDAKK MGAIGYCLKP
101       VSMAQLSDAF KHIETFI... .......... .......... ..........
151       .......... .......... .......... .......... ..........
201       .......... .......... .......... ..........
//
ID   ASLZZZZ3   ami; 115 BP.
XX
AC   ARB_2F588861;
OS   WP_002690178.1 (1048-1162) Beggiatoa alba B18LD
XX
XX
XX
XX
XX
XX
FT   source          1..115
XX
SQ   Sequence 115 BP;
1         LIVEDDRKFS TILKELATEK GFKCLLAEDG KEALQ-LAEM YLPCAIILDV
51        GLPLLDGWTV MERLKDSPLT RHIPIHVMSA SE-QSRDARK LGAIGYLLKP
101       VSMNELSDSF RKIETFI... .......... .......... ..........
151       .......... .......... .......... .......... ..........
201       .......... .......... .......... ..........
//
ID   ENBZZZ10   ami; 115 BP.
XX
AC   ARB_F270104E;
OS   EDN71132.1 (749-863) Beggiatoa sp. PS
XX
XX
XX
XX
```

```
XX
XX
FT   source          1..115
XX
SQ   Sequence 115 BP;
1         LIIEDDRRFS HLLLESARET GFKCLLAEEG RMGLE-LAET HKPNAIILDI
51        GLPSIDGWTV MEHLKDHPDT RHIPVHFISA FD-QRLEAKK MGAIGYLIKP
101       VSLEQLDETF QKIEQFL... .......... .......... ..........
151       .......... .......... .......... .......... ..........
201       .......... .......... .......... ..........
//
ID   ASKZZZZ3   ami; 115 BP.
XX
AC   ARB_34E75208;
OS   WP_062149449.1 (1025-1139) Beggiatoa leptomitiformis D-402
XX
XX
XX
XX
XX
XX
FT   source          1..115
XX
SQ   Sequence 115 BP;
1         LIVEDDRKFS TILKEIAVEK GFKCLLAEDG KTALQ-LAET YLPSAIILDV
51        GLPLLDGWTV MERLKDNAIT RHIPIHIMSA SE-QSKDARK LGAIGYLLKP
101       VSMNELSDSF KKIEHFV... .......... .......... ..........
151       .......... .......... .......... .......... ..........
201       .......... .......... .......... ..........
//
ID   ASKZZZZ5   ami; 120 BP.
XX
AC   ARB_3E2B09EC;
OS   WP_062154998.1 (864-983) Beggiatoa leptomitiformis D-402
XX
XX
XX
XX
XX
XX
FT   source          1..120
XX
SQ   Sequence 120 BP;
1         LIIEDDPTFA KLLQEIAQEK GFKALLASDG KAGLQ-LAQT YQPHAIILDV
51        GLPLIDGWTV MECLKDNANT RHIPVHFMSA TD-QDKDARQ MGAIGYLLKP
101       ISMEELSDSF KRIEQFIAKT LK........ .......... ..........
151       .......... .......... .......... .......... ..........
201       .......... .......... .......... ..........
//
ID   ASLZZZZ5   ami; 115 BP.
```

```
XX
AC   ARB_3463A477;
OS   WP_002690554.1 (866-980) Beggiatoa alba B18LD
XX
XX
XX
XX
XX
XX
FT   source          1..115
XX
SQ   Sequence 115 BP;
1         LIIEDDPVFA QLLQDIAQEK GFKSLLANDG KTGLQ-LAQD YQPQAIILDL
51        GLPQIDGWSV MERLKDNANT RHIPIHIMSA TD-QDKDARQ MGAIGYLLKP
101       ISMEELGNSF KYIEHFI... .......... .......... ..........
151       .......... .......... .......... .......... ..........
201       .......... .......... .......... ..........
//
ID   ASMZZZZZ   ami; 115 BP.
XX
AC   ARB_A0CA6C28;
OS   WP_034640925.1 (996-1110) Desulfovibrio inopinatus DSM 10711
XX
XX
XX
XX
XX
XX
FT   source          1..115
XX
SQ   Sequence 115 BP;
1         LVIEDDSTFA QVMRDFARER GFKCVVAEDG ETGLH-FADY FKPSAIILDI
51        GLPGIDGWTV MERLKDNPSL RHIPVHFMSA SD-SSMDAMR MGAVGFLTKP
101       VSMEKIQEAF SKIERII... .......... .......... ..........
151       .......... .......... .......... .......... ..........
201       .......... .......... .......... ..........
//
ID   ASOZZZZZ   ami; 115 BP.
XX
AC   ARB_1B63E5C5;
OS   WP_011189034.1 (974-1088) Desulfotalea psychrophila LSv54
XX
XX
XX
XX
XX
XX
FT   source          1..115
XX
SQ   Sequence 115 BP;
```

```
1         LIIEDDENFS RVLRDIGRDR GFACILASDG ETGLA-LADH YTPSAIILDI
51        GLPGIDGWTV MERLKDNSKL RHIPVHFMSA SD-SSMDAMR MGAIGYLTKP
101       VSMEKVEETL AKLENVI... .......... .......... ..........
151       .......... .......... .......... .......... ..........
201       .......... .......... .......... ..........
//
ID   00ZZZZ20   ami; 115 BP.
XX
AC   ARB_A358F679;
OS   GAK87599.1 (999-1113) Vibrio ponticus JCM 19238
XX
XX
XX
XX
XX
XX
FT   source          1..115
XX
SQ   Sequence 115 BP;
1         LIIEDDRAFA GVMRDFGRER GFKCIVAETG ETGLH-FAEY YKPSAIILDI
51        GLPGIDGWTV MERLKDNPAV RHIPVHFMSA ND-ANLDAMR MGAIGYLTKP
101       VDMKKLDTAF ASIENII... .......... .......... ..........
151       .......... .......... .......... .......... ..........
201       .......... .......... .......... ..........
//
ID   ASPZZZZZ   ami; 115 BP.
XX
AC   ARB_12FF7275;
OS   WP_028586737.1 (1029-1143) Desulfocurvus vexinensis DSM 17965
XX
XX
XX
XX
XX
XX
FT   source          1..115
XX
SQ   Sequence 115 BP;
1         LIVEDDPNFA RILVDMAHDR GFKCLVAGDG ETGLH-FADY YRPSAVVLDI
51        GLPGIDGWTV MDRLKSNPEL RHIPVHFMSA AD-ETLDAMR MGAVGYLTKP
101       VTLDAVNKAL ARIEGVL... .......... .......... ..........
151       .......... .......... .......... .......... ..........
201       .......... .......... .......... ..........
//
ID   ASJZZZZ8   ami; 115 BP.
XX
AC   ARB_5FDD2945;
OS   WP_052491910.1 (1199-1313) Thioploca ingrica
XX
XX
```

```
XX
XX
XX
XX
FT   source          1..115
XX
SQ   Sequence 115 BP;
1         LIVEDDERFS RLLMELAREK GFKCLLATEG QIGLQ-LAEK YKPSAIILDI
51        GLPQMNGWKV MSRLKENSLT RHIPVHFVSG TD-QSQEAKQ MGAIGYCLKP
101       VSMDELSNAF KHIENFL... .......... .......... ..........
151       .......... .......... .......... .......... ..........
201       .......... .......... .......... ..........
//
ID   ASQZZZZZ   ami; 115 BP.
XX
AC   ARB_11B88583;
OS   WP_015415991.1 (1009-1123) Desulfovibrio piezophilus C1TLV30
XX
XX
XX
XX
XX
XX
FT   source          1..115
XX
SQ   Sequence 115 BP;
1         LIIEDDVNFA KIMRDFAQER SFKCIVAEDG ETGLH-FADF YKPSAIILDI
51        GLPGIDGWTV MERLKDNPEL RHIPVHFMSA AD-SSLDAMR MGAVGFLSKP
101       VSLDKVADAF GRIENVI... .......... .......... ..........
151       .......... .......... .......... .......... ..........
201       .......... .......... .......... ..........
//
ID   ASUZZZZZ   ami; 115 BP.
XX
AC   ARB_9BEFAF8F;
OS   WP_028109261.1 (970-1084) Ferrimonas futtsuensis DSM 18154
XX
XX
XX
XX
XX
XX
FT   source          1..115
XX
SQ   Sequence 115 BP;
1         LIIEDDKAFA KVMRDFGRER GFKCIVASDG ETGLH-FADY YKPSAIILDI
51        GLPGIDGWTV MERLKENPAL RHIPVHFMSA ND-STLDAMR MGAIGYLTKP
101       VNLNEMESAF GKIEEII... .......... .......... ..........
151       .......... .......... .......... .......... ..........
201       .......... .......... .......... ..........
```

```
//
ID   ASVZZZZZ   ami; 115 BP.
XX
AC   ARB_FA81D242;
OS   WP_020000022.1 (1005-1119) Desulfovibrio desulfuricans ATCC 29578
XX
XX
XX
XX
XX
XX
FT   source          1..115
XX
SQ   Sequence 115 BP;
1         LIIEDDWNFA KIMRDFGRER GFLCLVAEDG ETGLH-FADY YKPSAIILDV
51        GLPGIDGWTV MERLKDNPAL RHIPVHFMSA ND-NSLDALR MGAIGYLAKP
101       VTMEHVEEAF GALEDVI... .......... .......... ..........
151       .......... .......... .......... .......... ..........
201       .......... .......... .......... ..........
//
ID   AtlZZZZZ   ami; 115 BP.
XX
AC   ARB_14F2AB09;
OS   ABD75783.1 (578-692) uncultured bacterium, tidal flat
XX
XX
XX
XX
XX
XX
FT   source          1..115
XX
SQ   Sequence 115 BP;
1         LIIEDDPKFA KILFDLAREK GFKGLIAGDG AAGLQ-LADQ YSPVAIILDI
51        GLPGMDGWAV MEKLKKNPDT RHIPVHFMSA QD-TPLEAMK MGAIGYLTKP
101       VTLDILNDAF NTIEETI... .......... .......... ..........
151       .......... .......... .......... .......... ..........
201       .......... .......... .......... ..........
//
ID   ASWZZZZZ   ami; 115 BP.
XX
AC   ARB_489A99B4;
OS   WP_061058081.1 (986-1100) Vibrio vulnificus ATL 6-1306
XX
XX
XX
XX
XX
XX
FT   source          1..115
```

```
XX
SQ   Sequence 115 BP;
1          LIIEDDRAFA SVMRDFGRER GFKCIVAETG ETGLH-FAQY YKPSAIILDI
51         GLPGIDGWTV MERLKENPET RHIPVHFMSA ND-ANLDALR MGAIGYLTKP
101        VDMKKLEKAF GNIEDIL... .......... .......... ..........
151        .......... .......... .......... .......... ..........
201        .......... .......... .......... ..........
//
ID   ASJZZZ10   ami; 115 BP.
XX
AC   ARB_64B46AE4;
OS   WP_052491912.1 (1316-1430) Thioploca ingrica
XX
XX
XX
XX
XX
XX
FT   source          1..115
XX
SQ   Sequence 115 BP;
1          LIIEDDVNFS HILMGIAQEK QFKCLLAHDG YSGLQ-LAEQ YRPSAIILDI
51         GLPQIDGWTV MEKLKGKPET RHIPVQFVSG SD-QSKEAKQ LGAIGYCLKP
101        VGMTELTKIF KNIDNFI... .......... .......... ..........
151        .......... .......... .......... .......... ..........
201        .......... .......... .......... ..........
//
ID   AT2ZZZZZ   ami; 115 BP.
XX
AC   ARB_498945C9;
OS   WP_015759177.1 (770-884) Desulfotomaculum acetoxidans DSM 771
XX
XX
XX
XX
XX
XX
FT   source          1..115
XX
SQ   Sequence 115 BP;
1          LIIEDDPRFS AILVDLAGGK GFHVYTAADG ETGLQ-FALK YRPSAIILDV
51         GLPDIDGWAV MERLKGNPET RHIPVHFISA YD-SGLDAMR MGAIGYLTKP
101        VSMEVLDGAF NKIQDII... .......... .......... ..........
151        .......... .......... .......... .......... ..........
201        .......... .......... .......... ..........
//
ID   AtlZZZZ4   ami; 115 BP.
XX
AC   ARB_91E2402C;
OS   ABD75785.1 (939-1053) uncultured bacterium, tidal flat
```

```
XX
XX
XX
XX
XX
XX
FT   source          1..115
XX
SQ   Sequence 115 BP;
1         LIIEDDPNFA KILFDLAREK GFKGLIAGDG AAGLQ-LAYQ YIPSAILLDI
51        ALPGMDGWMV MEKLKQNPET RHIPVHFISV YA-QSLKAMK MGAVGYLTKP
101       VTLETLDEAF TTIEHAI... .......... .......... ..........
151       .......... .......... .......... .......... ..........
201       .......... .......... .......... ..........
//
ID   00ZZZZ25   ami; 116 BP.
XX
AC   ARB_D44CF0E6;
OS   CBX27662.1 (632-747) uncultured Desulfobacterium sp., enrichment
culture N47
XX
XX
XX
XX
XX
XX
FT   source          1..116
XX
SQ   Sequence 116 BP;
1         LIIEDDSKFA EVLLDLARER GFKGLIAGSG ETGLH-FADY YKPNAVILDI
51        GLPGMDGLSL MERLKENAAT RHIPVHFISA SDDKSIIAMR MGAIGYLTKP
101       VSLEDIDSVF KKIENII... .......... .......... ..........
151       .......... .......... .......... .......... ..........
201       .......... .......... .......... ..........
//
ID   00ZZZZ27   ami; 120 BP.
XX
AC   ARB_7B142757;
OS   EDN68203.1 (358-477) Beggiatoa sp. PS
XX
XX
XX
XX
XX
XX
FT   source          1..120
XX
SQ   Sequence 120 BP;
1         LIIEDDRQFS NLLMELAREK DFKCLLAEKG ETGLQ-LAEQ YHPQAIILDV
51        KLPDIDGWTV IDKLQNNQQT QHIPVHVMSA YD-PNINALD KGAIGYLHKP
```

```
101       ISMPELGEAF KKIEQLITKR VK........ .......... ..........
151       .......... .......... .......... .......... ..........
201       .......... .......... .......... ..........
//
ID   AT3ZZZZZ   ami; 115 BP.
XX
AC   ARB_5E68BEDD;
OS   WP_045434835.1 (816-930) bacterium UASB270, wastewater treatment
sludge
XX
XX
XX
XX
XX
XX
FT   source          1..115
XX
SQ   Sequence 115 BP;
1         LIIEDDPKFA KILFDLARER GFKGLIAGDG SSGLH-LAYQ YVPSAIMLDI
51        NLPELDGRMV MDKLKKNPET RHIPVHFISV LD-ASLEAMK MGAIGYLTKP
101       VSLEQLREAF EKIEDHL... .......... .......... ..........
151       .......... .......... .......... .......... ..........
201       .......... .......... .......... ..........
//
ID   AT4ZZZZZ   ami; 115 BP.
XX
AC   ARB_95A80407;
OS   WP_045569434.1 (981-1095) Vibrio sp. S234-5
XX
XX
XX
XX
XX
XX
FT   source          1..115
XX
SQ   Sequence 115 BP;
1         LIIEDDRAFA GVMRDFGRER GFKCIVAETG ESGLH-FAQY YNPSAIILDI
51        GLPGIDGWTV MERLKENPET RHIPVHFMSA ND-ANLDALR MGAIGYLTKP
101       VDMKKLEKAF GNIEEII... .......... .......... ..........
151       .......... .......... .......... .......... ..........
201       .......... .......... .......... ..........
//
ID   AT5ZZZZ2   ami; 115 BP.
XX
AC   ARB_8418801F;
OS   WP_012141995.1 (990-1104) Shewanella sediminis HAW-EB3
XX
XX
XX
```

```
XX
XX
XX
FT   source          1..115
XX
SQ   Sequence 115 BP;
1         LIIEDDRAFA GVMRDFGRER GFKCIVAETG ETGLH-FADY YKPSAIILDI
51        GLPGIDGWTV MERLKENSEL RHIPVHFMSA SD-NNLDAMR MGAIGYLTKP
101       VDLNKLNKTF GDIEEII... .......... .......... ..........
151       .......... .......... .......... .......... ..........
201       .......... .......... .......... ..........
//
ID   EDDZZZZZ   ami; 115 BP.
XX
AC   ARB_E9C4BC18;
OS   EKD40401.1 (238-352) uncultured bacterium, subsurface aquifer
sediment
XX
XX
XX
XX
XX
XX
FT   source          1..115
XX
SQ   Sequence 115 BP;
1         LIIEDDPSFA GVMRDLARER GFKCILAESG ETGLH-YADY YRPDAIILDI
51        GLPGIDGWEV MARLKENPGL RHIPVHFMSA AD-SAMDALR QGAVGFLAKP
101       VTIEKVEEAF ARIKKII... .......... .......... ..........
151       .......... .......... .......... .......... ..........
201       .......... .......... .......... ..........
//
ID   KJRZZZZ2   ami; 115 BP.
XX
AC   ARB_88EA3D0F;
OS   KJR99450.1 (989-1103) Desulfobulbaceae bacterium BRH_c16a, rock
porewater metagenome
XX
XX
XX
XX
XX
XX
FT   source          1..115
XX
SQ   Sequence 115 BP;
1         LIIEDDPNFA GVMRDFARER GFKCILAESG ETGLH-YADY YRPDAIILDI
51        GLPGIDGWEV MTRLKENPGL RHIPVHFMSA AD-RAMDALR QGAIGFLAKP
101       VTIEKVEEAF NRIKNII... .......... .......... ..........
151       .......... .......... .......... .......... ..........
```

```
201       .......... .......... .......... ..........
//
ID   EAdZZZZZ   ami; 115 BP.
XX
AC   ARB_E1B0087B;
OS   EAT02265.1 (23-137) delta proteobacterium MLMS-1
XX
XX
XX
XX
XX
XX
FT   source          1..115
XX
SQ   Sequence 115 BP;
1         LIIEDDVTFA SLMLDLAREK GYKGLVAEDG ETGLH-LADY YRPSGIILDL
51        GLPGISGLEV MERLKKNPET RHIPVHFVSA SD-RSTELMQ LGAVGFLTKP
101       VSVEKLGEVF DRIEKLV... .......... .......... ..........
151       .......... .......... .......... .......... ..........
201       .......... .......... .......... ..........
//
ID   AT6ZZZZZ   ami; 115 BP.
XX
AC   ARB_DC0E37DC;
OS   WP_051328394.1 (1058-1172) Desulfatirhabdium butyrativorans DSM
18734
XX
XX
XX
XX
XX
XX
FT   source          1..115
XX
SQ   Sequence 115 BP;
1         LIIEDDPHFA RILCDLSHDR GFKVLVADNG ETGLH-FADY YKPDAIVLDV
51        NLPGMDGWTV LERLKANART RHIPVHIISA ND-RPLNALK MGAIGYMTKP
101       ISMDELDKVY TKIEKTI... .......... .......... ..........
151       .......... .......... .......... .......... ..........
201       .......... .......... .......... ..........
//
ID   ASJZZZ11   ami; 115 BP.
XX
AC   ARB_DA211F1;
OS   WP_052491710.1 (798-912) Thioploca ingrica
XX
XX
XX
XX
XX
```

```
XX
FT   source          1..115
XX
SQ   Sequence 115 BP;
1         LIIEDDLTFA YLLMKTAHEK AFKCLVATEG QSGLQ-LAKK YQPQAIILDI
51        GLPKIDGWSI MEQLKADSHT RPIPVHFISG AD-SEQMAKQ RGAIGYCLKP
101       VSMEGLYKVF NNIDHFI... .......... .......... ..........
151       .......... .......... .......... .......... ..........
201       .......... .......... .......... ..........
//
ID   AT7ZZZZZ   ami; 114 BP.
XX
AC   ARB_F61D8F3D;
OS   WP_019467124.1 (833-946) Dyella japonica A8
XX
XX
XX
XX
XX
XX
FT   source          1..114
XX
SQ   Sequence 114 BP;
1         LIIEDDPVFA RILADMVRRK GYRVLAAADG ESGLQ-LATH YRPTGILLDV
51        MLPGMDGWTV IERLKDSPAT RHVPVHFISA VD-EANRGRE LGAVGFLTKP
101       VSREAIGEAF DRLLHF.... .......... .......... ..........
151       .......... .......... .......... .......... ..........
201       .......... .......... .......... ..........
//
ID   AT8ZZZZ2   ami; 120 BP.
XX
AC   ARB_E840671A;
OS   WP_052567468.1 (809-928) Cand. Magnetobacterium casensis
XX
XX
XX
XX
XX
XX
FT   source          1..120
XX
SQ   Sequence 120 BP;
1         LVIEDDPVFA KILYDISHEK GFKCIVAGDG WRGCE-YAET YRPDAIILDV
51        GLPVMDGRMA LERLKDSAGT RNIPVHIVSA ADK-SIDCLK SGVVGYLTKP
101       VSMERLDDVF KKLEGIFAQG SK........ .......... ..........
151       .......... .......... .......... .......... ..........
201       .......... .......... .......... ..........
//
ID   KJUZZZZZ   ami; 114 BP.
XX
```

```
AC   ARB_33E15E5F;
OS   KJU86825.1 (806-919) Cand. Magnetobacterium bavaricum TM-1
XX
XX
XX
XX
XX
XX
FT   source          1..114
XX
SQ   Sequence 114 BP;
1         LVIEDDPVFA RILYNISHEK GFKCIVAGDG QRGCE-YAQT YRPDAIILDV
51        GLPVMDGRMV IEKLKDSAHT RNIPVHIVSA ADK-SVDCLK SGVVGYITKP
101       VSMERLDDVF KKLEAI.... .......... .......... ..........
151       .......... .......... .......... .......... ..........
201       .......... .......... .......... ..........
//
ID   ATEZZZZ2   ami; 115 BP.
XX
AC   ARB_7611E6D9;
OS   WP_013163556.1 (909-1023) Desulfurivibrio alkaliphilus AHT 2
XX
XX
XX
XX
XX
XX
FT   source          1..115
XX
SQ   Sequence 115 BP;
1         LIIEDDSSFA SLLLELARDK GYKGLVAEDG ETGLH-LADY FRPSGIILDM
51        GLPGISGREV MERLKKNPET RHIPVHFISA SD-PSNEVMQ LGAVGFLTKP
101       VSVDKLEQVF GRIEKLA... .......... .......... ..........
151       .......... .......... .......... .......... ..........
201       .......... .......... .......... ..........
//
ID   ATGZZZZZ   ami; 115 BP.
XX
AC   ARB_2551528A;
OS   WP_005009692.1 (876-990) Nitrospina gracilis 3/211
XX
XX
XX
XX
XX
XX
FT   source          1..115
XX
SQ   Sequence 115 BP;
1         LIIEDDLKFA HVLKDMAREK GFKCLIADDG ENGIL-LSQQ FQPSAIILDV
```

```
51        GLPGIDGLMV LEKLKNHPET KPIPVHFISA YE-KDKEAMQ LGAIGFLRKP
101       VTQEKMDEVF SRIEKVL... .......... .......... ..........
151       .......... .......... .......... .......... ..........
201       .......... .......... .......... ..........
//
ID   ATQZZZZZ   ami; 114 BP.
XX
AC   ARB_231BFBF4;
OS   WP_056085375.1 (827-940) Rhodanobacter sp. Root480
XX
XX
XX
XX
XX
XX
FT   source          1..114
XX
SQ   Sequence 114 BP;
1         LVIEDDPAFA RILTDMIHRK GYQVLAAADG ETGLA-LAHE YAPTGILLDV
51        MLPGMDGWTV IERLKADEAT RHLPVHFISA SG-DANRGRE MGAVGFLTKP
101       VSREAIGAAF ERLLHF.... .......... .......... ..........
151       .......... .......... .......... .......... ..........
201       .......... .......... .......... ..........
//
ID   ATXZZZZ2   ami; 114 BP.
XX
AC   ARB_E023D8E1;
OS   WP_048399379.1 (580-693) Cand. Achromatium palustre
XX
XX
XX
XX
XX
XX
FT   source          1..114
XX
SQ   Sequence 114 BP;
1         LVIEDDAAFA RIVRDLARNR GFKCLLANDG LLGIQ-MARL YRPIGIVLDV
51        NLPSIDGWNV MQQLKKHPET SHIPVHFISA ND-FSQRGLE MGAIGYLTKP
101       VTKEQIEGVF EKLHQV.... .......... .......... ..........
151       .......... .......... .......... .......... ..........
201       .......... .......... .......... ..........
//
ID   ATYZZZZZ   ami; 114 BP.
XX
AC   ARB_E5F2D02B;
OS   WP_046972715.1 (832-945) Dyella japonica DSM 16301
XX
XX
XX
```

```
XX
XX
XX
FT   source          1..114
XX
SQ   Sequence 114 BP;
1         LIIEDDPVFA RILADMVRRK GYRVLAAADG ESGLQ-LAAK HSPTGILLDV
51        MLPGMDGWTV IERLKENAAT RHVPVHFISA VD-EANRGRD LGAVGFLTKP
101       VSREAIGEAF DRLLHF.... .......... .......... ..........
151       .......... .......... .......... .......... ..........
201       .......... .......... .......... ..........
//
ID   AU0ZZZZZ   ami; 115 BP.
XX
AC   ARB_A819E8DB;
OS   WP_039463312.1 (981-1095) Vibrio navarrensis 0053-83
XX
XX
XX
XX
XX
XX
FT   source          1..115
XX
SQ   Sequence 115 BP;
1         LIIEDDRAFA GVMRDFGRER GFKCIVAETG ESGLH-FAQY YNPSAIILDI
51        GLPGIDGWTV MERLKENPDT RHIPVHFMSA ND-ANLDALR MGAIGYLTKP
101       VDMKKLEKAF GNIEEII... .......... .......... ..........
151       .......... .......... .......... .......... ..........
201       .......... .......... .......... ..........
//
ID   WjZZZZZ3   ami; 114 BP.
XX
AC   ARB_A0A23C88;
OS   WP_038615294.1 (833-946) Dyella jiangningensis SBZ_3-12
XX
XX
XX
XX
XX
XX
FT   source          1..114
XX
SQ   Sequence 114 BP;
1         LIVEDDPVFA RILADMVRRK GYRVLVAMDG ESGLQ-LAAR HVPTGILLDV
51        MLPGMDGWTV IERLKENPAT RHVPVHFISA VD-EASRGRD LGAVGFLTKP
101       VSREAIGSAF ERLLHF.... .......... .......... ..........
151       .......... .......... .......... .......... ..........
201       .......... .......... .......... ..........
//
```

```
ID   AU3WP200   ami; 113 BP.
XX
AC   ARB_50988610;
OS   WP_026635597.1 (830-942) Dyella japonica UNC79MFTsu3.2
XX
XX
XX
XX
XX
XX
FT   source          1..113
XX
SQ   Sequence 113 BP;
1         LIVEDDPVFA RILADMVRRK GYRALAAADG ESGLQ-LAQR FRPTGILLDV
51        MLPGMDGWTV IARLKENAAT RHIPVHFISA VD-DASRGRD LGAVGFLTKP
101       VSRESIDGAF DRLLH..... .......... .......... ..........
151       .......... .......... .......... .......... ..........
201       .......... .......... .......... ..........
//
ID   AU4ZZZZZ   ami; 115 BP.
XX
AC   ARB_314BC434;
OS   WP_018085118.1 (1028-1142) Desulfurispora thermophila DSM 16022
XX
XX
XX
XX
XX
XX
FT   source          1..115
XX
SQ   Sequence 115 BP;
1         LIIEDDRNFA GLLADLARQK GFQTAWAESG EKGIA-LARQ LQPAAIILDI
51        GLPGIDGWKV LEILKGDQLT RHIPVHFISA MD-HSLEAMR RGAIGYLTKP
101       VSPEDLDQVF QKIQDII... .......... .......... ..........
151       .......... .......... .......... .......... ..........
201       .......... .......... .......... ..........
//
ID   AU5ZZZZZ   ami; 115 BP.
XX
AC   ARB_121416A7;
OS   WP_012805237.1 (970-1084) Desulfomicrobium baculatum DSM 4028
XX
XX
XX
XX
XX
XX
FT   source          1..115
XX
```

```
SQ   Sequence 115 BP;
1         LIIEDDQHFA RILRDQAREM GFKALLAADG ETGLH-FADF HGPSAIILDN
51        ILPGMNGWTV MERLKNDPKT RHIPLSFISA ED-RSLEAMR MGALAFLTKP
101       VELETLRTLL ERIDSFI... .......... .......... ..........
151       .......... .......... .......... .......... ..........
201       .......... .......... .......... ..........
//
ID   AUEZZZZZ   ami; 115 BP.
XX
AC   ARB_2194EC5F;
OS   WP_014956971.1 (991-1105) Desulfobacula toluolica Tol2
XX
XX
XX
XX
XX
XX
FT   source          1..115
XX
SQ   Sequence 115 BP;
1         LIIEDDNEFS AILAAFFQKN GYESIIASDG ETGIK-FVIE HQPTAIILDI
51        GLPGIDGWAV LSELKNNPAT RHIPVHIMSA FD-NTRQGLE KGAVGYLTKP
101       VDTRGLQKAL NRIESVL... .......... .......... ..........
151       .......... .......... .......... .......... ..........
201       .......... .......... .......... ..........
//
ID   AUFZZZZZ   ami; 115 BP.
XX
AC   ARB_A529B33C;
OS   WP_035223370.1 (991-1105) Desulfobacula sp. TS
XX
XX
XX
XX
XX
XX
FT   source          1..115
XX
SQ   Sequence 115 BP;
1         LIIEDDNEFS AILAAFFQKN GYESIIASDG ETGIK-FVIE HQPTAIILDI
51        GLPGIDGWAV LSELKNNPAT RHIPVHIMSA FD-NTRQGLE KGAVGYLTKP
101       VDTKGLKKAL NRIESVL... .......... .......... ..........
151       .......... .......... .......... .......... ..........
201       .......... .......... .......... ..........
//
ID   AXCZZZZZ   ami; 115 BP.
XX
AC   ARB_14C8A565;
OS   AGX87357.1 (995-1109) Candidatus Symbiobacter mobilis CR
XX
```

```
XX
XX
XX
XX
XX
FT   source          1..115
XX
SQ   Sequence 115 BP;
1         LIVEDDIRFA EILADTAHDR GFKVLLATDG ETGLY-LADY HQPSGIILDI
51        GLPGISGLKV MERLKESSRT RHIPVHIMSA AD-RQIDVLN NGAVGFLVKP
101       VEIEAMHTAF ARIEHII... .......... .......... ..........
151       .......... .......... .......... .......... ..........
201       .......... .......... .......... ..........
//
ID   KWTZZZZZ   ami; 115 BP.
XX
AC   ARB_ACEAF27C;
OS   KWT91955.1 (805-919) Nitrospirae bacterium HCH-1
XX
XX
XX
XX
XX
XX
FT   source          1..115
XX
SQ   Sequence 115 BP;
1         LIIEDDTAFA KILLDISRER GYKSIAVQNG TDGLE-AAHI YKPDAIILDM
51        GLPDMDGWTV IERLKESSET RSIPVQIISA SEK-NIDVLK SGSIGYLTKP
101       VNMEKIEEAF LKIETIF... .......... .......... ..........
151       .......... .......... .......... .......... ..........
201       .......... .......... .......... ..........
//
ID   AUM91432   ami; 114 BP.
XX
AC   ARB_2846F4C6;
OS   WP_036110931.1 (832-945) Luteibacter spp. 9143, 9145
XX
XX
XX
XX
XX
XX
FT   source          1..114
XX
SQ   Sequence 114 BP;
1         LVVEDDPAFA RILADMIHRK GHRVLAAADG ETGLA-LANR YRPTGILLDV
51        MLPGMDGWTV IERLKGDAAT RHIPVHFISA TD-DSTRGLE LGAVGFLTKP
101       VSRESISDAF ERLLHF.... .......... .......... ..........
151       .......... .......... .......... .......... ..........
```

```
201       .......... .......... .......... ..........
//
ID   AUQZZZZ2   ami; 114 BP.
XX
AC   ARB_2C870410;
OS   WP_045831095.1 (833-946) Luteibacter yeojuensis SU11
XX
XX
XX
XX
XX
XX
FT   source          1..114
XX
SQ   Sequence 114 BP;
1         LVVEDDPAFA RILADMIHRK GHRVLAAADG ETGLA-LARQ YRPTGILLDV
51        MLPGMDGWTV IDRLKNDANT RHIPVHFISA TD-DATRGLE LGAVGFLTKP
101       VSRESIATAF ERLLHF.... .......... .......... ..........
151       .......... .......... .......... .......... ..........
201       .......... .......... .......... ..........
//
ID   EPYYyyyy   ami; 111 BP.
XX
AC   ARB_59FDF794;
OS   WP_021132364.1 (787-897) gacS Phaeospirillum fulvum MGU-K5
XX
XX
XX
XX
XX
XX
FT   source          1..111
XX
SQ   Sequence 111 BP;
1         LIIEDDESFS LILSDLARSR GFKCVRVGDG VEGLG-LARQ LLPTGILLDI
51        GLPGLDGWAV MEALKHDPLT RPIPVHFISA TN-DTLRGLR MGAAGFLTKP
101       VDRDQLNDVF DRL....... .......... .......... ..........
151       .......... .......... .......... .......... ..........
201       .......... .......... .......... ..........
//
ID   AV0ZZZZZ   ami; 115 BP.
XX
AC   ARB_AE386C76;
OS   WP_028585323.1 (1008-1122) Desulfobulbus mediterraneus DSM 13871
XX
XX
XX
XX
XX
XX
```

FT   source   1..115
XX
SQ   Sequence 115 BP;
1   LIIEDDKVFA TILSNYFQKN GYQSIVADSG ESGVK-YAIE HKPTAVILDI
51   GLPGIDGWTV LNELKKNPDT RHIPVHIMSA FD-ASGKGLQ QGAVGYLTKP
101   VGSQELRTAL ESIEYVL... ....
151   ....
201   ....
//
ID   AV4ZZZZ2   ami; 114 BP.
XX
AC   ARB_7002DED7;
OS   WP_046965975.1 (832-945) Luteibacter rhizovicinus DSM 16549
XX
XX
XX
XX
XX
XX
FT   source   1..114
XX
SQ   Sequence 114 BP;
1   LVIEDDPAFA RILADMVHRK GHRVLAAGDG ESGLA-LAKR YRPTGILLDV
51   MLPGMDGWTV IERLKGDVAT RHIPVHFISA TD-DATRGME LGAVGFLTKP
101   VSRESIANAF DRMLHF.... ....
151   ....
201   ....
//
ID   AUNZZZZ3   ami; 114 BP.
XX
AC   ARB_249B429F;
OS   WP_036137243.1 (832-945) Luteibacter sp. 9135
XX
XX
XX
XX
XX
XX
FT   source   1..114
XX
SQ   Sequence 114 BP;
1   LVVEDDPAFA RILADMIHRK GHRVLAAADG ETGLA-LAKQ YRPTGILLDV
51   MLPGMDGWTV IERLKGDVAT RHIPVHFISA TD-DASRGLE LGAVGFLTKP
101   VSRESIGDAF DRLLHF.... ....
151   ....
201   ....
//
ID   AV5ZZZZZ   ami; 111 BP.
XX
AC   ARB_89E01B39;

```
OS   WP_056001001.1 (834-944) Frateuria sp. Soil773
XX
XX
XX
XX
XX
XX
FT   source          1..111
XX
SQ   Sequence 111 BP;
1         LIVEDDPAFA RILADMVRQK GHRVLLAANG ENGLA-LARR YRPLGILLDV
51        MLPGIDGWAV IDRLKSDPAT RHIPVHFLSA ND-DAGRGRE LGAVGFLTKP
101       VSREAIDEAF ERL....... .......... .......... ..........
151       .......... .......... .......... .......... ..........
201       .......... .......... .......... ..........
//
ID   AV7ZZZZZ   ami; 114 BP.
XX
AC   ARB_CB8CEC51;
OS   WP_051361761.1 (843-956) Solimonas soli DSM 21787
XX
XX
XX
XX
XX
XX
FT   source          1..114
XX
SQ   Sequence 114 BP;
1         LIVEDDAAFA RVLADITRRK GYRVLLAGDG ESGLE-LARR YRPNGILLDV
51        DLPGMNGWGV MAALKADGAT RHIPVHFISA TD-ESSRGMA MGAVGFLTKP
101       VTREGLDSVF QRVLQA.... .......... .......... ..........
151       .......... .......... .......... .......... ..........
201       .......... .......... .......... ..........
//
ID   AVAZZZZZ   ami; 115 BP.
XX
AC   ARB_168FDDF2;
OS   WP_025294632.1 (840-954) Sphingomonas sanxanigenens DSM 19645
XX
XX
XX
XX
XX
XX
FT   source          1..115
XX
SQ   Sequence 115 BP;
1         LIVEDDLRFA KIMLDVVRSK NYAGLVAGDG ESGIA-LARR YKPRGILLDA
51        MLPGMDGWTV IERLKEDDGT RHIPVHFISA TD-EAPRARA LGAIGFLTKP
```

```
101       VTREDIADVF GRFEHYA... .......... .......... ..........
151       .......... .......... .......... .......... ..........
201       .......... .......... .......... ..........
//
ID   AWBZZZZZ   ami; 80 BP.
XX
AC   ARB_CC21F777;
OS   518251480_WP_019421688_1_879-958_Paenibacillus_sp._OSY-SE
XX
XX
XX
XX
XX
XX
FT   source          1..80
XX
SQ   Sequence 80 BP;
1         LIVEDDGPQR QSLIALIEGV DVSVTAVSTG TEALKQLADR K-FDGMVLDL
51        LLPDMTGFEL MDHISRNPQL RRVPIIVYTG K--------- ----------
101       ---------- ---------- ---------- ---------- ----------
151       ---------- ---------- ---------- ---------- ----------
201       ---------- ---------- ---------- ----------
//
ID   AU0ZZZZ5   ami; 202 BP.
XX
AC   ARB_96DEF2F0;
OS   WP_039442982.1 (981-1182) Vibrio navarrensis 08-2462
XX
XX
XX
XX
XX
XX
FT   source          1..202
XX
SQ   Sequence 202 BP;
1         LIIEDDRAFA GVMRDFGRER GFKCIVAETG ESGLH-FAQY YNPSAIILDI
51        GLPGIDGWTV MERLKENPET RHIPVHFMSA ND-ANLDALR MGAIGYLTKP
101       VDMKKLEKAF GNIEEIISKP VKRLLVVEDD AIQQESIRQL IGEDDVHIVT
151       VPTGEKALSE LESARYDCMV LDLGLEDMTG FELLERIRRS ETAARVPIIV
201       YTGR------ ---------- ---------- ----------
//
ID   ASYZZZZ2   ami; 238 BP.
XX
AC   ARB_A890AFE1;
OS   WP_017422776.1 (986-1223) Vibrio vulnificus ATCC 27562
XX
XX
XX
XX
```

```
XX
XX
FT   source          1..238
XX
SQ   Sequence 238 BP;
1         LIIEDDRAFA SVMRDFGRER GFKCIVAETG ETGLH-FAQY YKPSAIILDI
51        GLPGIDGWTV MERLKENPET RHIPVHFMSA ND-ANLDALR MGAIGYLTKP
101       VDMKKLEKAF GNIEDILSKP VKRLLVVEDD AIQQESIRQL IGENDIHIVA
151       VPTGEKALEE LESNRYDCMV LDLGLEDMTG FELLERIRRS ETAARVPIIV
201       YTGRELSKEE ERELNRYAES IIIKGVKSPE RLLDESALFL
//
ID   ASYZZZZ8   ami; 210 BP.
XX
AC   ARB_7B93BF8C;
OS   WP_045609177.1 (989-1195) Vibrio_vulnificus SC9740
XX
XX
XX
XX
XX
XX
FT   source          1..210
XX
SQ   Sequence 210 BP;
1         LIIEDDRAFA SVMRDFGRER GFKCIVAETG ETGLH-FAQY YKPSAIILDI
51        GLPGIDGWTV MERLKENPET RHIPVHFMSA ND-ANLDALR MGAIGYLTKP
101       VDMKKLEKAF GNIEDILSKP VKRLLVVEDD AIQQESIRQL IGEDDIHIVA
151       VPTGEKALEE LESNRYDCMV LDLGLEDMTG FELLERIRRS ETAARVPIIV
201       YTGRELSKEE ER-------- ---------- ---------.
//
ID   AUYZZZZ2   ami; 82 BP.
XX
AC   ARB_D901CEC2;
OS   655083960_WP_028532005_1_868-949_Paenibacillus_sp._UNC217MF
XX
XX
XX
XX
XX
XX
FT   source          1..82
XX
SQ   Sequence 82 BP;
1         LIVEDDGPQR QSLIALIEGA DVAVTAVSTG TEALKVLGEE N-FDGMVLDL
51        LLPDMTGFEL MDEISHHSRI RRIPIIVYTG KLL------- ----------
101       ---------- ---------- ---------- ---------- ----------
151       ---------- ---------- ---------- ---------- ----------
201       ---------- ---------- ---------- ----------
//
ID   AVWZZZZ4   ami; 80 BP.
```

```
XX
AC   ARB_83EAF85A;
OS   544846345_WP_021261593_1_868-947_Paenibacillus_alvei
XX
XX
XX
XX
XX
XX
FT   source          1..80
XX
SQ   Sequence 80 BP;
1         LIVEDDGPQR QSLIALIEGA DVAVTAVSTG TEALKVLGEE K-FDGMVLDL
51        LLPDMTGFEL MDEISHHSRI RRIPIIVYTG K--------- ----------
101       ---------- ---------- ---------- ---------- ----------
151       ---------- ---------- ---------- ---------- ----------
201       ---------- ---------- ---------- ----------
//
```
