# Supplementary material for: Visualizing Evolutionary Relationships of Multidomain Proteins: An Example from Receiver (REC) Domains of Sensor Histidine Kinases in the Candidatus Maribeggiatoa str. Orange Guaymas Draft Genome
Source: Front Microbiol. 2016 Nov 14;7:1780. doi: 10.3389/fmicb.2016.01780 (PMC5108060; doi:10.3389/fmicb.2016.01780)
Supplement: Supplementary file 8 [file DataSheet6.PDF]

```
ID   Yp100000   ami; 117 BP.
XX
AC   ARB_638E959A;
OS   YP_001036718.1 (9-125)
response_regulator_receiver_sensor_signal_transduction_histidine_kinas
e_Ruminiclostridium thermocellum ATCC 27405
XX
XX
XX
XX
XX
XX
FT   source          1..117
XX
SQ   Sequence 117 BP;
1         .......... .......... .......... .......... ..........
51        .......... ...LIVDDNE NNLFSLRTL- -IE------- EH---IN---
101       --AD------ --VKEAN--S GEKALKI--- ---------- ----------
151       -------L-- ---------- F----KE--- R--------- ------V---
201       -----D---- --L-IILDIQ M-E--G-M-- ---DG--FEL AS---I--I-
251       --K----K-- ---------- ---------- ---------- ----------
301       ---------- ---------- ------R--- ---K-----K ------TS--
351       S--------- ---I--P--- ----I--VFL T---ASY--- ---IGDEFQR
401       RGFEIGA--- ---VDYL-TK PIDEYQL--- ---------- INRINVYL..
451       .......... .......... .......... .......... ..........
501       ......
//
ID   Wp100000   ami; 117 BP.
XX
AC   ARB_6EF5679A;
OS   WP_017414985.1 (10-126) hypothetical_protein_Clostridium
tunisiense TJ
XX
XX
XX
XX
XX
XX
FT   source          1..117
XX
SQ   Sequence 117 BP;
1         .......... .......... .......... .......... ..........
51        .......... ...LIVDDNH NNLFTLREL- -IK------- EY---ID---
101       --AE------ --ILEAD--C GEQALTI--- ---------- ----------
151       -------L-- ---------- Y----DR--- A--------- ------V---
201       -----D---- --L-IILDIQ M-E--G-M-- ---DG--FEI AS---L--I-
251       --K----N-- ---------- ---------- ---------- ----------
301       ---------- ---------- ------R--- ---K-----K ------TK--
351       D--------- ---I--P--- ----I--VFL T---AAY--- ---KNDEFRS
401       RGFELGA--- ---VDYL-VK PIDEYQL--- ---------- INRINVYL..
```

```
451       .......... .......... .......... .......... ..........
501       ......
//
ID   Eqb10000   ami; 117 BP.
XX
AC   ARB_66445B69;
OS   EQB89702.1 (10-126) hypothetical_protein_M918_19540_Clostridium
sp. BL8
XX
XX
XX
XX
XX
XX
FT   source          1..117
XX
SQ   Sequence 117 BP;
1         .......... .......... .......... .......... ..........
51        .......... ...LIVDDNH SNLFTLREF- -IK------- EY---ID---
101       --AE------ --ILEAD--C GEDALTL--- ---------- ----------
151       -------L-- ---------- Y----DK--- T--------- ------V---
201       -----D---- --L-IILDVQ M-E--G-M-- ---DG--FEI AS---L--I-
251       --K----K-- ---------- ---------- ---------- ----------
301       ---------- ---------- ------R--- ---K-----K ------TK--
351       D--------- ---I--P--- ----I--VFL T---AAY--- ---KNDEFKK
401       RGFEIGA--- ---VDYL-VK PIDEFQL--- ---------- INRINVYL..
451       .......... .......... .......... .......... ..........
501       ......
//
ID   WPreYyyy   ami; 118 BP.
XX
AC   ARB_11611640;
OS
WP_003444572_1_10_127_response_regulator_receiver_sensor_signal_transd
uction_histidine_kinase_Clostridium_pasteurianum_DSM 525
XX
XX
XX
XX
XX
XX
FT   source          1..118
XX
SQ   Sequence 118 BP;
1         .......... .......... .......... .......... ..........
51        .......... ...LVVDDNQ NNLFTLKTL- -IK------- EY---ID---
101       --AN------ --VLIAD--T PKKALQE--- ---------- ----------
151       -------L-- ---------- I----KN--- S--------- ------V---
201       -----D---- --L-IILDVH MKR--E-M-- ---DG--FEL AQ---I--I-
251       --K----G-- ---------- ---------- ---------- ----------
```

301   ---------- ---------- ------R--- ---K-----K ------TE--
351   H--------- ---I--P--- ----I--VFL T---GAY--- ---ISEEFKK
401   KGFEIGA--- ---VDYL-TK PIDDFLL--- ---------- INRINVYL..
451   ....
501   ......
//
ID   WPnkYyyy   ami; 118 BP.
XX
AC   ARB_4B431475;
OS   WP_010233239_1_10_127_histidine_kinase_Clostridium_arbusti_SL206
XX
XX
XX
XX
XX
XX
FT   source   1..118
XX
SQ   Sequence 118 BP;
1   ....
51   ....LVVDDDQ NNLFTVKTL- -IK------- EY---ID---
101   --AN------ --VLTAD--T PKKALQE--- ---------- ----------
151   -------L-- ---------- I----KN--- P--------- ------V---
201   -----D---- --L-IILDVQ MKR--E-M-- ---DG--FEL AQ---I--I-
251   --K----G-- ---------- ---------- ---------- ----------
301   ---------- ---------- ------R--- ---K-----K ------TE--
351   H--------- ---I--P--- ----I--VFL T---GAY--- ---IGDDFKK
401   RGFEIGA--- ---VDYL-TK PIDDFLL--- ---------- INRINVYL..
451   ....
501   ......
//
ID   YPsiYyyy   ami; 118 BP.
XX
AC   ARB_244A71C7;
OS
YP_007942684_1_10_127_signal_transduction_histidine_kinase_Clostridium
_pasteurianum_BC1_
XX
XX
XX
XX
XX
XX
FT   source   1..118
XX
SQ   Sequence 118 BP;
1   ....
51   ....LVVDDDE NNLFTVKTL- -IK------- EY---ID---
101   --AN------ --VLIAD--T PKKALQE--- ---------- ----------
151   -------L-- ---------- I----KN--- P--------- ------I---

```
201       -----D---- --L-IILDVQ MKR--E-M-- ---DG--FEL AQ---I--I-
251       --K----G-- ---------- ---------- ---------- ----------
301       ---------- ---------- ------R--- ---K-----K ------TE--
351       H--------- ---I--P--- ----I--VFL T---GAY--- ---IGEDFKK
401       RGFEIGA--- ---VDYL-TK PIDDFLL--- ---------- INRINVYL..
451       .......... .......... .......... .......... ..........
501       ......
//
ID   BOUYyyyy   ami; 116 BP.
XX
AC   ARB_313C0501;
OS
BOGUAY_2502843810_01308_0467_707_822_ATPase,_histidine_kinase-,_DNA_gy
rase_B-,_and_HSP90-like_domain_protein
XX
XX
XX
XX
XX
XX
FT   source          1..116
XX
SQ   Sequence 116 BP;
1         .......... .......... .......... .......... ..........
51        .......... ...LVVDDEL VNLQVLINH- -LT------- L----QN---
101       --YA------ --ISRAT--N GIDALAE--- ---------- ----------
151       -------I-- ---------- E----KGY-- K--------- ------P---
201       -----D---- --L-VLLDVM M-P--K-M-- ---TG--YEV CR---K--I-
251       --R----E-- ---------- ---------- ---------- ----------
301       ---------- ---------- ------Q--- ---F-----P ------LN--
351       E--------- ---L--P--- ----V--LML T---AKN--- ----QVSDLV
401       EGLESGA--- ---NDYL-TK PISKHEL--- ---------- IARIKIHL..
451       .......... .......... .......... .......... ..........
501       ......
//
ID   BOUAYyy3   ami; 117 BP.
XX
AC   ARB_60DB5D4F;
OS
BOGUAY_2502842938_01054_0100_681_797_ATPase,_histidine_kinase-,_DNA_gy
rase_B-,_and_HSP90-like_domain_protein_
XX
XX
XX
XX
XX
XX
FT   source          1..117
XX
SQ   Sequence 117 BP;
```

```
1         .......... .......... .......... .......... ..........
51        .......... ...LIVDDDP INLQVLENQ- -LS------- L----EN---
101       --YA------ --ISHAH--N GQEALDA--- ---------- ----------
151       -------I-- ---------- N----SGIT- T--------- ------Y---
201       -----A---- --L-ILLDIM M-P--K-M-- ---SG--FEV CK---I--I-
251       --R----Q-- ---------- ---------- ---------- ----------
301       ---------- ---------- ------T--- ---H-----P ------AN--
351       L--------- ---L--P--- ----I--IML T---AKN--- ----QVSDLV
401       EGLQAGA--- ---NDYL-TK PFSKGEL--- ---------- IARLKMHI..
451       .......... .......... .......... .......... ..........
501       ......
//
ID   BOGUYyy5   ami; 116 BP.
XX
AC   ARB_5E744DB;
OS   BOGUAY_2502840933_00470_1295_187_302_PAS_domain_S-box_
XX
XX
XX
XX
XX
XX
FT   source          1..116
XX
SQ   Sequence 116 BP;
1         .......... .......... .......... .......... ..........
51        .......... ...LVVDDDN INRRVLVSM- -LS------- L----KN---
101       --YT------ --TIEAS--S GIETLTL--- ---------- ----------
151       -------I-- ---------- E----NGS-- K--------- ------P---
201       -----D---- --I-ILMDVM M-P--D-L-- ---TG--LEV TR---K--L-
251       --R----E-- ---------- ---------- ---------- ----------
301       ---------- ---------- ------T--- ---W-----Q ------AD--
351       E--------- ---L--P--- ----I--LLV T---ANT--- ----QVANIV
401       EGLESGA--- ---NDYM-KK PVFKDEL--- ---------- LAKVKTHL..
451       .......... .......... .......... .......... ..........
501       ......
//
ID   BOEFYyyy   ami; 117 BP.
XX
AC   ARB_E68E040;
OS   BOGUAY_2502840240_00316_2961_1148_1264_GGDEF_domain_protein_
XX
XX
XX
XX
XX
XX
FT   source          1..117
XX
SQ   Sequence 117 BP;
```

```
1         .......... .......... .......... .......... ..........
51        .......... ...LIVDDDL VNRQVLLNQ- -LS------- L---EKN---
101       --YY------ --LNQAA--S GLETLEF--- ---------- ----------
151       -------L-- ---------- E----QGY-- Q--------- ------P---
201       -----D---- --L-ILLDVM M-P--H-L-- ---TG--YEV TQ---K--I-
251       --R----E-- ---------- ---------- ---------- ----------
301       ---------- ---------- ------T--- ---K-----T ------PD--
351       E--------- ---L--P--- ----I--ILL T---AKN--- ----QVTDLV
401       TGLESGA--- ---NDYL-TK PVSKYEL--- ---------- LARIRTHL..
451       .......... .......... .......... .......... ..........
501       ......
//
ID   BOtYyyyy   ami; 117 BP.
XX
AC   ARB_6C9E41E9;
OS
BOGUAY_2502840176_00301_1145_1112_1228_Stage_II_sporulation_protein_E_
XX
XX
XX
XX
XX
XX
FT   source          1..117
XX
SQ   Sequence 117 BP;
1         .......... .......... .......... .......... ..........
51        .......... ...FIVDDDA VNRQVLINH- -LS------- L---QQN---
101       --YA------ --LTQAS--S GVEALAL--- ---------- ----------
151       -------M-- ---------- K----EGY-- K--------- ------P---
201       -----D---- --L-ILLDVM M-P--H-I-- ---SG--YEV TE---E--I-
251       --R----Q-- ---------- ---------- ---------- ----------
301       ---------- ---------- ------T--- ---Q-----Q ------AN--
351       E--------- ---L--P--- ----I--LLL T---AKN--- ----QTSDLV
401       TGLEVGA--- ---NDYL-TK PVFKEEL--- ---------- LARIKTHL..
451       .......... .......... .......... .......... ..........
501       ......
//
ID   BOUAYY11   ami; 116 BP.
XX
AC   ARB_4B79E9D9;
OS
BOGUAY_2502839728_00217_2058_747_862_ATPase,_histidine_kinase-,_DNA_gy
rase_B-,_and_HSP90-like_domain_protein_
XX
XX
XX
XX
XX
XX
```

```
FT   source          1..116
XX
SQ   Sequence 116 BP;
1         .......... .......... .......... .......... ..........
51        .......... ...LIVDDEA MNLHVLTNY- -LS------- P----YH---
101       --YH------ --IIQAA--S GFQALKI--- ---------- ----------
151       -------L-- ---------- E----KGE-- I--------- ------P---
201       -----D---- --L-ILLDVM M-P--Y-M-- ---TG--YEV TQ---K--I-
251       --R----K-- ---------- ---------- ---------- ----------
301       ---------- ---------- ------R--- ---W-----E ------AN--
351       E--------- ---L--P--- ----I--ILI T---AKN--- ----QVADLV
401       MGLEVGA--- ---NDYL-VK PTSKNEL--- ---------- IARMKTHL..
451       .......... .......... .......... .......... ..........
501       ......
//
ID   YurYyyyy   ami; 116 BP.
XX
AC   ARB_27F9D15E;
OS
YP_720573_1_725_840_adenylate_guanylate_cyclase_Trichodesmium_erythrae
um_IMS101_
XX
XX
XX
XX
XX
XX
FT   source          1..116
XX
SQ   Sequence 116 BP;
1         .......... .......... .......... .......... ..........
51        .......... ...LVVDDEP INIQVLNNY- -LK------- A----NN---
101       --YQ------ --VTQAL--S GKEALAA--- ---------- ----------
151       -------L-- ---------- E----NNH-- N--------- ------F---
201       -----D---- --L-ILLDVM M-P--N-M-- ---SG--YEV CS---Q--I-
251       --R----E-- ---------- ---------- ---------- ----------
301       ---------- ---------- ------K--- ---Y-----P ------AQ--
351       S--------- ---L--P--- ----V--LML T---AKN--- ----QIADLV
401       MGFQFGA--- ---NDYL-TK PFAKDEL--- ---------- LTRIQTHI..
451       .......... .......... .......... .......... ..........
501       ......
//
ID   YuriYyyy   ami; 116 BP.
XX
AC   ARB_91C7DA23;
OS
YP_720574_1_722_837_adenylate_guanylate_cyclase_Trichodesmium_erythrae
um_IMS101_
XX
XX
```

```
XX
XX
XX
XX
FT   source          1..116
XX
SQ   Sequence 116 BP;
1          .......... .......... .......... .......... ..........
51         .......... ...LVVDDEP INIQVLNNY- -LK------- A----NN---
101        --YQ------ --VTQAL--S GKEALAA--- ---------- ----------
151        -------L-- ---------- E----NNH-- N--------- ------F---
201        -----D---- --L-ILLDIM M-P--N-M-- ---TG--YEV CS---Q--I-
251        --R----E-- ---------- ---------- ---------- ----------
301        ---------- ---------- ------K--- ---Y-----P ------AQ--
351        S--------- ---L--P--- ----V--LML T---AKN--- ----QIADLV
401        MGFQFGA--- ---NDYL-TK PFAKDEL--- ---------- LTRIQTHI..
451        .......... .......... .......... .......... ..........
501        ......
//
ID   YuriYyy2   ami; 116 BP.
XX
AC   ARB_BFB44C92;
OS
YP_722985_1_742_857_adenylate_guanylate_cyclase_Trichodesmium_erythrae
um_IMS101_
XX
XX
XX
XX
XX
XX
FT   source          1..116
XX
SQ   Sequence 116 BP;
1          .......... .......... .......... .......... ..........
51         .......... ...LIVDDEP VNLTLLSNQ- -LS------- L----HN---
101        --YQ------ --VIQAN--N GQKALDI--- ---------- ----------
151        -------L-- ---------- A----QDF-- L--------- ------P---
201        -----D---- --L-ILLDVM M-P--G-M-- ---TG--YEV TQ---K--V-
251        --R----E-- ---------- ---------- ---------- ----------
301        ---------- ---------- ------T--- ---W-----L ------LH--
351        Q--------- ---L--P--- ----I--IML T---AKN--- ----RVSDLV
401        MGLEVGA--- ---NDYL-SK PFHKKEL--- ---------- LARIKTHI..
451        .......... .......... .......... .......... ..........
501        ......
//
ID   ACVYyyyy   ami; 116 BP.
XX
AC   ARB_CD985A15;
OS
```

```
ACV42481_1_748_863_putative_adenylate_guanylate_cyclase_Moorea_produce
ns_(Lyngbya_majuscula)_19L_
XX
XX
XX
XX
XX
XX
FT   source          1..116
XX
SQ   Sequence 116 BP;
1          .......... .......... .......... .......... ..........
51         .......... ...LIVDDEP VNRQVISNY- -LS------- M----EN---
101        --YA------ --IATAT--N GLEALEM--- ---------- ----------
151        -------L-- ---------- S----TGF-- Q--------- ------P---
201        -----D---- --L-ILLDVM M-P--R-M-- ---TG--YEV CA---K--I-
251        --R----Q-- ---------- ---------- ---------- ----------
301        ---------- ---------- ------K--- ---F-----L ------PS--
351        E--------- ---L--P--- ----V--IML T---ANN--- ----QISDLV
401        EGFTFGV--- ---NDYL-TK PCSKHEL--- ---------- LARIKSHL..
451        .......... .......... .......... .......... ..........
501        ......
//
ID   Yg0uyyyy   ami; 116 BP.
XX
AC   ARB_B3E4DE2C;
OS
YP_004446809_1_1201_1316_guanylate_cyclase_Haliscomenobacter_hydrossis
_DSM_1100_
XX
XX
XX
XX
XX
XX
FT   source          1..116
XX
SQ   Sequence 116 BP;
1          .......... .......... .......... .......... ..........
51         .......... ...LIVDDEV VNQQVLKNF- -LN------- K----AI---
101        --YR------ --ITQVL--S GEEALQV--- ---------- ----------
151        -------L-- ---------- D----DDD-- T--------- ------I---
201        -----D---- --L-VLLDVM M-P--R-M-- ---SG--YEV CQ---K--I-
251        --R----E-- ---------- ---------- ---------- ----------
301        ---------- ---------- ------K--- ---H-----L ------PS--
351        E--------- ---L--P--- ----V--LMI T---AKN--- ----QVSDLV
401        TGLNTGA--- ---NDYI-AK PFSKDEF--- ---------- LARLNMHL..
451        .......... .......... .......... .......... ..........
501        ......
//
```

```
ID   Yg0uayyy   ami; 116 BP.
XX
AC   ARB_FC98F965;
OS
YP_004447710_1_1194_1309_guanylate_cyclase_Haliscomenobacter_hydrossis
_DSM_1100_
XX
XX
XX
XX
XX
XX
FT   source          1..116
XX
SQ   Sequence 116 BP;
1         .......... .......... .......... .......... ..........
51        .......... ...LVVDDEP INQQVLKSH- -LS------- A----LK---
101       --YD------ --ITSAL--N GEDALKA--- ---------- ----------
151       -------L-- ---------- S----NGK-- H--------- ------F---
201       -----D---- --L-VLLDVM M-P--R-M-- ---SG--YEV CE---Q--I-
251       --R----K-- ---------- ---------- ---------- ----------
301       ---------- ---------- ------K--- ---F-----L ------PS--
351       E--------- ---L--P--- ----V--IMI T---AKN--- ----QVQDLV
401       HGLNTGA--- ---NDYI-TK PFTKDEF--- ---------- LARVKTHL..
451       .......... .......... .......... .......... ..........
501       ......
//
ID   Yw2oyyyy   ami; 116 BP.
XX
AC   ARB_EA6926A3;
OS
YP_006762911_1_723_838_two_component_system_sensor_histidine_kinase_hy
brid_related_to_ArcB_Desulfobacula_toluolica_Tol2_
XX
XX
XX
XX
XX
XX
FT   source          1..116
XX
SQ   Sequence 116 BP;
1         .......... .......... .......... .......... ..........
51        .......... ..MVVDDD-P VNLQVLINH- -LE------- S----GH---
101       --FK------ --AQPFA--G GCQALDC--- ---------- ----------
151       -------L-- ---------- E----KGP-- L--------- ------P---
201       -----D---- --L-ILLDIM M-P--E-I-- ---SG--YDV CR---K--I-
251       --R----E-- ---------- ---------- ---------- ----------
301       ---------- ---------- ------K--- ---Y-----S ------AS--
351       E--------- ---L--P--- ----V--ILL T---AKN--- ----RLQDLV
```

```
401       EGFTVGS--- ---NDYL-TK PFFKDEL--- ---------- MARIKTQL..
451       .......... .......... .......... .......... ..........
501       ......
//
ID   Yd0eyyyy   ami; 116 BP.
XX
AC   ARB_E71D5D85;
OS
YP_007070807_1_734_849_adenylate_guanylate_cyclase_with_integral_membr
ane_sensor_Leptolyngbya_sp_PCC_7376_
XX
XX
XX
XX
XX
XX
FT   source          1..116
XX
SQ   Sequence 116 BP;
1         .......... .......... .......... .......... ..........
51        .......... ...LIVDDEP VNVQVLVNH- -LS------- L----QD---
101       --YA------ --IAQAS--N GIEALQL--- ---------- ----------
151       -------I-- ---------- E----SGI-- K--------- ------P---
201       -----D---- --I-VLLDIM M-P--R-M-- ---TG--YEV CK---K--L-
251       --R----E-- ---------- ---------- ---------- ----------
301       ---------- ---------- ------K--- ---Y-----S ------LD--
351       E--------- ---L--P--- ----V--VML T---AKN--- ----QVNDLV
401       AGFNCGA--- ---NDYL-TK PISKTEL--- ---------- LARIKTHL..
451       .......... .......... .......... .......... ..........
501       ......
//
ID   Yd0enyyy   ami; 116 BP.
XX
AC   ARB_C5D147F3;
OS
YP_007072482_1_719_834_adenylate_guanylate_cyclase_with_integral_membr
ane_sensor_Leptolyngbya_sp_PCC_7376_
XX
XX
XX
XX
XX
XX
FT   source          1..116
XX
SQ   Sequence 116 BP;
1         .......... .......... .......... .......... ..........
51        .......... ...LIVDDEP INRQVLINH- -LS------- P----YD---
101       --YA------ --VTEAS--N GQEALDI--- ---------- ----------
151       -------I-- ---------- N----DGL-- L--------- ------P---
```

```
201       -----D---- --L-ILLDIM M-P--N-M-- ---TG--YEV CH---I--L-
251       --R----Q-- ---------- ---------- ---------- ----------
301       ---------- ---------- ------K--- ---F-----L ------AH--
351       E--------- ---L--P--- ----I--VML T---AKN--- ----QVESIV
401       EGFTAGA--- ---NDYL-TK PIQKQEL--- ---------- LARMKTHL..
451       .......... .......... .......... .......... ..........
501       ......
//
ID   Yi6gyyyy   ami; 116 BP.
XX
AC   ARB_ACFCC148;
OS
YP_007058396_1_737_852_signal_transduction_histidine_kinase_Rivularia_
sp_PCC_7116_
XX
XX
XX
XX
XX
XX
FT   source          1..116
XX
SQ   Sequence 116 BP;
1         .......... .......... .......... .......... ..........
51        .......... ...LIVDDEP VNLQVLNNH- -LS------- L----QK---
101       --YR------ --VTQAS--S GAEALEI--- ---------- ----------
151       -------I-- ---------- E----NGD-- K--------- ------V---
201       -----D---- --L-MLLDIM M-P--K-M-- ---SG--YKV CA---K--L-
251       --R----E-- ---------- ---------- ---------- ----------
301       ---------- ---------- ------K--- ---Y-----A ------PH--
351       Q--------- ---L--P--- ----V--VML T---AKN--- ----QVTNIV
401       TGFKFGA--- ---NDYL-TK PFHKDEL--- ---------- LTRIKSHL..
451       .......... .......... .......... .......... ..........
501       ......
//
ID   Yi6gnyyy   ami; 116 BP.
XX
AC   ARB_CAE2244E;
OS
YP_007058398_1_738_853_signal_transduction_histidine_kinase_Rivularia_
sp_PCC_7116_
XX
XX
XX
XX
XX
XX
FT   source          1..116
XX
SQ   Sequence 116 BP;
```

```
1         .......... .......... .......... .......... ..........
51        .......... ...LIVDDEP VNLQVLNNH- -LS------- L----HK---
101       --YR------ --VTQAL--N GREALNI--- ---------- ----------
151       -------V-- ---------- D----KEE-- K--------- ------I---
201       -----D---- --L-ILLDIM M-P--K-M-- ---SG--YEV CA---A--L-
251       --R----E-- ---------- ---------- ---------- ----------
301       ---------- ---------- ------K--- ---Y-----P ------AH--
351       Q--------- ---L--P--- ----V--IML T---AKN--- ----QVTDIV
401       TGFKFGA--- ---NDYL-TK PFHKDEL--- ---------- LTRIKSHL..
451       .......... .......... .......... .......... ..........
501       ......
//
ID   Yi8nyyyy   ami; 116 BP.
XX
AC   ARB_706CDA8F;
OS
YP_007083863_1_598_713_serine_phosphatase_RsbU_regulator_of_sigma_subu
nit_Oscillatoria_acuminata_PCC_6304_
XX
XX
XX
XX
XX
XX
FT   source          1..116
XX
SQ   Sequence 116 BP;
1         .......... .......... .......... .......... ..........
51        .......... ...LIVDDEP INLQVLVNM- -LS------- L----QN---
101       --YT------ --VIQAK--N GLDALEL--- ---------- ----------
151       -------L-- ---------- H----KGF-- K--------- ------P---
201       -----D---- --L-VLLDVM M-P--K-M-- ---TG--YEV TQ---K--I-
251       --R----E-- ---------- ---------- ---------- ----------
301       ---------- ---------- ------T--- ---W-----Q ------AS--
351       E--------- ---M--P--- ----V--LLL T---AKN--- ----QLSDLV
401       AGFESGA--- ---NDYL-TK PVEKDEL--- ---------- LARIKTHL..
451       .......... .......... .......... .......... ..........
501       ......
//
ID   Yi9pyyyy   ami; 116 BP.
XX
AC   ARB_AE0FF42D;
OS
YP_007084184_1_636_751_periplasmic_ligand_binding_sensor_domain_contai
ning_protein_Oscillatoria_acuminata_PCC_6304_
XX
XX
XX
XX
XX
```

```
XX
FT   source          1..116
XX
SQ   Sequence 116 BP;
1          .......... .......... .......... .......... ..........
51         .......... ...LIVDDEP VNLQVLVNH- -LS------- L----QN---
101        --YA------ --ITQAS--N GMEALDV--- ---------- ----------
151        -------I-- ---------- E----RGF-- K--------- ------P---
201        -----D---- --L-ILLDVM M-P--K-M-- ---TG--LEV CQ---K--L-
251        --R----D-- ---------- ---------- ---------- ----------
301        ---------- ---------- ------V--- ---F-----P ------AT--
351        E--------- ---V--P--- ----I--LML T---AKT--- ----QIDVLV
401        EGFGSGA--- ---NDYL-TK PISKNEL--- ---------- LARIKTHL..
451        .......... .......... .......... .......... ..........
501        ......
//
ID   YO3scyy2   ami; 116 BP.
XX
AC   ARB_BD77F0C2;
OS   YP_007088849_1_1852_1967_ATPase_Oscillatoria_acuminata_PCC_6304_
XX
XX
XX
XX
XX
XX
FT   source          1..116
XX
SQ   Sequence 116 BP;
1          .......... .......... .......... .......... ..........
51         .......... ...LIVDDEP INLQVLVNH- -LS------- L----EN---
101        --YA------ --IATAS--N GPEALAE--- ---------- ----------
151        -------M-- ---------- E----NGL-- R--------- ------P---
201        -----D---- --L-ILLDVM M-P--K-M-- ---TG--YEV CQ---R--I-
251        --R----E-- ---------- ---------- ---------- ----------
301        ---------- ---------- ------K--- ---F-----P ------AN--
351        E--------- ---L--P--- ----V--VLL T---ARN--- ----QASDMM
401        EAFGSGA--- ---NDYL-TK PISKNEL--- ---------- LARIKMHI..
451        .......... .......... .......... .......... ..........
501        ......
//
ID   Yn2eyyyy   ami; 116 BP.
XX
AC   ARB_20FAE976;
OS
YP_007120051_1_602_717_serine_phosphatase_RsbU_regulator_of_sigma_subu
nit_Microcoleus_sp_PCC_7113_
XX
XX
XX
```

```
XX
XX
XX
FT   source          1..116
XX
SQ   Sequence 116 BP;
1          .......... .......... .......... .......... ..........
51         .......... ...LIVDDEP VNRQVFANY- -LA------- L----HN---
101        --YG------ --INQAS--N GLEALAL--- ---------- ----------
151        -------L-- ---------- E----RGL-- K--------- ------P---
201        -----D---- --L-VLLDVM M-P--R-L-- ---TG--YEV TR---K--I-
251        --R----E-- ---------- ---------- ---------- ----------
301        ---------- ---------- ------T--- ---W-----Q ------AN--
351        E--------- ---L--P--- ----V--LLL S---AKN--- ----QVSDLV
401        VGLEVGA--- ---NDYL-TK PISKDEL--- ---------- LARIKTHL..
451        .......... .......... .......... .......... ..........
501        ......
//
ID   YPdmnYy2   ami; 116 BP.
XX
AC   ARB_F21BA7C8;
OS
YP_007120333_1_581_696_PAS_domain_containing_protein_Microcoleus_sp_PC
C_7113_
XX
XX
XX
XX
XX
XX
FT   source          1..116
XX
SQ   Sequence 116 BP;
1          .......... .......... .......... .......... ..........
51         .......... ...LVVDDEA INLQVVANH- -LS------- L----QN---
101        --YG------ --IVQAS--S GIEALEK--- ---------- ----------
151        -------I-- ---------- H----NGL-- R--------- ------P---
201        -----D---- --L-ILLDIM M-P--K-M-- ---SG--YQV CQ---K--I-
251        --R----E-- ---------- ---------- ---------- ----------
301        ---------- ---------- ------Q--- ---F-----P ------AS--
351        E--------- ---M--P--- ----V--VML T---AKN--- ----QVSNLV
401        AGLDAGA--- ---NDYL-TK PILKNEL--- ---------- LARIKTHI..
451        .......... .......... .......... .......... ..........
501        ......
//
ID   YlhstYyy   ami; 116 BP.
XX
AC   ARB_46AF6EFD;
OS
YP_007122515_1_696_811_signal_transduction_histidine_kinase_Microcoleu
```

```
s_sp_PCC_7113_
XX
XX
XX
XX
XX
XX
FT   source          1..116
XX
SQ   Sequence 116 BP;
1          .......... .......... .......... .......... ..........
51         .......... ...LIVDDEP VNLQVLVNH- -LS------- L----QN---
101        --YA------ --ITQAA--N GMEALEL--- ---------- ----------
151        -------I-- ---------- D----RGF-- K--------- ------P---
201        -----D---- --L-ILLDIM M-P--K-M-- ---TG--YEL CQ---K--I-
251        --R----E-- ---------- ---------- ---------- ----------
301        ---------- ---------- ------R--- ---F-----P ------AN--
351        E--------- ---L--P--- ----V--VLL T---AKN--- ----QVSDLV
401        EGFSAGA--- ---NDYL-TK PVLKNEL--- ---------- LARIKTHI..
451        .......... .......... .......... .......... ..........
501        ......
//
ID   Yd7eyyyy   ami; 116 BP.
XX
AC   ARB_19D3FBF5;
OS
YP_007114488_1_723_838_adenylate_guanylate_cyclase_with_integral_membr
ane_sensor_Oscillatoria_nigro_viridis_PCC_7112_
XX
XX
XX
XX
XX
XX
FT   source          1..116
XX
SQ   Sequence 116 BP;
1          .......... .......... .......... .......... ..........
51         .......... ...LVVDDEP INIQVIINS- -LS------- I----EN---
101        --YE------ --ITQAS--N GLEAVNL--- ---------- ----------
151        -------I-- ---------- G----SGF-- K--------- ------P---
201        -----D---- --L-ILLDVM M-P--Q-M-- ---TG--YEV CR---E--V-
251        --R----K-- ---------- ---------- ---------- ----------
301        ---------- ---------- ------K--- ---Y-----S ------PL--
351        E--------- ---M--P--- ----I--LML T---AKN--- ----QTTDLV
401        EAFNLEA--- ---NDYV-TK PFIKKEL--- ---------- LARINTQI..
451        .......... .......... .......... .......... ..........
501        ......
//
ID   Yd7enyyy   ami; 116 BP.
```

```
XX
AC   ARB_8C095A21;
OS
YP_007114489_1_683_798_adenylate_guanylate_cyclase_with_integral_membr
ane_sensor_Oscillatoria_nigro_viridis_PCC_7112_
XX
XX
XX
XX
XX
XX
FT   source          1..116
XX
SQ   Sequence 116 BP;
1         .......... .......... .......... .......... ..........
51        .......... ...LIVDDEA VNIQVLANN- -LL------- L----EK---
101       --YA------ --LAKAS--S GREALAL--- ---------- ----------
151       -------I-- ---------- D----RGY-- K--------- ------P---
201       -----D---- --L-ILLDLM M-P--R-M-- ---TG--YEV CE---K--I-
251       --R----E-- ---------- ---------- ---------- ----------
301       ---------- ---------- ------K--- ---F-----T ------AI--
351       E--------- ---V--P--- ----I--VML T---AKN--- ----QVSDLV
401       QGFNAGA--- ---NDFL-TK PFVKNEL--- ---------- LARIKTHI..
451       .......... .......... .......... .......... ..........
501       ......
//
ID   Yg2naYy2   ami; 116 BP.
XX
AC   ARB_D7AED625;
OS
YP_007149058_1_734_849_signal_transduction_histidine_kinase_Cylindrosp
ermum_stagnale_PCC_7417_
XX
XX
XX
XX
XX
XX
FT   source          1..116
XX
SQ   Sequence 116 BP;
1         .......... .......... .......... .......... ..........
51        .......... ...LIVDDEP VNRQVLVNH- -LS------- L----YN---
101       --YE------ --ITEAA--S GIEALAI--- ---------- ----------
151       -------L-- ---------- E----GGL-- K--------- ------P---
201       -----D---- --L-ILLDVM M-P--R-M-- ---TG--YDV TR---Q--I-
251       --R----Q-- ---------- ---------- ---------- ----------
301       ---------- ---------- ------T--- ---W-----Q ------AS--
351       E--------- ---L--P--- ----I--VLL T---ARN--- ----QVSDLV
401       VGFEAGA--- ---NDYL-TK PISKDEL--- ---------- LARIKVHC..
```

```
451       .......... .......... .......... .......... ..........
501       ......
//
ID   Yw5oyyyy   ami; 116 BP.
XX
AC   ARB_30D8231E;
OS
YP_005069038_1_874_989_two_component_hybrid_sensor_and_regulator_Arthr
ospira_platensis_NIES_39_
XX
XX
XX
XX
XX
XX
FT   source          1..116
XX
SQ   Sequence 116 BP;
1         .......... .......... .......... .......... ..........
51        .......... ...LIVDDDP INLQVLANY- -LY------- L----EN---
101       --YA------ --VTQAT--N GMEALEI--- ---------- ----------
151       -------L-- ---------- E----SGF-- I--------- ------P---
201       -----D---- --L-ILLDVM M-P--R-M-- ---TG--YEV TE---K--I-
251       --R----E-- ---------- ---------- ---------- ----------
301       ---------- ---------- ------D--- ---W-----P ------PH--
351       Q--------- ---L--P--- ----I--MML T---AKN--- ----RISDLV
401       VGLELGA--- ---NDYL-SK PLNKEEL--- ---------- LARIKTHI..
451       .......... .......... .......... .......... ..........
501       ......
//
ID   W11Ptyyy   ami; 117 BP.
XX
AC   ARB_52C02E7A;
OS
WP_002694524_1_888_1004_two_component_hybrid_sensor_and_regulator_Micr
oscilla_marina_
XX
XX
XX
XX
XX
XX
FT   source          1..117
XX
SQ   Sequence 117 BP;
1         .......... .......... .......... .......... ..........
51        .......... ...LVVDDEP VNLQVLENH- -LS------- I----QN---
101       --YN------ --ITQAS--D GLKALKI--- ---------- ----------
151       -------I-- ---------- K----ESKE- P--------- ------F---
201       -----D---- --I-ILLDVM M-P--K-M-- ---SG--YEV CR---T--I-
```

```
251       --R----E-- ---------- ---------- ---------- ----------
301       ---------- ---------- ------R--- ---F-----P ------LV--
351       E--------- ---L--P--- ----V--LML T---AKN--- ----QPKDIV
401       EGFDAGA--- ---NDYL-TK PFSKVEL--- ---------- LSRIKTHV..
451       .......... .......... .......... .......... ..........
501       ......
//
ID   W42PYyy2   ami; 116 BP.
XX
AC   ARB_F1759CFB;
OS
WP_006101034_1_713_828_histidine_kinase_Coleofasciculus_chthonoplastes
_
XX
XX
XX
XX
XX
XX
FT   source          1..116
XX
SQ   Sequence 116 BP;
1         .......... .......... .......... .......... ..........
51        .......... ...LIVDDEP TNLQVIVNH- -LS------- L----QN---
101       --YA------ --ITQAS--S ALEALEL--- ---------- ----------
151       -------I-- ---------- R----LGF-- K--------- ------P---
201       -----D---- --L-ILLDIM M-P--Q-M-- ---TG--YEL CK---K--L-
251       --R----E-- ---------- ---------- ---------- ----------
301       ---------- ---------- ------Q--- ---F-----L ------PN--
351       E--------- ---L--P--- ----I--VLL T---AKN--- ----QISDLV
401       EGFAVGA--- ---NDYL-TK PVLKNEL--- ---------- LARIKTHL..
451       .......... .......... .......... .......... ..........
501       ......
//
ID   W45PYyyy   ami; 116 BP.
XX
AC   ARB_940736E;
OS
WP_006104393_1_581_696_PAS_fold_family_Coleofasciculus_chthonoplastes_
XX
XX
XX
XX
XX
XX
FT   source          1..116
XX
SQ   Sequence 116 BP;
1         .......... .......... .......... .......... ..........
51        .......... ...LVVDDEP INLQVVTNH- -LS------- V----HN---
```

```
101       --YA------ --IVQAT--S GIEALAK--- ---------- ----------
151       -------I-- ---------- E----QGF-- K--------- ------P---
201       -----D---- --L-VILDIM M-P--K-M-- ---SG--YEV CQ---K--I-
251       --R----Q-- ---------- ---------- ---------- ----------
301       ---------- ---------- ------C--- ---F-----P ------AT--
351       E--------- ---M--P--- ----I--ILL T---AKE--- ----QVSDLV
401       EGLSLGA--- ---NDYL-TK PISKHEL--- ---------- IARIKTHL..
451       .......... .......... .......... .......... ..........
501       ......
//
ID   W62Payyy   ami; 116 BP.
XX
AC   ARB_5EADF5E1;
OS
WP_006619087_1_722_837_adenylate_guanylate_cyclase_Arthrospira_platens
is_
XX
XX
XX
XX
XX
XX
FT   source          1..116
XX
SQ   Sequence 116 BP;
1         .......... .......... .......... .......... ..........
51        .......... ...LIADDEP VNLQVISNY- -LS------- L----EN---
101       --YT------ --IVSAL--N GIEALAL--- ---------- ----------
151       -------I-- ---------- E----EGF-- K--------- ------P---
201       -----D---- --L-VLLDLM M-P--Q-M-- ---TG--YEV CE---K--L-
251       --R----E-- ---------- ---------- ---------- ----------
301       ---------- ---------- ------H--- ---F-----E ------AN--
351       Q--------- ---L--P--- ----I--LML T---AKN--- ----QVSDLV
401       HGLGVGA--- ---NDYL-TK PISKHEL--- ---------- LARIKTHL..
451       .......... .......... .......... .......... ..........
501       ......
//
ID   W88Pgyyy   ami; 116 BP.
XX
AC   ARB_DD6AF1C5;
OS   WP_007353506_1_732_847_guanylate_cyclase_Oscillatoria_
XX
XX
XX
XX
XX
XX
FT   source          1..116
XX
SQ   Sequence 116 BP;
```

```
1         .......... .......... .......... .......... ..........
51        .......... ...LIVDDEP VNIQVLINN- -LS------- V----LK---
101       --YE------ --VTQAT--N GFEALEI--- ---------- ----------
151       -------I-- ---------- E----NGF-- R--------- ------P---
201       -----D---- --L-ILLDVM M-P--R-M-- ---SG--YEV CQ---I--L-
251       --R----E-- ---------- ---------- ---------- ----------
301       ---------- ---------- ------K--- ---F-----P ------AI--
351       E--------- ---L--P--- ----I--VML T---AKN--- ----QVTDLV
401       EGFNSGA--- ---NDYL-SK PFSKNEL--- ---------- LARIKTHI..
451       .......... .......... .......... .......... ..........
501       ......
//
ID   W88Pgyy0   ami; 116 BP.
XX
AC   ARB_BF701369;
OS   WP_007356084_1_714_829_guanylate_cyclase_Oscillatoria_
XX
XX
XX
XX
XX
XX
FT   source          1..116
XX
SQ   Sequence 116 BP;
1         .......... .......... .......... .......... ..........
51        .......... ...LIVDDEP INRQVLINN- -LS------- L----YN---
101       --YA------ --ITEAS--N GQEALTA--- ---------- ----------
151       -------M-- ---------- E----NGF-- I--------- ------P---
201       -----D---- --L-ILLDLM M-P--Q-M-- ---TG--YEV CQ---K--I-
251       --R----A-- ---------- ---------- ---------- ----------
301       ---------- ---------- ------R--- ---F-----P ------TY--
351       E--------- ---L--P--- ----I--VML T---AKN--- ----QVADIV
401       EGFESGA--- ---NDYL-SK PIQKQEM--- ---------- LARIKTHI..
451       .......... .......... .......... .......... ..........
501       ......
//
ID   A08WPyyy   ami; 116 BP.
XX
AC   ARB_75ABD328;
OS   WP_008187689_1_732_847_histidine_kinase_Moorea_producens_
XX
XX
XX
XX
XX
XX
FT   source          1..116
XX
SQ   Sequence 116 BP;
```

```
1          .......... .......... .......... .......... ..........
51         .......... ...LIVDDEP VNLQVLVNH- -LS------- L----HN---
101        --YK------ --ITQAV--N GLDAFTK--- ---------- ----------
151        -------I-- ---------- R----QGY-- R--------- ------P---
201        -----D---- --L-ILLDIM M-P--K-M-- ---TG--YEF CK---K--I-
251        --R----N-- ---------- ---------- ---------- ----------
301        ---------- ---------- ------K--- ---Y-----L ------PN--
351        E--------- ---L--P--- ----V--VLL T---AKN--- ----QISDLV
401        AGFAAGA--- ---NDYL-TK PISKNEL--- ---------- LARIKTHL..
451        .......... .......... .......... .......... ..........
501        ......
//
ID   A0HWPyyy   ami; 116 BP.
XX
AC   ARB_78914018;
OS
WP_008310939_1_713_828_signal_transduction_histidine_kinase_Leptolyngb
ya_sp_PCC_6406_
XX
XX
XX
XX
XX
XX
FT   source          1..116
XX
SQ   Sequence 116 BP;
1          .......... .......... .......... .......... ..........
51         .......... ...LIVDDEP INRQVLINN- -LS------- L----YN---
101        --YA------ --ITEAS--N GQEALKI--- ---------- ----------
151        -------I-- ---------- D----EGF-- L--------- ------P---
201        -----D---- --L-ILLDVM M-P--R-M-- ---TG--YEV CK---K--I-
251        --R----D-- ---------- ---------- ---------- ----------
301        ---------- ---------- ------R--- ---F-----P ------SY--
351        E--------- ---L--P--- ----I--VML T---AKN--- ----QVADIV
401        EGFESGA--- ---NDYL-CK PIQKQEM--- ---------- LARIKTHL..
451        .......... .......... .......... .......... ..........
501        ......
//
ID   W54Pryy3   ami; 116 BP.
XX
AC   ARB_6D480684;
OS
WP_008317381_1_788_903_response_regulator_with_CheY_like_receiver_doma
in_and_winged_helix_DNA_binding_domain_Leptolyngbya_sp_PCC_6406_
XX
XX
XX
XX
XX
```

```
XX
FT   source          1..116
XX
SQ   Sequence 116 BP;
1          .......... .......... .......... .......... ..........
51         .......... ...LVVDDEP VNRQVIVNH- -LS------- V----HS---
101        --YR------ --VTQAS--N GPEALAM--- ---------- ----------
151        -------L-- ---------- S----NGL-- L--------- ------P---
201        -----D---- --L-ILLDVM M-P--R-M-- ---TG--YEV CR---Q--V-
251        --R----E-- ---------- ---------- ---------- ----------
301        ---------- ---------- ------Q--- ---H-----P ------AY--
351        A--------- ---L--P--- ----I--VML T---AKN--- ----QVADLV
401        EGLSAGA--- ---NDYL-TK PVSKGEL--- ---------- LARLNTHL..
451        .......... .......... .......... .......... ..........
501        ......
//
ID   A1DWPyyy   ami; 116 BP.
XX
AC   ARB_291BCA24;
OS   WP_009782294_1_893_1008_adenylate_cyclase_Lyngbya_sp_PCC_8106_
XX
XX
XX
XX
XX
XX
FT   source          1..116
XX
SQ   Sequence 116 BP;
1          .......... .......... .......... .......... ..........
51         .......... ...LIVDDDP INLQVLVNY- -LC------- L----QN---
101        --YA------ --VTQAS--S GIEAMEI--- ---------- ----------
151        -------L-- ---------- E----AGY-- I--------- ------P---
201        -----D---- --L-ILLDVM M-P--R-M-- ---TG--YEV TR---R--I-
251        --R----E-- ---------- ---------- ---------- ----------
301        ---------- ---------- ------T--- ---W-----P ------PH--
351        Q--------- ---L--P--- ----I--MML T---AKN--- ----QVSDLV
401        AGLEVGA--- ---NDYL-SK PLNKDEL--- ---------- LARIKTHI..
451        .......... .......... .......... .......... ..........
501        ......
//
ID   A1IWPyyy   ami; 116 BP.
XX
AC   ARB_E57DF868;
OS
WP_009785933_1_640_755_two_component_sensor_histidine_kinase_Lyngbya_s
p_PCC_8106_
XX
XX
XX
```

```
XX
XX
XX
FT   source          1..116
XX
SQ   Sequence 116 BP;
1         .......... .......... .......... .......... ..........
51        .......... ...FIVDDEP INLQVLMNM- -LS------- L----HD---
101       --YT------ --ITQAS--D GEEALAI--- ---------- ----------
151       -------L-- ---------- E----TGY-- K--------- ------P---
201       -----D---- --I-ILLDVM M-P--K-M-- ---TG--YEV TQ---K--I-
251       --R----E-- ---------- ---------- ---------- ----------
301       ---------- ---------- ------R--- ---F-----T ------TT--
351       E--------- ---L--P--- ----I--LLL T---AKT--- ----QVQDIV
401       TGLGFGA--- ---NDYL-NK PIAKDEL--- ---------- LARMKTQI..
451       .......... .......... .......... .......... ..........
501       ......
//
ID   A1FWPyy0   ami; 116 BP.
XX
AC   ARB_51FB7714;
OS   WP_009786545_1_719_834_guanylate_cyclase_Lyngbya_sp_PCC_8106_
XX
XX
XX
XX
XX
XX
FT   source          1..116
XX
SQ   Sequence 116 BP;
1         .......... .......... .......... .......... ..........
51        .......... ...LVVDDEP VNIQVIANN- -LS------- L----QN---
101       --YA------ --ITQAT--N GLEALTL--- ---------- ----------
151       -------I-- ---------- E----KGF-- K--------- ------P---
201       -----D---- --L-ILLDVM M-P--R-M-- ---TG--YEV CQ---K--L-
251       --R----E-- ---------- ---------- ---------- ----------
301       ---------- ---------- ------K--- ---Y-----L ------HS--
351       E--------- ---L--P--- ----I--LML T---AKN--- ----QVTDLV
401       DGLSSGA--- ---NDYL-SK PFSKQEL--- ---------- LARIKTHL..
451       .......... .......... .......... .......... ..........
501       ......
//
ID   WhhtcYy5   ami; 116 BP.
XX
AC   ARB_AB48E59A;
OS   WP_017304826_1_746_861_hypothetical_protein_Spirulina_subsalsa_
XX
XX
XX
```

```
XX
XX
XX
FT   source          1..116
XX
SQ   Sequence 116 BP;
1          .......... .......... .......... .......... ..........
51         .......... ...LIVDDEP VNLQVLKNN- -LS------- L----QS---
101        --YS------ --ITQAT--D GLEALRI--- ---------- ----------
151        -------I-- ---------- E----NGF-- V--------- ------P---
201        -----D---- --L-MLLDVM M-P--R-M-- ---TG--YEV SQ---K--I-
251        --R----E-- ---------- ---------- ---------- ----------
301        ---------- ---------- ------K--- ---F-----S ------AS--
351        E--------- ---L--P--- ----I--VML T---AKN--- ----QVTDLV
401        EGFGAGA--- ---NDYL-TK PFSKNEL--- ---------- LARIKTHI..
451        .......... .......... .......... .......... ..........
501        ......
//
ID   WhhtcYy6   ami; 116 BP.
XX
AC   ARB_73E3BED7;
OS   WP_017305029_1_560_675_hypothetical_protein_Spirulina_subsalsa_
XX
XX
XX
XX
XX
XX
FT   source          1..116
XX
SQ   Sequence 116 BP;
1          .......... .......... .......... .......... ..........
51         .......... ...LVVDDDP INLQVIINH- -LS------- L----ES---
101        --YQ------ --IIPVR--S GKEALTL--- ---------- ----------
151        -------L-- ---------- A----EGL-- Q--------- ------P---
201        -----D---- --L-MLLDVM M-P--K-M-- ---SG--YEV CR---R--I-
251        --R----E-- ---------- ---------- ---------- ----------
301        ---------- ---------- ------T--- ---I-----P ------MN--
351        E--------- ---L--P--- ----I--VML T---ANT--- ----QVSELV
401        ACLNVGA--- ---NDYL-TK PLSKNEL--- ---------- LARLKTHL..
451        .......... .......... .......... .......... ..........
501        ......
//
ID   WhrtnYy6   ami; 116 BP.
XX
AC   ARB_24EF9D64;
OS
WP_017660438_1_740_855_hypothetical_protein_Geitlerinema_sp_PCC_7105_
XX
XX
```

```
XX
XX
XX
XX
FT   source          1..116
XX
SQ   Sequence 116 BP;
1          .......... .......... .......... .......... ..........
51         .......... ...LIVDDEP INLQVLINH- -LS------- P----EH---
101        --YQ------ --ITQAS--N GLEALKS--- ---------- ----------
151        -------I-- ---------- E----NGL-- K--------- ------P---
201        -----D---- --L-ILLDVM M-P--K-M-- ---TG--YEV TA---K--L-
251        --R----E-- ---------- ---------- ---------- ----------
301        ---------- ---------- ------K--- ---Y-----S ------AH--
351        E--------- ---L--P--- ----I--LML T---AKT--- ----QAEDIV
401        QGLNLGA--- ---NDYL-TK PINKKEL--- ---------- LARLQTHL..
451        .......... .......... .......... .......... ..........
501        ......
//
ID   WhOscYy8   ami; 116 BP.
XX
AC   ARB_74E537A3;
OS
WP_017718872_1_505_620_hypothetical_protein_Oscillatoria_sp_PCC_10802_
XX
XX
XX
XX
XX
XX
FT   source          1..116
XX
SQ   Sequence 116 BP;
1          .......... .......... .......... .......... ..........
51         .......... ...LIVDDEP VNLQVLVNY- -LS------- L----HK---
101        --YT------ --TARAS--S GREALKI--- ---------- ----------
151        -------I-- ---------- Q----QGF-- Q--------- ------P---
201        -----D---- --L-VLLDVM M-P--R-M-- ---TG--YEA CR---K--I-
251        --R----Q-- ---------- ---------- ---------- ----------
301        ---------- ---------- ------M--- ---F-----S ------AS--
351        E--------- ---L--P--- ----V--VLL T---AKN--- ----QVSDLV
401        EGFRAGA--- ---NDYL-TK PISKDEL--- ---------- LARIKTHI..
451        .......... .......... .......... .......... ..........
501        ......
//
ID   WfESFYy2   ami; 116 BP.
XX
AC   ARB_B9C025BD;
OS
WP_018397756_1_654_769_hypothetical_protein_filamentous_cyanobacterium
```

```
_ESFC_1_
XX
XX
XX
XX
XX
XX
FT   source          1..116
XX
SQ   Sequence 116 BP;
1          .......... .......... .......... .......... ..........
51         .......... ...LIVDDEP INLQILLNN- -LS------- L----EN---
101        --YC------ --VIQAQ--N GLEALRL--- ---------- ----------
151        -------L-- ---------- D----QGL-- Q--------- ------P---
201        -----D---- --L-ILLDVM M-P--H-L-- ---TG--YEV AQ---K--I-
251        --R----E-- ---------- ---------- ---------- ----------
301        ---------- ---------- ------L--- ---F-----L ------PS--
351        Q--------- ---L--P--- ----I--VML T---AKN--- ----RDTDVL
401        EGFACGA--- ---NDYL-LK PFSKKEL--- ---------- LARIKTHL..
451        .......... .......... .......... .......... ..........
501        ......
//
ID   Wh7ypyy2   ami; 116 BP.
XX
AC   ARB_38045A1F;
OS   WP_020286031_1_710_825_hypothetical_protein_Osedax_symbiont_Rs2_
XX
XX
XX
XX
XX
XX
FT   source          1..116
XX
SQ   Sequence 116 BP;
1          .......... .......... .......... .......... ..........
51         .......... ...LVVDDEP INLRVIGNH- -LS------- L----YR---
101        --YR------ --IKEAT--S GAQALEL--- ---------- ----------
151        -------I-- ---------- E----DGL-- K--------- ------A---
201        -----D---- --L-ILLDVM M-P--Q-M-- ---NG--FKV CQ---I--L-
251        --R----Q-- ---------- ---------- ---------- ----------
301        ---------- ---------- ------T--- ---Y-----S ------AN--
351        Q--------- ---L--P--- ----I--ILV T---AKN--- ----QATDLI
401        AGFASGA--- ---NDYL-TK PYSKHEL--- ---------- LMRINLHI..
451        .......... .......... .......... .......... ..........
501        ......
//
ID   Yu9lyyyy   ami; 116 BP.
XX
AC   ARB_C3022E76;
```

```
OS
YP_526141_1_1147_1262_response_regulator_receiver_domain_containing_pr
otein_Saccharophagus_degradans_2_40_
XX
XX
XX
XX
XX
XX
FT   source          1..116
XX
SQ   Sequence 116 BP;
1         .......... .......... .......... .......... ..........
51        .......... ...LIVDDEP VNRKVLANY- -LS------- L----KY---
101       --YT------ --VHEVR--S GMEAITY--- ---------- ----------
151       -------V-- ---------- R----NGP-- K--------- ------V---
201       -----D---- --L-ILLDIM M-P--K-M-- ---SG--YEA CK---Q--L-
251       --R----Q-- ---------- ---------- ---------- ----------
301       ---------- ---------- ------Q--- ---Y-----S ------AN--
351       E--------- ---L--P--- ----I--ILL T---ARS--- ----QVSDLV
401       TGFNSGA--- ---NDFI-TK PIAKEEL--- ---------- LARVHTHL..
451       .......... .......... .......... .......... ..........
501       ......
//
ID   Yy0pyyyy   ami; 115 BP.
XX
AC   ARB_468C0225;
OS
156975687_YP_001446594_1_725_839_hypothetical_protein_VIBHAR_03422_Vib
rio_campbellii_ATCC_BAA_1116_
XX
XX
XX
XX
XX
XX
FT   source          1..115
XX
SQ   Sequence 115 BP;
1         .......... .......... .......... .......... ..........
51        .......... ...YVADDEP VNLRVLESF- -LR------- L----EG---
101       --YR------ --VRTIS--D GPETLAL--- ---------- ----------
151       -------I-- ---------- E----QE--- K--------- ------P---
201       -----E---- --L-LLLDIM M-P--G-M-- ---SG--YQV CS---E--L-
251       --R----E-- ---------- ---------- ---------- ----------
301       ---------- ---------- ------T--- ---Y-----D ------HA--
351       E--------- ---L--P--- ----I--IML T---ALS--- ----QTEDRV
401       RGFEAGA--- ---NDYL-TK PFNKQEL--- ---------- AARIQAHL..
451       .......... .......... .......... .......... ..........
501       ......
```

```
//
ID   Yv9iyyyy   ami; 115 BP.
XX
AC   ARB_FF985C66;
OS
209695992_YP_002263922_1_723_837_chitin_degradation_sensor_protein_Ali
ivibrio_salmonicida_LFI1238_
XX
XX
XX
XX
XX
XX
FT   source          1..115
XX
SQ   Sequence 115 BP;
1         .......... .......... .......... .......... ..........
51        .......... ...LVADDEP VNLQVLSSF- -LK------- L----EG---
101       --YR------ --VKTAN--D GPETLKQ--- ---------- ----------
151       -------L-- ---------- E----EE--- T--------- ------P---
201       -----E---- --L-VLLDVM M-P--G-M-- ---SG--YEV CQ---E--V-
251       --R----K-- ---------- ---------- ---------- ----------
301       ---------- ---------- ------Q--- ---Y-----S ------HS--
351       Q--------- ---L--P--- ----I--IML T---ALN--- ----QTQDRV
401       RGFESGA--- ---NDYL-SK PFNKQEL--- ---------- AARIKAHL..
451       .......... .......... .......... .......... ..........
501       ......
//
ID   Yb8ryyyy   ami; 115 BP.
XX
AC   ARB_3F5AAD22;
OS
YP_003243024_1_666_780_integral_membrane_sensor_hybrid_histidine_kinas
e_Paenibacillus_sp_Y412MC10_
XX
XX
XX
XX
XX
XX
FT   source          1..115
XX
SQ   Sequence 115 BP;
1         .......... .......... .......... .......... ..........
51        .......... ...LIVDDDP INVQVLLQF- -LA------- -----GK---
101       --YS------ --LRSTC--S GQEALEW--- ---------- ----------
151       -------I-- ---------- Q----NGY-- K--------- ------P---
201       -----D---- --I-ALLDVM M-P--Y-V-- ---SG--LQL GE---E--I-
251       --R----R-- ---------- ---------- ---------- ----------
301       ---------- ---------- ------H--- ---Y-----N ------SG--
```

```
351       E--------- ---L--P--- ----V--IFL S---AKS--- ----QMTDLV
401       AGFEHGG--- ---NDYL-AK PVDKQEL--- ---------- LARLELHL..
451       .......... .......... .......... .......... ..........
501       ......
//
ID   Y10Pyyyy   ami; 112 BP.
XX
AC   ARB_D1DA41B3;
OS
YP_004120895_1_716_827_response_regulator_receiver_Desulfovibrio_aespo
eensis_Aspo_2_
XX
XX
XX
XX
XX
XX
FT   source          1..112
XX
SQ   Sequence 112 BP;
1         .......... .......... .......... .......... ..........
51        .......... ...LVVDDEP VNLQVVAAI- -LN------- I----EG---
101       --IS------ --FRTAA--N GAMALRM--- ---------- ----------
151       -------L-- ---------- E----DGD-- Q--------- ------P---
201       -----D---- --M-VLLDVM M-P--D-M-- ---SG--YAV CR---E--L-
251       --R----K-- ---------- ---------- ---------- ----------
301       ---------- ---------- ------T--- ---Y-----Q ------AS--
351       V--------- ---L--P--- ----I--VLL T---VKN--- ----RVEDIV
401       EGFSAGA--- ---NDYL-TK PFAREEL--- ---------- GARV......
451       .......... .......... .......... .......... ..........
501       ......
//
ID   Yk0iyyyy   ami; 112 BP.
XX
AC   ARB_3424B088;
OS
YP_004640948_1_692_803_integral_membrane_sensor_hybrid_histidine_kinas
e_Paenibacillus_mucilaginosus_KNP414_
XX
XX
XX
XX
XX
XX
FT   source          1..112
XX
SQ   Sequence 112 BP;
1         .......... .......... .......... .......... ..........
51        .......... ...LIADDEP VNLLVLQQH- -LA------- S----QP---
101       --YR------ --LVQAR--S GTEALEL--- ---------- ----------
```

```
151       -------A-- ---------- L----RS--- K--------- ------P---
201       -----D---- --L-MLLDVM M-P--G-L-- ---SG--VEV CR---Q--L-
251       --R----E-- ---------- ---------- ---------- ----------
301       ---------- ---------- ------R--- ---F-----N ------PS--
351       E--------- ---L--P--- ----V--LLL T---AKS--- ----EPRDLA
401       EGFEAGA--- ---NDYL-TK PIHRSEL--- ---------- LARVR.....
451       .......... .......... .......... .......... ..........
501       ......
//
ID   AEWYyyyy   ami; 115 BP.
XX
AC   ARB_2ED07015;
OS
363585954_AEW28962_1_724_838_sensory_box_sensor_histidine_kinase_respo
nse_regulator_Photobacterium_damselae_subsp_piscicida_
XX
XX
XX
XX
XX
XX
FT   source          1..115
XX
SQ   Sequence 115 BP;
1         .......... .......... .......... .......... ..........
51        .......... ...LVVDDEP VNLQILNNF- -LR------- L----EG---
101       --YR------ --VLTAE--S GKQALEL--- ---------- ----------
151       -------V-- ---------- Q----SH--- K--------- ------P---
201       -----A---- --L-ILLDIM M-P--E-M-- ---SG--YEV CS---Q--L-
251       --R----Q-- ---------- ---------- ---------- ----------
301       ---------- ---------- ------Q--- ---Y-----T ------PL--
351       E--------- ---L--P--- ----I--MML S---ALG--- ----QVKDRV
401       KGFECGA--- ---NDYL-TK PFNKEEL--- ---------- TARIKAHL..
451       .......... .......... .......... .......... ..........
501       ......
//
ID   YkJYyyyy   ami; 115 BP.
XX
AC   ARB_B7D8BAA;
OS
375266574_YP_005024017_1_725_839_sensory_box_sensor_histidine_kinase_r
esponse_regulator_Vibrio_sp_EJY3_
XX
XX
XX
XX
XX
XX
FT   source          1..115
XX
```

```
SQ   Sequence 115 BP;
1         .......... .......... .......... .......... ..........
51        .......... ...YVADDEP VNLRVLESF- -LR------- L----EG---
101       --YR------ --VRTVS--D GPETLAL--- ---------- ----------
151       -------V-- ---------- E----QE--- K--------- ------P---
201       -----E---- --L-LLLDIM M-P--G-M-- ---SG--YQV CN---E--L-
251       --R----Q-- ---------- ---------- ---------- ----------
301       ---------- ---------- ------T--- ---F-----D ------HA--
351       E--------- ---L--P--- ----I--IML T---ALS--- ----QTEDRV
401       RGFEAGA--- ---NDYL-SK PFNKQEL--- ---------- AARIRAHL..
451       .......... .......... .......... .......... ..........
501       ......
//
ID   Yk8iyyyy   ami; 116 BP.
XX
AC   ARB_3025293C;
OS
YP_006440036_1_542_657_integral_membrane_sensor_hybrid_histidine_kinas
e_Turneriella_parva_DSM_21527_
XX
XX
XX
XX
XX
XX
FT   source          1..116
XX
SQ   Sequence 116 BP;
1         .......... .......... .......... .......... ..........
51        .......... ...LLVDDEP VNLQVLVNQ- -LS------- L----VG---
101       --YK------ --VTVAE--S GKEAINY--- ---------- ----------
151       -------M-- ---------- E----HEP-- V--------- ------P---
201       -----D---- --A-ILLDVM M-P--G-M-- ---SG--YDV SR---I--L-
251       --R----N-- ---------- ---------- ---------- ----------
301       ---------- ---------- ------K--- ---Y-----T ------SY--
351       E--------- ---V--P--- ----I--LML T---AKN--- ----RSEDVI
401       RGFEAGA--- ---NDYI-SK PIESEVL--- ---------- LARVKTAI..
451       .......... .......... .......... .......... ..........
501       ......
//
ID   A3VWPyyy   ami; 115 BP.
XX
AC   ARB_EC020C56;
OS   446410415_WP_000488270_1_725_839_histidine_kinase_Vibrio_mimicus_
XX
XX
XX
XX
XX
XX
```

```
FT   source          1..115
XX
SQ   Sequence 115 BP;
1          .......... .......... .......... .......... ..........
51         .......... ...LVADDEP VNLRVLDSF- -LR------- L----EG---
101        --YR------ --VHTAQ--D GHEVLEA--- ---------- ----------
151        -------I-- ---------- Q----RE--- K--------- ------P---
201        -----E---- --L-LLLDIM M-P--G-M-- ---SG--YQV CE---K--L-
251        --R----Q-- ---------- ---------- ---------- ----------
301        ---------- ---------- ------T--- ---Y-----D ------HA--
351        E--------- ---L--P--- ----I--IML T---ALN--- ----QSDDRV
401        RGFEAGA--- ---NDYL-SK PFNKQEL--- ---------- AARIVAHL..
451        .......... .......... .......... .......... ..........
501        ......
//
ID   A3WWPyyy   ami; 115 BP.
XX
AC   ARB_29B4EFA3;
OS
446410426_WP_000488281_1_725_839_histidine_kinase_Vibrio_cholerae_
XX
XX
XX
XX
XX
XX
FT   source          1..115
XX
SQ   Sequence 115 BP;
1          .......... .......... .......... .......... ..........
51         .......... ...LIADDEP VNLRVLDSF- -LR------- L----EG---
101        --YR------ --VHTAQ--D GHQVLEA--- ---------- ----------
151        -------I-- ---------- K----RE--- K--------- ------P---
201        -----E---- --L-VLLDIM M-P--G-M-- ---SG--YQV CE---K--L-
251        --R----Q-- ---------- ---------- ---------- ----------
301        ---------- ---------- ------T--- ---Y-----D ------HA--
351        E--------- ---L--P--- ----I--IML T---ALN--- ----QSDDRV
401        RGFEAGA--- ---NDYL-SK PFNKQEL--- ---------- AARIVAHL..
451        .......... .......... .......... .......... ..........
501        ......
//
ID   A3YWPyyy   ami; 115 BP.
XX
AC   ARB_B7291AB9;
OS
487833820_WP_001907286_1_215_329_histidine_kinase_Vibrio_albensis_
XX
XX
XX
XX
```

```
XX
XX
FT   source          1..115
XX
SQ   Sequence 115 BP;
1          .......... .......... .......... .......... ..........
51         .......... ...LVADDEP VNLRVLDSF- -LR------- L----EG---
101        --YR------ --VHTAQ--D GHQVLEA--- ---------- ----------
151        -------M-- ---------- K----RE--- K--------- ------P---
201        -----E---- --L-VLLDIM M-P--G-M-- ---SG--YQV CE---K--L-
251        --R----Q-- ---------- ---------- ---------- ----------
301        ---------- ---------- ------T--- ---Y-----D ------HA--
351        E--------- ---L--P--- ----I--IML T---ALN--- ----QSDDRV
401        RGFEAGA--- ---NDYL-SK PFNKQEL--- ---------- AARIVAHL..
451        .......... .......... .......... .......... ..........
501        ......
//
ID   A4LWPyyy   ami; 115 BP.
XX
AC   ARB_2080F82C;
OS
490536773_WP_004401931_1_725_839_histidine_kinase_Vibrio_nigripulchrit
udo_
XX
XX
XX
XX
XX
XX
FT   source          1..115
XX
SQ   Sequence 115 BP;
1          .......... .......... .......... .......... ..........
51         .......... ...VVADDEP VNLQILDSF- -LR------- M----EG---
101        --YR------ --VKAAK--D GTETLEL--- ---------- ----------
151        -------V-- ---------- Q----QE--- K--------- ------P---
201        -----E---- --L-LLLDVM M-P--G-L-- ---SG--YQV CE---Q--V-
251        --R----L-- ---------- ---------- ---------- ----------
301        ---------- ---------- ------S--- ---Y-----D ------LS--
351        E--------- ---L--P--- ----V--IML T---ALS--- ----QTNDRI
401        KGFDSGA--- ---NDYL-TK PFNKLEL--- ---------- ASRIKAHL..
451        .......... .......... .......... .......... ..........
501        ......
//
ID   A4TWPyyy   ami; 115 BP.
XX
AC   ARB_8A39BED5;
OS
491528920_WP_005386545_1_725_839_histidine_kinase_Vibrio_alginolyticus
_
```

```
XX
XX
XX
XX
XX
XX
FT   source          1..115
XX
SQ   Sequence 115 BP;
1          .......... .......... .......... .......... ..........
51         .......... ...YVADDEP VNLRVLESF- -LR------- L----EG---
101        --YR------ --VRTVS--D GPETLAL--- ---------- ----------
151        -------V-- ---------- E----QE--- K--------- ------P---
201        -----E---- --L-LLLDIM M-P--G-M-- ---SG--YQV CS---E--L-
251        --R----E-- ---------- ---------- ---------- ----------
301        ---------- ---------- ------T--- ---H-----D ------HA--
351        E--------- ---L--P--- ----I--IML T---ALS--- ----QTEDRV
401        RGFEAGA--- ---NDYL-SK PFNKQEL--- ---------- AARIQAHL..
451        .......... .......... .......... .......... ..........
501        ......
//
ID   A4UWPyyy   ami; 115 BP.
XX
AC   ARB_2FF5B0C4;
OS   491616764_WP_005474319_1_708_822_histidine_kinase_Vibrio_sp_16_
XX
XX
XX
XX
XX
XX
FT   source          1..115
XX
SQ   Sequence 115 BP;
1          .......... .......... .......... .......... ..........
51         .......... ...LVVDDEP VNLRVLDSF- -LR------- L----EG---
101        --YR------ --VQTAK--D GLEAFEK--- ---------- ----------
151        -------L-- ---------- E----AE--- K--------- ------P---
201        -----E---- --L-VLLDIM M-P--G-M-- ---SG--YQV CE---K--L-
251        --R----Q-- ---------- ---------- ---------- ----------
301        ---------- ---------- ------D--- ---Y-----D ------HA--
351        Q--------- ---L--P--- ----V--IML T---ALN--- ----QADDRL
401        RGFEAGA--- ---NDYL-SK PFNKQEL--- ---------- AARIRAHL..
451        .......... .......... .......... .......... ..........
501        ......
//
ID   A4XWPyyy   ami; 115 BP.
XX
AC   ARB_CA63CD02;
OS   491776688_WP_005593238_1_724_838_histidine_kinase_Vibrio_
```

```
XX
XX
XX
XX
XX
XX
FT   source          1..115
XX
SQ   Sequence 115 BP;
1          .......... .......... .......... .......... ..........
51         .......... ...LVVDDEP VNLRVLDSF- -LR------- I----AG---
101        --YR------ --VRTAK--D GFEALQQ--- ---------- ----------
151        -------I-- ---------- E----QE--- R--------- ------P---
201        -----E---- --L-VLLDVM M-P--G-L-- ---SG--YQV CE---T--I-
251        --R----E-- ---------- ---------- ---------- ----------
301        ---------- ---------- ------N--- ---Y-----S ------QS--
351        E--------- ---L--P--- ----V--IML T---ALN--- ----QPDDRI
401        RGFNSGA--- ---NDYL-SK PFNKQEL--- ---------- AARILVHL..
451        .......... .......... .......... .......... ..........
501        ......
//
ID   A53WPyyy   ami; 115 BP.
XX
AC   ARB_E68862C2;
OS
WP_006001166_1_720_834_multi_sensor_hybrid_histidine_kinase_Desulfurom
onas_acetoxidans_
XX
XX
XX
XX
XX
XX
FT   source          1..115
XX
SQ   Sequence 115 BP;
1          .......... .......... .......... .......... ..........
51         .......... ...LVVDDEP VNLQVLVNQ- -LH------- V----AR---
101        --YR------ --VRIAN--S GQQALDL--- ---------- ----------
151        -------V-- ---------- E----EE--- T--------- ------P---
201        -----D---- --L-ILLDIM M-P--H-M-- ---TG--YEV CQ---R--L-
251        --R----R-- ---------- ---------- ---------- ----------
301        ---------- ---------- ------T--- ---Y-----N ------AA--
351        Q--------- ---L--P--- ----V--IML T---ARS--- ----RVSDVV
401        QGFQAGA--- ---NDYV-AK PFSRDIL--- ---------- LARVRTQL..
451        .......... .......... .......... .......... ..........
501        ......
//
ID   A58WPyyy   ami; 115 BP.
XX
```

```
AC   ARB_8635D2F7;
OS   WP_006210711_1_664_778_histidine_kinase_Paenibacillus_vortex_
XX
XX
XX
XX
XX
XX
FT   source          1..115
XX
SQ   Sequence 115 BP;
1         .......... .......... .......... .......... ..........
51        .......... ...LIVDDDP INVQVLLQF- -LG------- -----GK---
101       --YS------ --HRSTC--S GQEALEW--- ---------- ----------
151       -------I-- ---------- E----NGY-- K--------- ------P---
201       -----D---- --I-ALLDVM M-P--Y-V-- ---SG--LQL GE---E--I-
251       --R----K-- ---------- ---------- ---------- ----------
301       ---------- ---------- ------H--- ---Y-----N ------SG--
351       E--------- ---L--P--- ----I--LFL S---AKS--- ----QMTDLV
401       AGFEHGG--- ---NDYL-AK PVAKQEL--- ---------- LARLELHL..
451       .......... .......... .......... .......... ..........
501       ......
//
ID   A59WPyyy   ami; 115 BP.
XX
AC   ARB_61C1726;
OS
493272788_WP_006230602_1_712_826_histidine_kinase_Photobacterium_profu
ndum_
XX
XX
XX
XX
XX
XX
FT   source          1..115
XX
SQ   Sequence 115 BP;
1         .......... .......... .......... .......... ..........
51        .......... ...LVVDDEP VNLQVLNNF- -LR------- L----EG---
101       --YR------ --VLTAD--S GPQALKI--- ---------- ----------
151       -------I-- ---------- E----QQ--- Q--------- ------P---
201       -----A---- --L-ILLDIM M-P--E-M-- ---SG--YEV CH---I--L-
251       --R----E-- ---------- ---------- ---------- ----------
301       ---------- ---------- ------E--- ---H-----S ------LI--
351       E--------- ---L--P--- ----I--IML S---ALG--- ----QVQDRI
401       KGFESGA--- ---NDYL-TK PFNKEEL--- ---------- IARISAHL..
451       .......... .......... .......... .......... ..........
501       ......
//
```

```
ID   A5TWPyyy   ami; 113 BP.
XX
AC   ARB_10DD558A;
OS   WP_007129051_1_724_836_histidine_kinase_Paenibacillus_lactis_
XX
XX
XX
XX
XX
XX
FT   source          1..113
XX
SQ   Sequence 113 BP;
1         .......... .......... .......... .......... ..........
51        .......... ...LAVDDDP VNLKVLLGI- -LS------- S----EP---
101       --YH------ --ITTAV--S GLEVLEL--- ---------- ----------
151       -------L-- ---------- G----MR--- T--------- ------W---
201       -----D---- --L-LIADVM M-P--Q-M-- ---SG--YEL TQ---R--V-
251       --R----E-- ---------- ---------- ---------- ----------
301       ---------- ---------- ------H--- ---Y-----S ------VA--
351       E--------- ---L--P--- ----I--LLL T---ARS--- ----QPEDIY
401       TGFASGA--- ---NDYM-TK PVDATEL--- ---------- KYRIRA....
451       .......... .......... .......... .......... ..........
501       ......
//
ID   A61WPyyy   ami; 115 BP.
XX
AC   ARB_D21A6CA9;
OS
494735221_WP_007470661_1_723_837_Chitin_catabolic_cascade_sensor_histi
dine_kinase_ChiS_Photobacterium_sp_AK15_
XX
XX
XX
XX
XX
XX
FT   source          1..115
XX
SQ   Sequence 115 BP;
1         .......... .......... .......... .......... ..........
51        .......... ...LIVDDEP VNLQVLTNY- -LR------- L----EG---
101       --YR------ --VITAE--N GPQALEL--- ---------- ----------
151       -------L-- ---------- E----SE--- Q--------- ------P---
201       -----A---- --L-LLLDIM M-P--G-M-- ---NG--YEV CQ---Q--L-
251       --R----K-- ---------- ---------- ---------- ----------
301       ---------- ---------- ------E--- ---H-----T ------PL--
351       S--------- ---L--P--- ----I--IML S---ALG--- ----QVQDRV
401       KGFEYGA--- ---NDYL-TK PFNKEEL--- ---------- TARIRAHI..
451       .......... .......... .......... .......... ..........
```

```
501       ......
//
ID   A6YWPyyy   ami; 115 BP.
XX
AC   ARB_E528EC38;
OS   WP_009592651_1_666_780_histidine_kinase_Paenibacillus_sp_HGF5_
XX
XX
XX
XX
XX
XX
FT   source          1..115
XX
SQ   Sequence 115 BP;
1         .......... .......... .......... .......... ..........
51        .......... ...LIVDDDP INVQVLLQF- -LA------- -----GK---
101       --YS------ --LRSTC--S GQEALEW--- ---------- ----------
151       -------I-- ---------- Q----NGY-- K--------- ------P---
201       -----D---- --I-ALLDVM M-P--Y-V-- ---SG--LQL GE---E--I-
251       --R----R-- ---------- ---------- ---------- ----------
301       ---------- ---------- ------H--- ---Y-----N ------SG--
351       E--------- ---L--P--- ----V--IFL S---AKS--- ----QMTDLI
401       AGFEHGG--- ---NDYL-AK PVDKQEL--- ---------- LARLELHL..
451       .......... .......... .......... .......... ..........
501       ......
//
ID   A7HWPyyy   ami; 114 BP.
XX
AC   ARB_87949D26;
OS
WP_010651340_1_685_798_ATPase_domain_containing_protein_Oceanobacillus
massiliensis str. N'diop
XX
XX
XX
XX
XX
XX
FT   source          1..114
XX
SQ   Sequence 114 BP;
1         .......... .......... .......... .......... ..........
51        .......... ...LIADDEI VNVQVLRNH- -LS------- T----VG---
101       --YW------ --LEVAY--D GESALEI--- ---------- ----------
151       -------L-- ---------- T----EDP-- S--------- ------Y---
201       -----D---- --L-VILDVM L-P--K-Q-- ---SG--FEI AK---R--L-
251       --R----K-- ---------- ---------- ---------- ----------
301       ---------- ---------- ------R--- ---Y-----S ------LT--
351       E--------- ---L--P--- ----I--LML T---ARS--- ----QTEDIV
```

```
401       TAFESGA--- ---NDYL-TK PCSKEEL--- ---------- LSRVKT....
451       .......... .......... .......... .......... ..........
501       ......
//
ID   A7WWPyyy   ami; 115 BP.
XX
AC   ARB_F030FDE6;
OS
WP_017463536_1_1146_1260_hypothetical_protein_Dyella_ginsengisoli_
XX
XX
XX
XX
XX
XX
FT   source          1..115
XX
SQ   Sequence 115 BP;
1         .......... .......... .......... .......... ..........
51        .......... ...LVVDDEP INRQVLRNH- -LL------- L----ED---
101       --YH------ --VVEAS--G GEEALAA--- ---------- ----------
151       -------F-- ---------- A----DT--- T--------- ------P---
201       -----D---- --L-VLLDVM M-P--R-M-- ---SG--FEV CR---Q--L-
251       --R----Q-- ---------- ---------- ---------- ----------
301       ---------- ---------- ------A--- ---H-----R ------PA--
351       D--------- ---L--P--- ----V--IYL S---ART--- ----QLADRL
401       SGFESGA--- ---SDYL-TK PVAKAEL--- ---------- VARVRTHL..
451       .......... .......... .......... .......... ..........
501       ......
//
ID   A31WYyy3   ami; 116 BP.
XX
AC   ARB_C4DD5AE6;
OS
WP_019029155_1_1103_1218_hypothetical_protein_Colwellia_piezophila_BAA
-637
XX
XX
XX
XX
XX
XX
FT   source          1..116
XX
SQ   Sequence 116 BP;
1         .......... .......... .......... .......... ..........
51        .......... ...LLVDDEP INRQVLHNH- -LS------- M----QD---
101       --YQ------ --LVEVS--G GEQALQA--- ---------- ----------
151       -------I-- ---------- S----EQG-- P--------- ------F---
201       -----D---- --L-VLLDIM M-P--K-V-- ---SG--YEV CK---I--L-
```

```
251       --R----L-- ---------- ---------- ---------- ----------
301       ---------- ---------- ------S--- ---H-----G ------IN--
351       D--------- ---L--P--- ----V--IFL T---AKN--- ----QVNDLV
401       HSFAVGA--- ---NDYL-SK PVSKLEL--- ---------- LARVETQL..
451       .......... .......... .......... .......... ..........
501       ......
//
ID   Y30Pyyyy   ami; 115 BP.
XX
AC   ARB_11FBE448;
OS
54307731_YP_128751_1_726_840_sensory_box_sensor_histidine_kinase_respo
nse_regulator_Photobacterium_profundum_SS9_
XX
XX
XX
XX
XX
XX
FT   source          1..115
XX
SQ   Sequence 115 BP;
1         .......... .......... .......... .......... ..........
51        .......... ...LVVDDEP VNLQVLNNF- -LR------- L----EG---
101       --YR------ --VLTAD--S GPQALKI--- ---------- ----------
151       -------I-- ---------- E----QQ--- Q--------- ------P---
201       -----A---- --L-ILLDIM M-P--E-M-- ---SG--YEV CH---I--L-
251       --R----E-- ---------- ---------- ---------- ----------
301       ---------- ---------- ------E--- ---Y-----S ------LI--
351       E--------- ---L--P--- ----I--IML S---ALG--- ----QVQDRI
401       KGFESGA--- ---NDYL-TK PFNKEEL--- ---------- IARISAHL..
451       .......... .......... .......... .......... ..........
501       ......
//
ID   YuriYyy4   ami; 115 BP.
XX
AC   ARB_31C22FCE;
OS
YP_722386_1_720_834_adenylate_guanylate_cyclase_Trichodesmium_erythrae
um_IMS101_
XX
XX
XX
XX
XX
XX
FT   source          1..115
XX
SQ   Sequence 115 BP;
1         .......... .......... .......... .......... ..........
```

```
51        .......... ....IVDDEP VNLQVIRNN- -LA------- L----QN---
101       --YA------ --ITEAN--N GIEALEM--- ---------- ----------
151       -------I-- ---------- E----KGL-- M--------- ------P---
201       -----D---- --L-ILLDVM M-P--H-M-- ---TG--YEV AK---K--L-
251       --R----E-- ---------- ---------- ---------- ----------
301       ---------- ---------- ------K--- ---Y-----L ------HI--
351       E--------- ---L--P--- ----I--IML T---AKP--- ----LVSDLV
401       EGFVSGA--- ---NDYL-IK PFSKQEL--- ---------- LARIKIHI..
451       .......... .......... .......... .......... ..........
501       ......
//
ID   Y70Ptyyy   ami; 115 BP.
XX
AC   ARB_10B98241;
OS
YP_007087963_1_701_815_transmembrane_sensor_domain_containing_protein_
Oscillatoria_acuminata_PCC_6304_
XX
XX
XX
XX
XX
XX
FT   source          1..115
XX
SQ   Sequence 115 BP;
1         .......... .......... .......... .......... ..........
51        .......... ....IVDDEP INLQVLANT- -LS------- L----QN---
101       --YA------ --ITRAA--N GLKALEI--- ---------- ----------
151       -------I-- ---------- E----NGF-- K--------- ------P---
201       -----D---- --L-ILLDVM M-P--R-M-- ---TG--YEV CE---K--I-
251       --R----Q-- ---------- ---------- ---------- ----------
301       ---------- ---------- ------K--- ---F-----A ------AI--
351       D--------- ---L--P--- ----I--VML T---AKN--- ----QVSDLV
401       EGFSAGA--- ---NDYL-MK PFNKNEL--- ---------- LTRIKTHI..
451       .......... .......... .......... .......... ..........
501       ......
//
ID   YPdmnY10   ami; 115 BP.
XX
AC   ARB_4A101136;
OS
YP_007119567_1_884_998_PAS_domain_containing_protein_Microcoleus_sp_PC
C_7113_
XX
XX
XX
XX
XX
XX
```

```
FT   source          1..115
XX
SQ   Sequence 115 BP;
1         .......... .......... .......... .......... ..........
51        .......... .....IVDDEP VNLQVLINH- -LS------- L----QN---
101       --YA------ --ITQAS--N GIDALAM--- ---------- ----------
151       -------I-- ---------- E----QGF-- K--------- ------P---
201       -----D---- --I-VLLDVM M-P--R-M-- ---TG--YEV CQ---K--L-
251       --R----E-- ---------- ---------- ---------- ----------
301       ---------- ---------- ------K--- ---F-----P ------AY--
351       E--------- ---L--P--- ----V--VML T---AKN--- ----QVTDLV
401       EGFSVGA--- ---NDYL-SK PISKNEL--- ---------- LARIKTHL..
451       .......... .......... .......... .......... ..........
501       ......
//
ID   A99WPYyy   ami; 116 BP.
XX
AC   ARB_AC73696F;
OS
WP_002682771_1_687_802_signal_transduction_histidine_kinase_Beggiatoa_
alba_
XX
XX
XX
XX
XX
XX
FT   source          1..116
XX
SQ   Sequence 116 BP;
1         .......... .......... .......... .......... ..........
51        .......... ...LIVDDDP INLQVLENQ- -LK------- L----EN---
101       --YG------ --VTRAA--N GMAALDA--- ---------- ----------
151       -------I-- ---------- N----SGT-- Y--------- ------F---
201       -----N---- --L-ILLDIM M-P--R-M-- ---SG--FEV CR---I--I-
251       --R----E-- ---------- ---------- ---------- ----------
301       ---------- ---------- ------K--- ---W-----G ------AN--
351       E--------- ---L--P--- ----I--IML T---AKN--- ----QVSDLV
401       DGLQAGA--- ---NDYL-SK PFSKNEL--- ---------- LTRIKIHI..
451       .......... .......... .......... .......... ..........
501       ......
//
ID   A9CWPyyy   ami; 116 BP.
XX
AC   ARB_C39FD367;
OS
WP_002684462_1_706_821_PAS_domain_S_box_diguanylate_cyclase_GGDEF_doma
in_containing_protein_Beggiatoa_alba_
XX
XX
```

```
XX
XX
XX
XX
FT   source          1..116
XX
SQ   Sequence 116 BP;
1          .......... .......... .......... .......... ..........
51         .......... ...LLVDDEL INLHVLSNY- -LT------- P----HG---
101        --YE------ --LIHAT--S GHEVLQI--- ---------- ----------
151        -------L-- ---------- E----DGV-- K--------- ------P---
201        -----D---- --L-ILLDIM M-P--K-M-- ---TG--YEV CK---K--V-
251        --R----A-- ---------- ---------- ---------- ----------
301        ---------- ---------- ------R--- ---Y-----N ------AN--
351        E--------- ---L--P--- ----I--III S---AKN--- ----QVNDLV
401        SGLNAGA--- ---NDYL-VK PVWKDEL--- ---------- LARMRIHL..
451        .......... .......... .......... .......... ..........
501        ......
//
ID   W45PYyy2   ami; 115 BP.
XX
AC   ARB_6DD11D8E;
OS
WP_006098419_1_981_1095_PAS_fold_family_Coleofasciculus_chthonoplastes
_
XX
XX
XX
XX
XX
XX
FT   source          1..115
XX
SQ   Sequence 115 BP;
1          .......... .......... .......... .......... ..........
51         .......... ....IVDDDT VNRQILVNY- -LS------- L----EN---
101        --YQ------ --LAQAA--N GREALDI--- ---------- ----------
151        -------I-- ---------- N----NGF-- Q--------- ------P---
201        -----D---- --L-ILLDAI M-P--R-M-- ---TG--YDV CK---K--L-
251        --R----E-- ---------- ---------- ---------- ----------
301        ---------- ---------- ------F--- ---Y-----P ------PT--
351        E--------- ---L--P--- ----V--LML T---ANE--- ----QVNDLV
401        AGFSVGV--- ---NDYL-TK PIAKYKL--- ---------- LVRIKTHL..
451        .......... .......... .......... .......... ..........
501        ......
//
ID   AA8WPyyy   ami; 116 BP.
XX
AC   ARB_162D1DC6;
OS   WP_006621627_1_876_991_adenylate_cyclase_Arthrospira_platensis_
```

```
XX
XX
XX
XX
XX
XX
FT   source          1..116
XX
SQ   Sequence 116 BP;
1         .......... .......... .......... .......... ..........
51        .......... ...MIVDDDP INLQVLANY- -LY------- L----EN---
101       --YA------ --VTQAT--N GMEALKI--- ---------- ----------
151       -------L-- ---------- E----SGF-- I--------- ------P---
201       -----D---- --L-ILLDVM M-P--R-M-- ---TG--YEV TE---K--I-
251       --R----E-- ---------- ---------- ---------- ----------
301       ---------- ---------- ------D--- ---W-----P ------PH--
351       Q--------- ---L--P--- ----I--MML T---AKN--- ----RISDLV
401       VGLELGA--- ---NDYL-SK PLNKEEL--- ---------- LARIKTHI..
451       .......... .......... .......... .......... ..........
501       ......
//
ID   AARWPyyy   ami; 116 BP.
XX
AC   ARB_C6CFB276;
OS
WP_008473219_1_27_142_two_component_system_response_regulator_Beggiato
a_sp_SS_
XX
XX
XX
XX
XX
XX
FT   source          1..116
XX
SQ   Sequence 116 BP;
1         .......... .......... .......... .......... ..........
51        .......... ...LAVDDEP VNLQVVTNL- -LS------- L----EN---
101       --YT------ --VKTVM--G GAETFEL--- ---------- ----------
151       -------L-- ---------- E----QGE-- K--------- ------P---
201       -----D---- --L-ILLDIM M-P--K-M-- ---TG--YEV CQ---K--L-
251       --R----E-- ---------- ---------- ---------- ----------
301       ---------- ---------- ------Q--- ---Y-----S ------AT--
351       E--------- ---L--P--- ----I--MLL T---AKN--- ----QVSDLV
401       EGLNVGA--- ---NDYI-TK PVSKNEL--- ---------- LARIKTHL..
451       .......... .......... .......... .......... ..........
501       ......
//
ID   AAVWYyyy   ami; 116 BP.
XX
```

```
AC   ARB_AFDB71EF;
OS
WP_008476837_1_253_368_adenylate_guanylate_cyclase_Beggiatoa_sp_PS_
XX
XX
XX
XX
XX
XX
FT   source          1..116
XX
SQ   Sequence 116 BP;
1         .......... .......... .......... .......... ..........
51        .......... ...LIVDDDP INLQILENQ- -LR------- V----EN---
101       --YA------ --ITRAT--T GHDALKA--- ---------- ----------
151       -------L-- ---------- E----SGI-- P--------- ------F---
201       -----A---- --I-ILLDIM M-P--K-M-- ---SG--FEV CQ---I--I-
251       --R----Q-- ---------- ---------- ---------- ----------
301       ---------- ---------- ------T--- ---Y-----P ------AN--
351       Q--------- ---L--P--- ----I--IML T---AKN--- ----QVSDLV
401       QGMQAGA--- ---NDYL-TK PFSKGEL--- ---------- ITRIKTHI..
451       .......... .......... .......... .......... ..........
501       ......
//
ID   AAXWPYyy   ami; 116 BP.
XX
AC   ARB_ADFB41B1;
OS
WP_008477205_1_564_679_Response_regulator_receiver_protein_Beggiatoa_s
p_PS_
XX
XX
XX
XX
XX
XX
FT   source          1..116
XX
SQ   Sequence 116 BP;
1         .......... .......... .......... .......... ..........
51        .......... ...LIVDDEA VNLHVLANH- -LS------- L----NH---
101       --YA------ --TIQAA--S GREALSI--- ---------- ----------
151       -------L-- ---------- E----EGL-- I--------- ------P---
201       -----D---- --L-ILLDVM M-P--H-M-- ---TG--YEV TQ---K--I-
251       --R----E-- ---------- ---------- ---------- ----------
301       ---------- ---------- ------H--- ---W-----E ------AN--
351       E--------- ---L--P--- ----I--VLL T---AKN--- ----QVSDLV
401       MGLEVGA--- ---NDYL-IK PISKNEL--- ---------- LARIKTHL..
451       .......... .......... .......... .......... ..........
501       ......
```

```
//
ID   AAYWPyyy   ami; 116 BP.
XX
AC   ARB_E024134E;
OS
WP_008477452_1_727_842_sensory_box_sensor_histidine_kinase_response_re
gulator_Beggiatoa_sp_PS_
XX
XX
XX
XX
XX
XX
FT   source          1..116
XX
SQ   Sequence 116 BP;
1          .......... .......... .......... .......... ..........
51         .......... ...FVVDDEP VNIQVLINH- -LT------- L----QN---
101        --YA------ --ISIAS--N GLEALAQ--- ---------- ----------
151        -------V-- ---------- E----KGY-- K--------- ------P---
201        -----D---- --I-ILLDVM M-P--K-M-- ---TG--YEV CR---K--L-
251        --R----E-- ---------- ---------- ---------- ----------
301        ---------- ---------- ------V--- ---F-----P ------VN--
351        E--------- ---L--P--- ----I--VML T---AKN--- ----QVSDLV
401        EGLESGA--- ---NDYL-TK PISKHEL--- ---------- IARLKMHL..
451        .......... .......... .......... .......... ..........
501        ......
//
ID   AAVWYyy2   ami; 104 BP.
XX
AC   ARB_8D6D71D6;
OS   WP_008478346_1_1_104_adenylate_guanylate_cyclase_Beggiatoa_sp_PS_
XX
XX
XX
XX
XX
XX
FT   source          1..104
XX
SQ   Sequence 104 BP;
1          .......... .......... .......... .......... ..........
51         .......... .......... .....MINH- -LS------- R----FN---
101        --YT------ --IIQAS--S GIEAWQL--- ---------- ----------
151        -------I-- ---------- E----NGL-- K--------- ------P---
201        -----D---- --V-ILLDVM M-P--Q-M-- ---TG--YEV TQ---K--I-
251        --R----E-- ---------- ---------- ---------- ----------
301        ---------- ---------- ------R--- ---F-----S ------AN--
351        E--------- ---L--P--- ----I--LML T---AKN--- ----QVSDLV
401        TGLEVGA--- ---NDYL-TK PISKYEL--- ---------- LARVKSHL..
```

```
451       .......... .......... .......... .......... ..........
501       ......
//
ID   AB1WPyyy   ami; 117 BP.
XX
AC   ARB_C60C6EA2;
OS   WP_008480732_1_1_117_adenylate_cyclase_Beggiatoa_sp_PS_
XX
XX
XX
XX
XX
XX
FT   source          1..117
XX
SQ   Sequence 117 BP;
1         .......... .......... .......... .......... ..........
51        .......... ...MIVDDEP VNRQVLVNH- -LS------- L----QN---
101       --YK------ --TIQAT--D GQEALNL--- ---------- ----------
151       -------VL- ---------- E----GEI-- K--------- ------P---
201       -----D---- --L-ILLDIM M-P--Y-K-- ---TG--YDV TQ---K--I-
251       --R----K-- ---------- ---------- ---------- ----------
301       ---------- ---------- ------K--- ---W-----P ------AD--
351       E--------- ---L--P--- ----I--ILL T---AKN--- ----QVSDLV
401       VGLEMGA--- ---NDYL-TK PVSKDEL--- ---------- LARIKTHL..
451       .......... .......... .......... .......... ..........
501       ......
//
ID   WfESFYy6   ami; 115 BP.
XX
AC   ARB_9B286CFB;
OS
WP_018398141_1_688_802_hypothetical_protein_filamentous_cyanobacterium
_ESFC_1_
XX
XX
XX
XX
XX
XX
FT   source          1..115
XX
SQ   Sequence 115 BP;
1         .......... .......... .......... .......... ..........
51        .......... ....IVDDDP VNRQVLLNY- -LA------- L----EN---
101       --YI------ --VTQAE--D GLEALNL--- ---------- ----------
151       -------L-- ---------- E----QGY-- R--------- ------P---
201       -----D---- --L-ILLDIM M-P--Q-M-- ---TG--YEA CQ---R--I-
251       --R----S-- ---------- ---------- ---------- ----------
301       ---------- ---------- ------Q--- ---Y-----S ------SG--
```

```
351       E--------- ---L--P--- ----I--VLL T---AKI--- ----QVSDLV
401       EGFDSGA--- ---NDYL-TK PISKKEL--- ---------- LARLKTHL..
451       .......... .......... .......... .......... ..........
501       ......
//
ID   BAgZZZZZ   ami; 116 BP.
XX
AC
686203607_BAP54740_1_772_887_response_regulator_receiver_protein_Thiop
loca_ingrica\;
OS
686203607_BAP54740_1_WP_045478691_772_887_response_regulator_receiver_
protein_Thioploca_ingrica\
XX
XX
XX
XX
XX
XX
FT   source          1..116
XX
SQ   Sequence 116 BP;
1         .......... .......... .......... .......... ..........
51        .......... ...MIVDDEP INLQVLTNH- -LT------- L----YH---
101       --YA------ --TIQAN--S GLEALKQ--- ---------- ----------
151       -------L-- ---------- E----DGI-- I--------- ------P---
201       -----D---- --L-ILLDVM M-P--H-L-- ---TG--YEV TQ---R--I-
251       --R----R-- ---------- ---------- ---------- ----------
301       ---------- ---------- ------K--- ---W-----E ------AN--
351       E--------- ---L--P--- ----I--VLL T---AKN--- ----QVADVI
401       TGFEVGA--- ---NDYL-IK PISKPEL--- ---------- LARIKTHL..
451       .......... .......... .......... .......... ..........
501       ......
//
ID   BPsZZZZZ   ami; 116 BP.
XX
AC
686206846_BAP57979_1_1124_1239_response_regulator_receiver_domain_cont
aining_protein_Thioploca_ingrica\;
OS
686206846_BAP57979_1_1124_1239_WP_045480560_response_regulator_receive
r_domain_containing_protein_Thioploca_ingrica
XX
XX
XX
XX
XX
XX
FT   source          1..116
XX
```

```
SQ   Sequence 116 BP;
1         .......... .......... .......... .......... ..........
51        .......... ...LIVDDDP VNRQVLINH- -LS------- L----HN---
101       --YA------ --VSQAT--S GQEALTL--- ---------- ----------
151       -------I-- ---------- V----NGP-- K--------- ------P---
201       -----D---- --L-ILLDVM M-P--H-L-- ---SG--YEV TQ---K--I-
251       --R----E-- ---------- ---------- ---------- ----------
301       ---------- ---------- ------Q--- ---W-----Q ------AN--
351       E--------- ---L--P--- ----V--LLL T---AKN--- ----QIVDLV
401       TGLEAGA--- ---NDYL-TK PVAKEEL--- ---------- LARIKTHL..
451       .......... .......... .......... .......... ..........
501       ......
//
ID   KHpZZZZ9   ami; 116 BP.
XX
AC
723292343_KHD11477_1_126_241_hypothetical_protein_OT06_10600_Candidatu
s_Thiomargarita_nelsonii\;
OS
723292343_KHD11477_1_126_241_hypothetical_protein_OT06_10600_Candidatu
s_Thiomargarita_nelsonii
XX
XX
XX
XX
XX
XX
FT   source          1..116
XX
SQ   Sequence 116 BP;
1         .......... .......... .......... .......... ..........
51        .......... ...LIVDDDP LTLELFMMY- -LS------- L----AE---
101       --YT------ --ISTAT--S APEALAK--- ---------- ----------
151       -------L-- ---------- K----EGL-- K--------- ------P---
201       -----D---- --L-VLLDVV M-P--V-M-- ---NG--YEL TL---K--I-
251       --R----E-- ---------- ---------- ---------- ----------
301       ---------- ---------- ------T--- ---W-----S ------AN--
351       E--------- ---L--P--- ----V--LLI S---AKT--- ----ETSDVV
401       SGLEIGA--- ---NDYI-CK PINRKEL--- ---------- LARIKTHL..
451       .......... .......... .......... .......... ..........
501       ......
//
ID   AKGZZZZZ   ami; 116 BP.
XX
AC
780107804_WP_045475695_1_712_827_guanylate_cyclase_Thioploca_ingrica\;
OS
780107804_WP_045475695_1_712_827_guanylate_cyclase_Thioploca_ingrica
XX
XX
```

```
XX
XX
XX
XX
FT   source          1..116
XX
SQ   Sequence 116 BP;
1          .......... .......... .......... .......... ..........
51         .......... ...LIVDDEP INLQVLINH- -LS------- L----HN---
101        --YS------ --ITQAM--N GLEALAI--- ---------- ----------
151        -------V-- ---------- E----KGY-- Q--------- ------P---
201        -----D---- --L-VLLDVM M-P--K-M-- ---TG--YEV CQ---K--L-
251        --R----E-- ---------- ---------- ---------- ----------
301        ---------- ---------- ------K--- ---F-----P ------IN--
351        E--------- ---L--P--- ----I--LML T---AKN--- ----QVSDLV
401        EGLDAGA--- ---NDYL-SK PVSKSEL--- ---------- MARIKMHL..
451        .......... .......... .......... .......... ..........
501        ......
//
ID   AKKZZZZZ   ami; 116 BP.
XX
AC
780115170_WP_045478459_1_352_467_hypothetical_protein_partial_Thioploc
a_ingrica\;
OS
780115170_WP_045478459_1_352_467_hypothetical_protein_partial_Thioploc
a_ingrica
XX
XX
XX
XX
XX
XX
FT   source          1..116
XX
SQ   Sequence 116 BP;
1          .......... .......... .......... .......... ..........
51         .......... ...LIVDDDP INLQVLENQ- -LR------- L----EN---
101        --YA------ --VTRAH--N GQEALTA--- ---------- ----------
151        -------I-- ---------- D----SGT-- Q--------- ------F---
201        -----V---- --I-ILLDIM M-P--K-M-- ---SG--FEV CR---I--I-
251        --R----E-- ---------- ---------- ---------- ----------
301        ---------- ---------- ------R--- ---Y-----S ------IT--
351        Q--------- ---L--P--- ----I--IML T---AKN--- ----QVSDLV
401        EGMQAGA--- ---NDYL-TK PFSKGEL--- ---------- ITRIKTHI..
451        .......... .......... .......... .......... ..........
501        ......
//
```
