# Supplementary material for: Visualizing Evolutionary Relationships of Multidomain Proteins: An Example from Receiver (REC) Domains of Sensor Histidine Kinases in the Candidatus Maribeggiatoa str. Orange Guaymas Draft Genome
Source: Front Microbiol. 2016 Nov 14;7:1780. doi: 10.3389/fmicb.2016.01780 (PMC5108060; doi:10.3389/fmicb.2016.01780)
Supplement: Supplementary file 9 [file Image1.PDF]

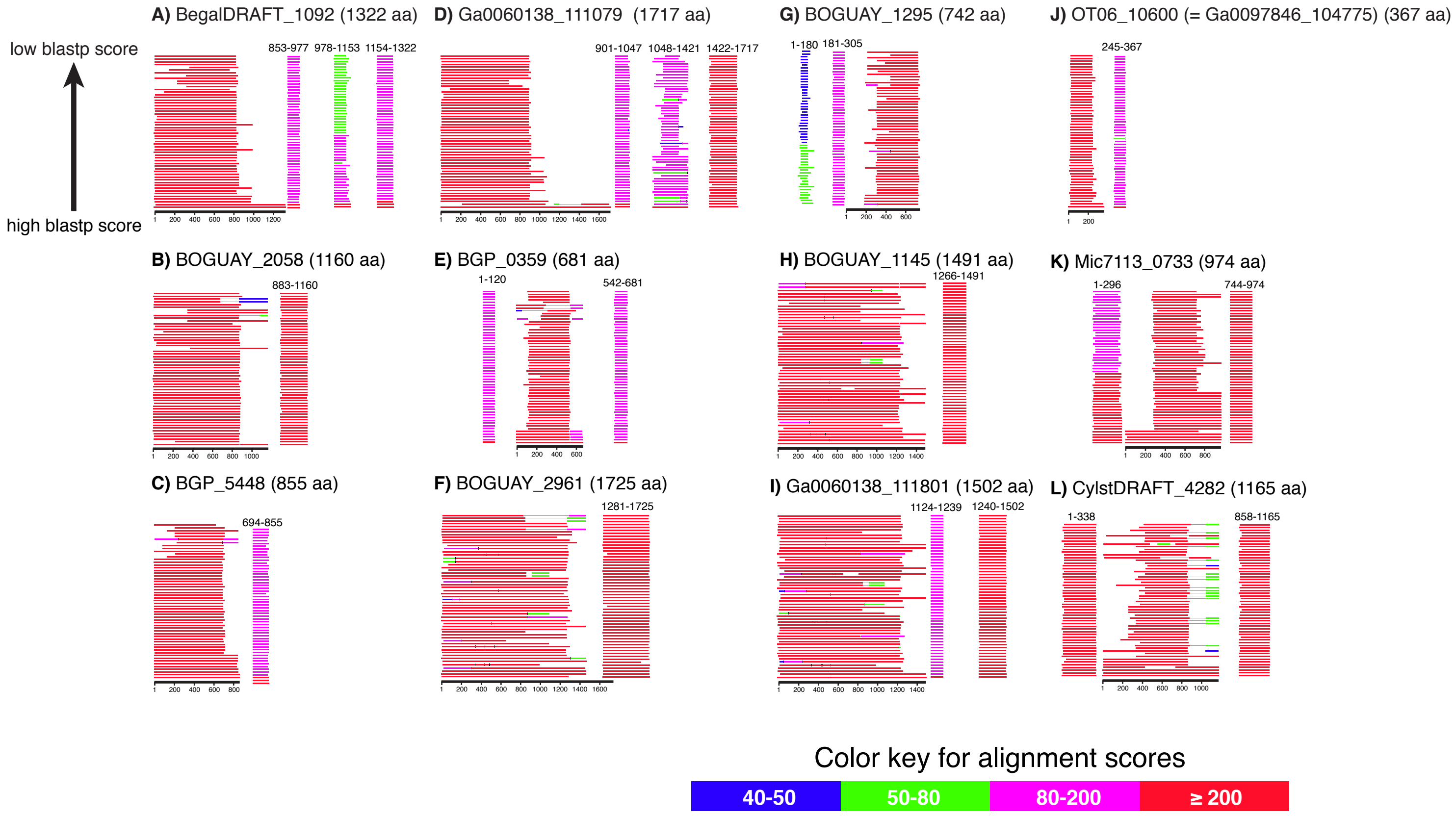

Supp. Fig. 1. BLASTP results for complete ORFs containing selected Tree 36 REC domains. Searches were carried out with default NCBI settings. Whole-ORF results are shown with a scale bar; segments identified by break points were then searched separately. Amino acid ranges covered by these are shown at the top of each diagram. High scores are at the bottom for consistency with other figures.
